# Supplementary material for: A Novel Class of Potent Anti-Tyrosinase Compounds with Antioxidant Activity, 2-(Substituted phenyl)-5-(trifluoromethyl)benzo[d]thiazoles: In Vitro and In Silico Insights
Source: Antioxidants (Basel). 2022 Jul 15;11(7):1375. doi: 10.3390/antiox11071375 (PMC9311798; doi:10.3390/antiox11071375)
Supplement: Supplementary file 1 [file antioxidants-11-01375-s001.zip › antioxidants-1785121-supplementary.pdf]

antioxidants

Supplementary Material

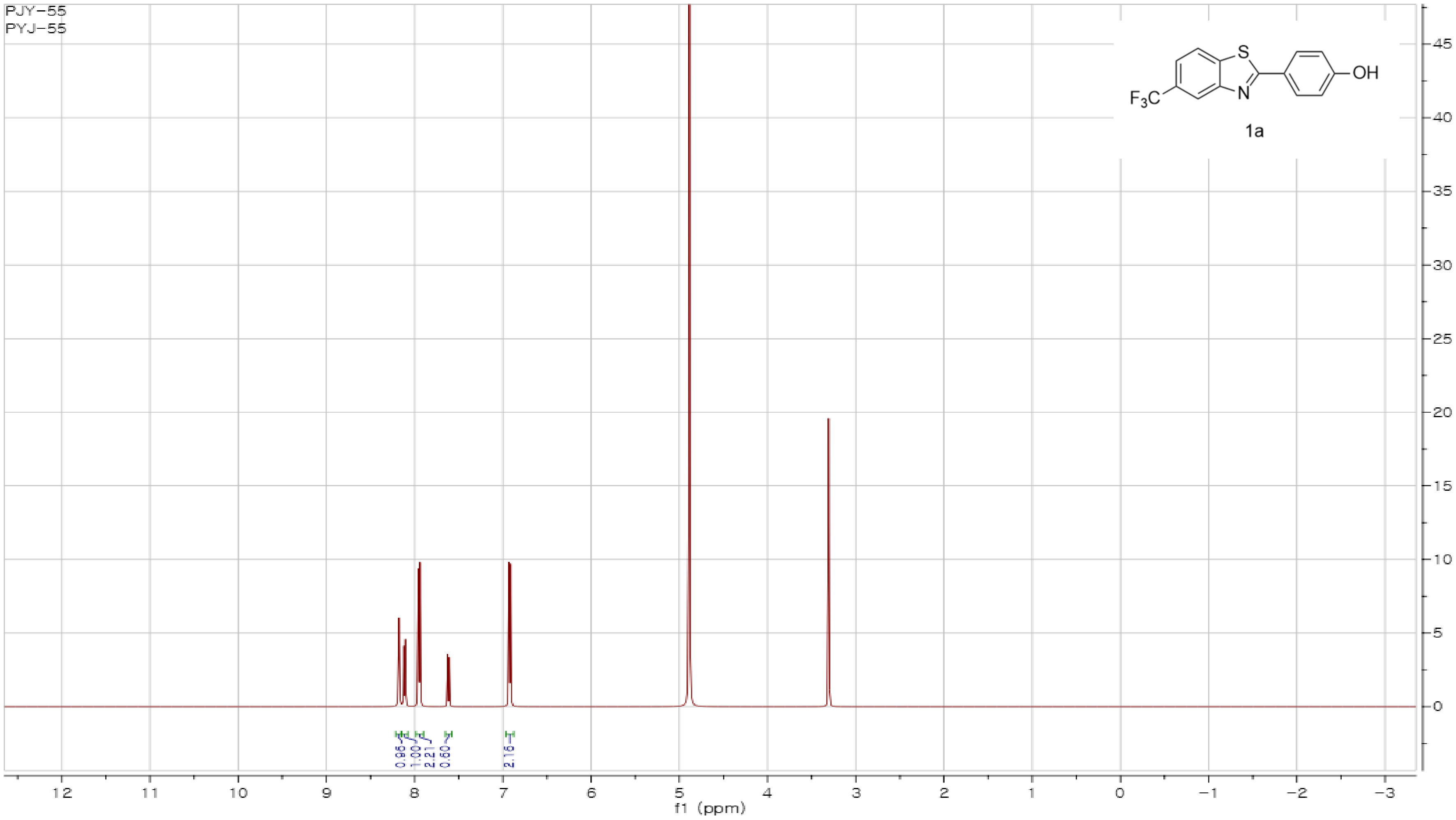

Figure S1. ^{1}H NMR spectrum of compound **1a.**

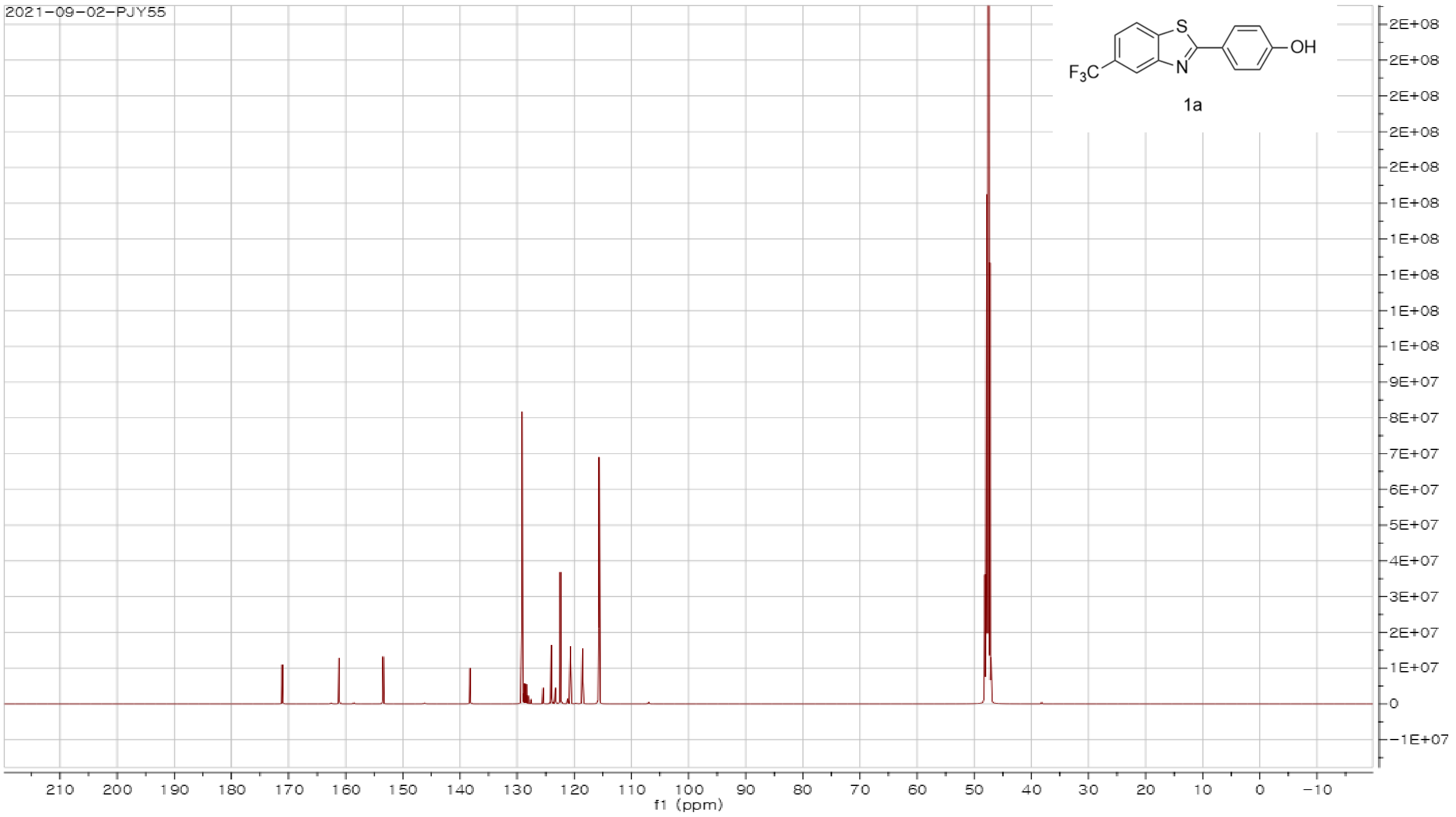

Figure S2. ^{13}C NMR spectrum of compound **1a.**

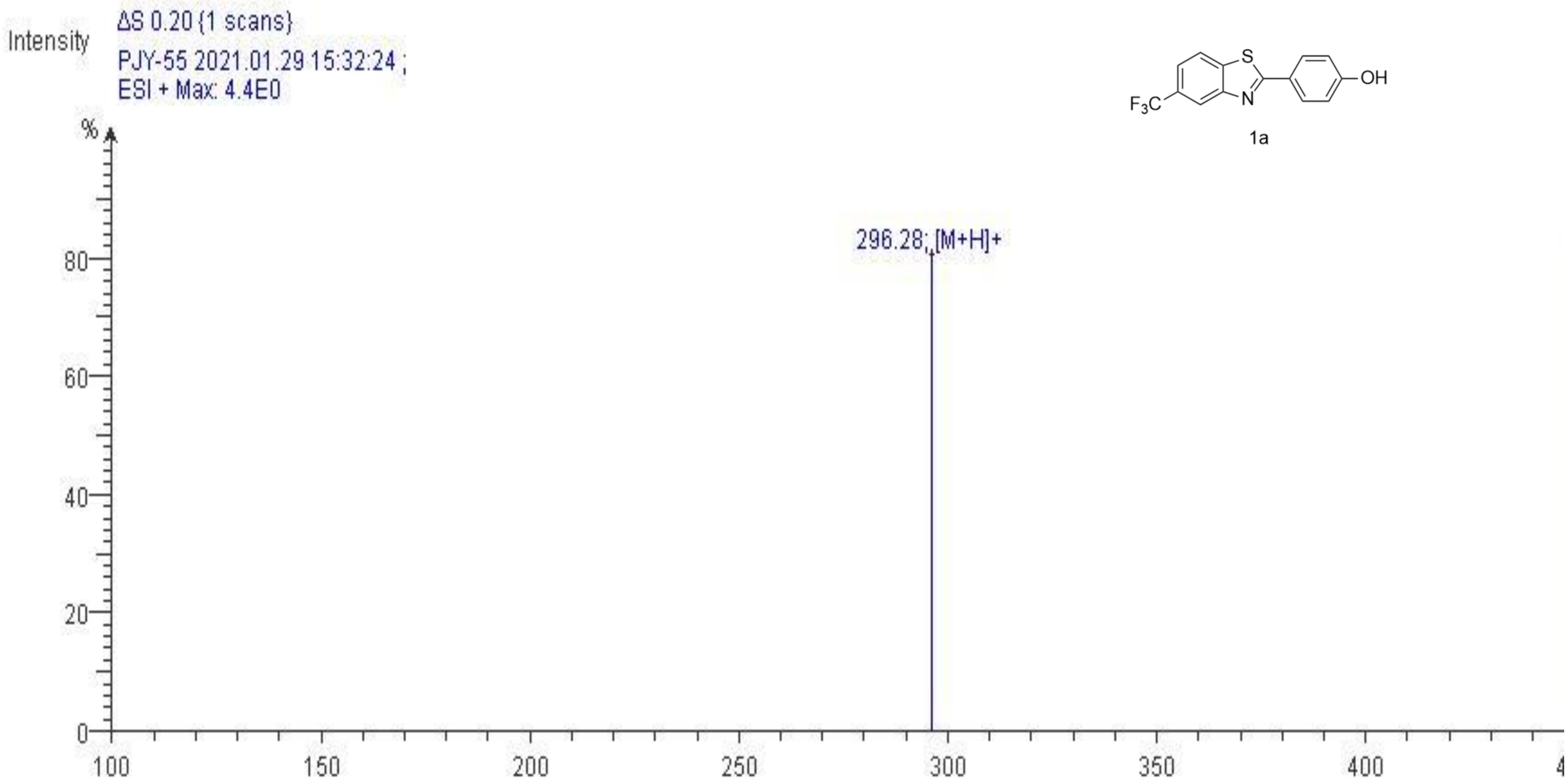

Figure S3. LRMS (ESI+) spectrum of compound **1a.**

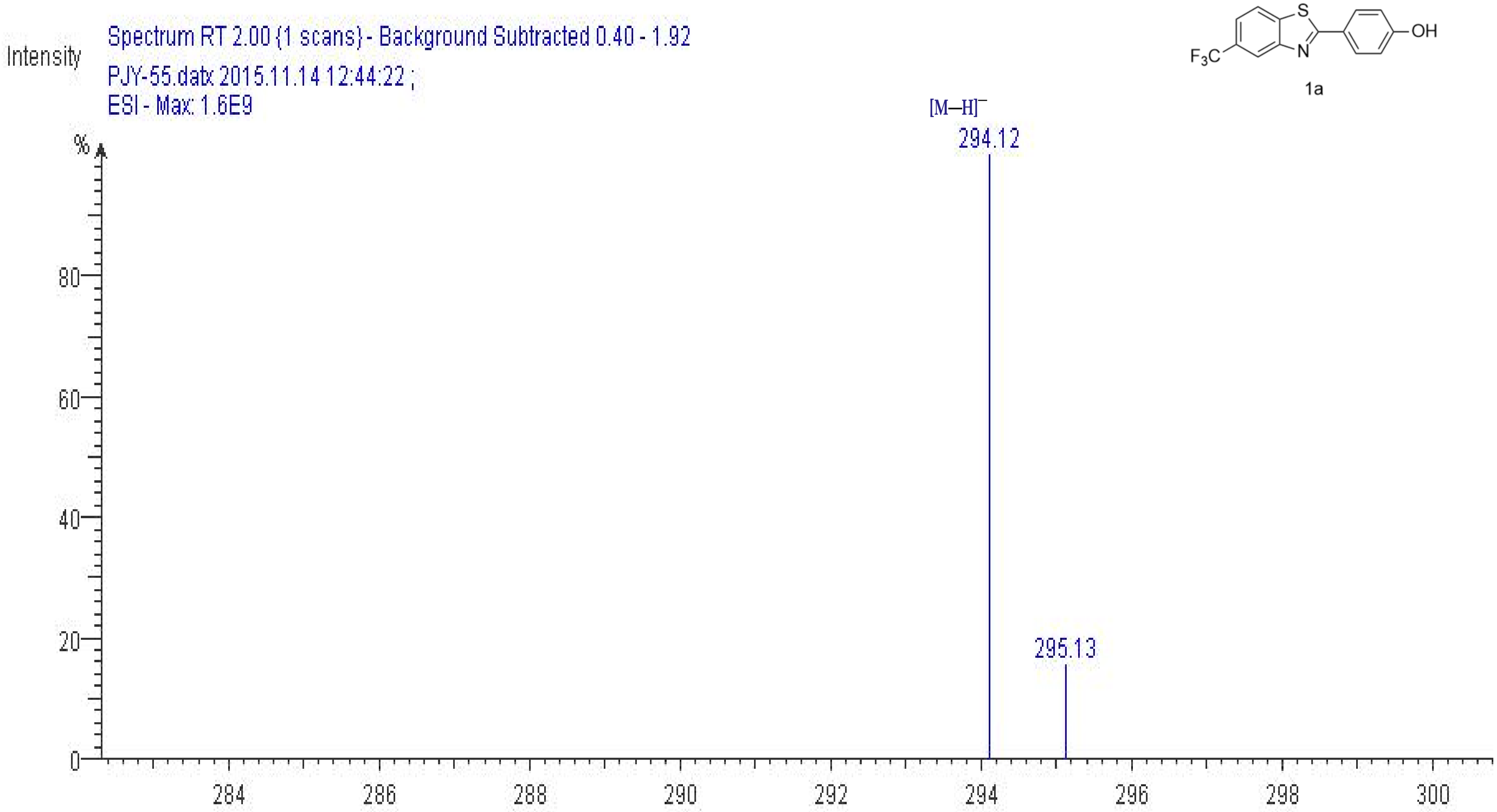

1a

Figure S4. LRMS (ESI–) spectrum of compound **1a.**

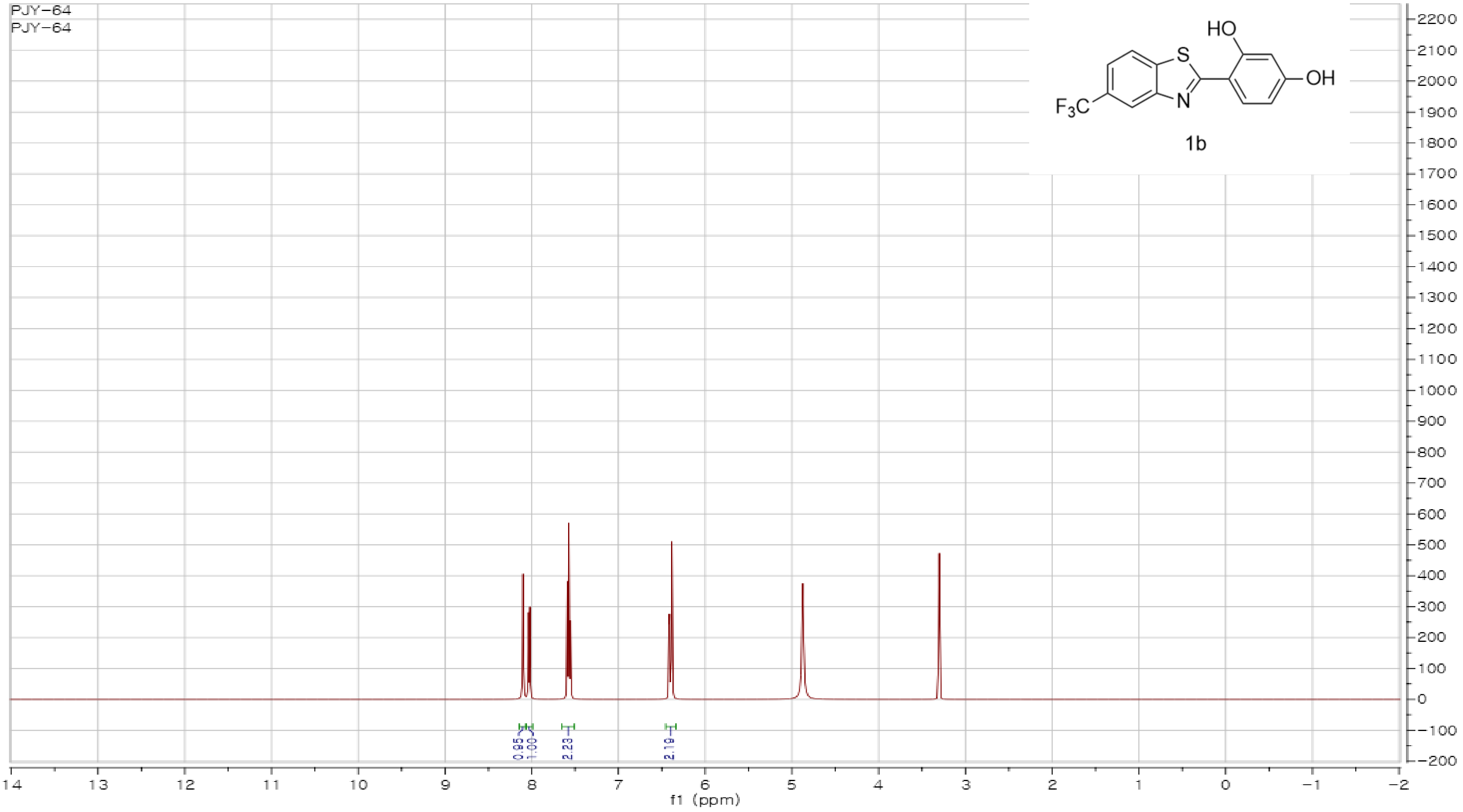

Figure S5. ^{1}H NMR spectrum of compound **1b.**

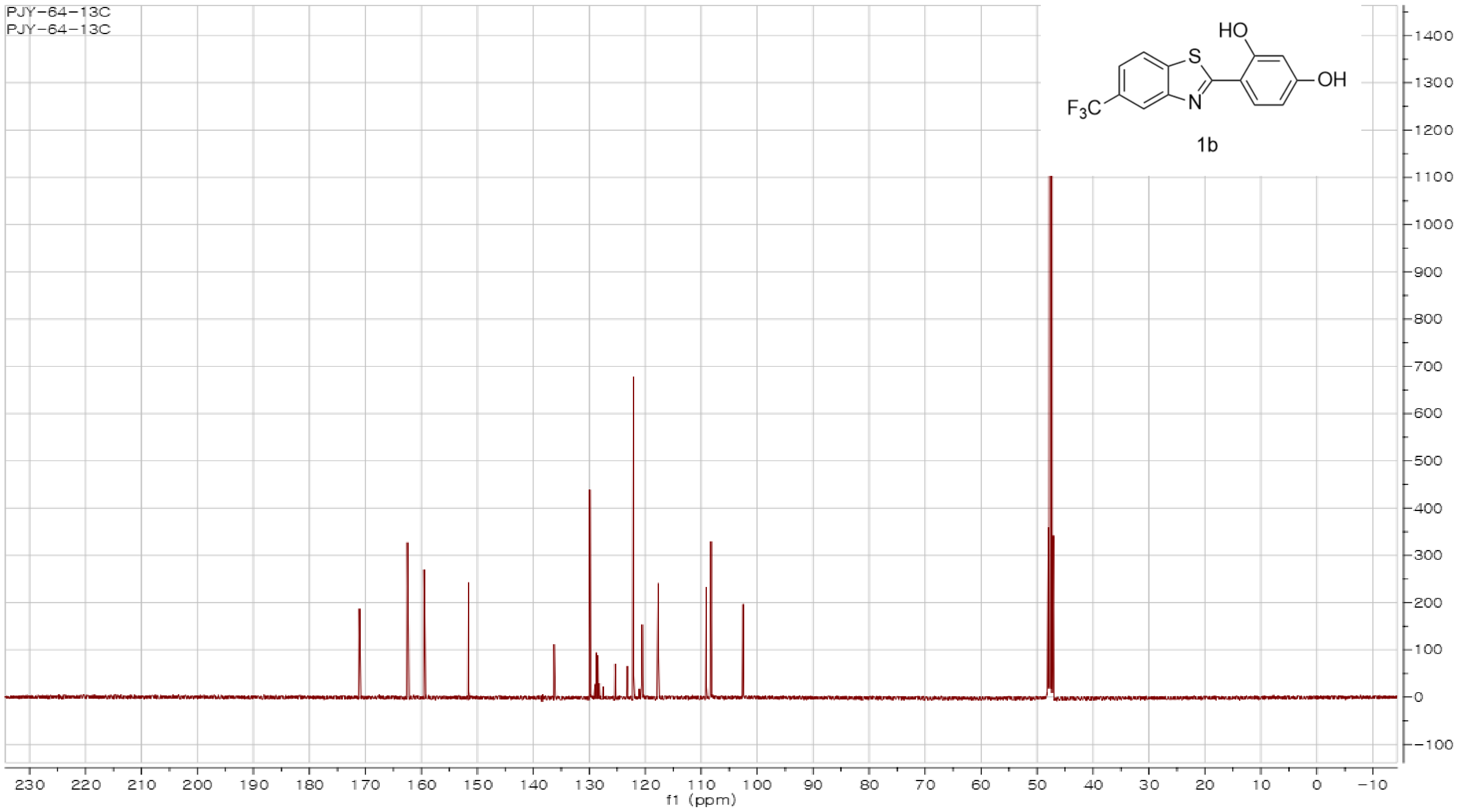

Figure S6. ^{13}C NMR spectrum of compound **1b.**

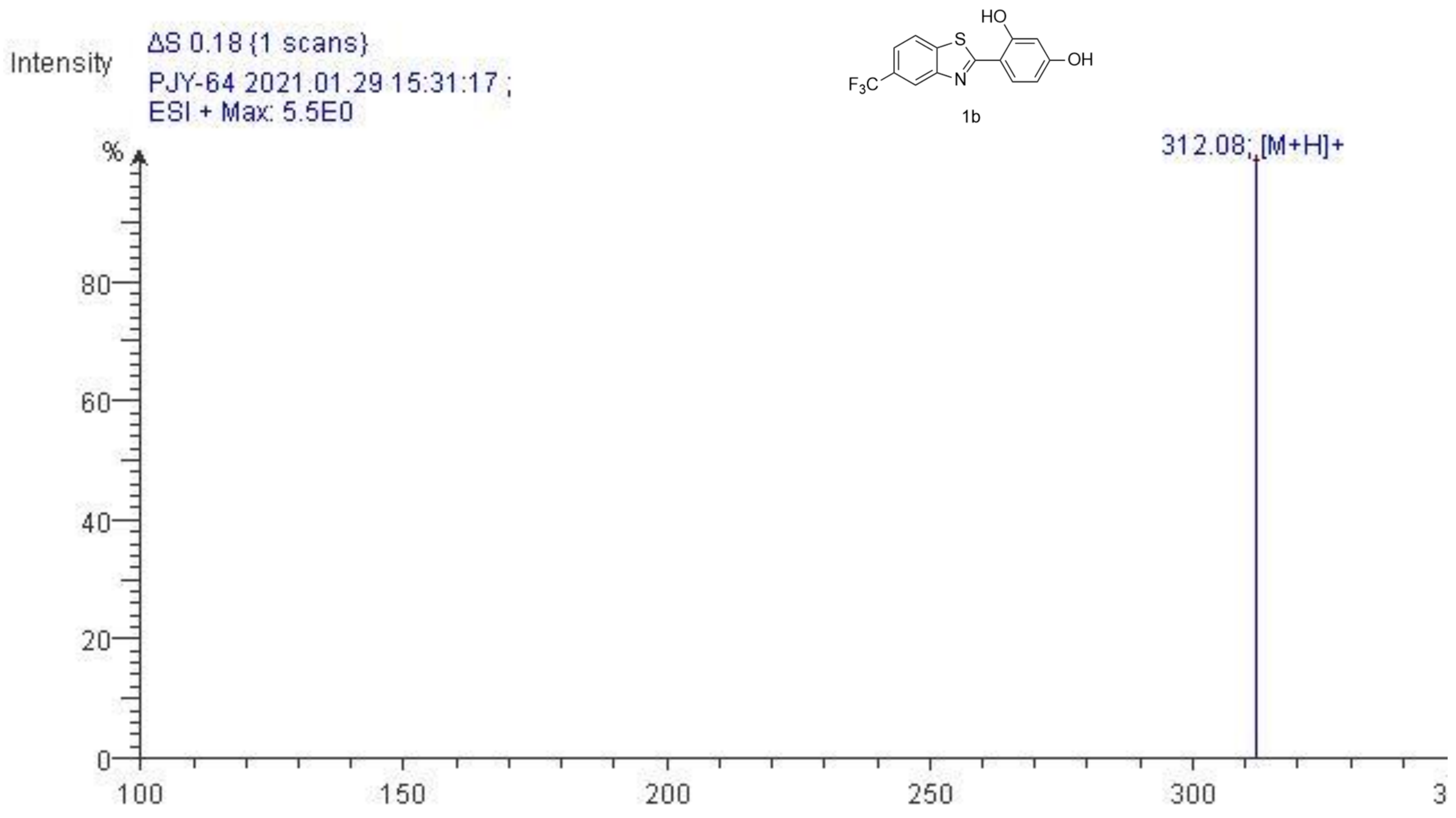

Figure S7. LRMS (ESI+) spectrum of compound **1b.**

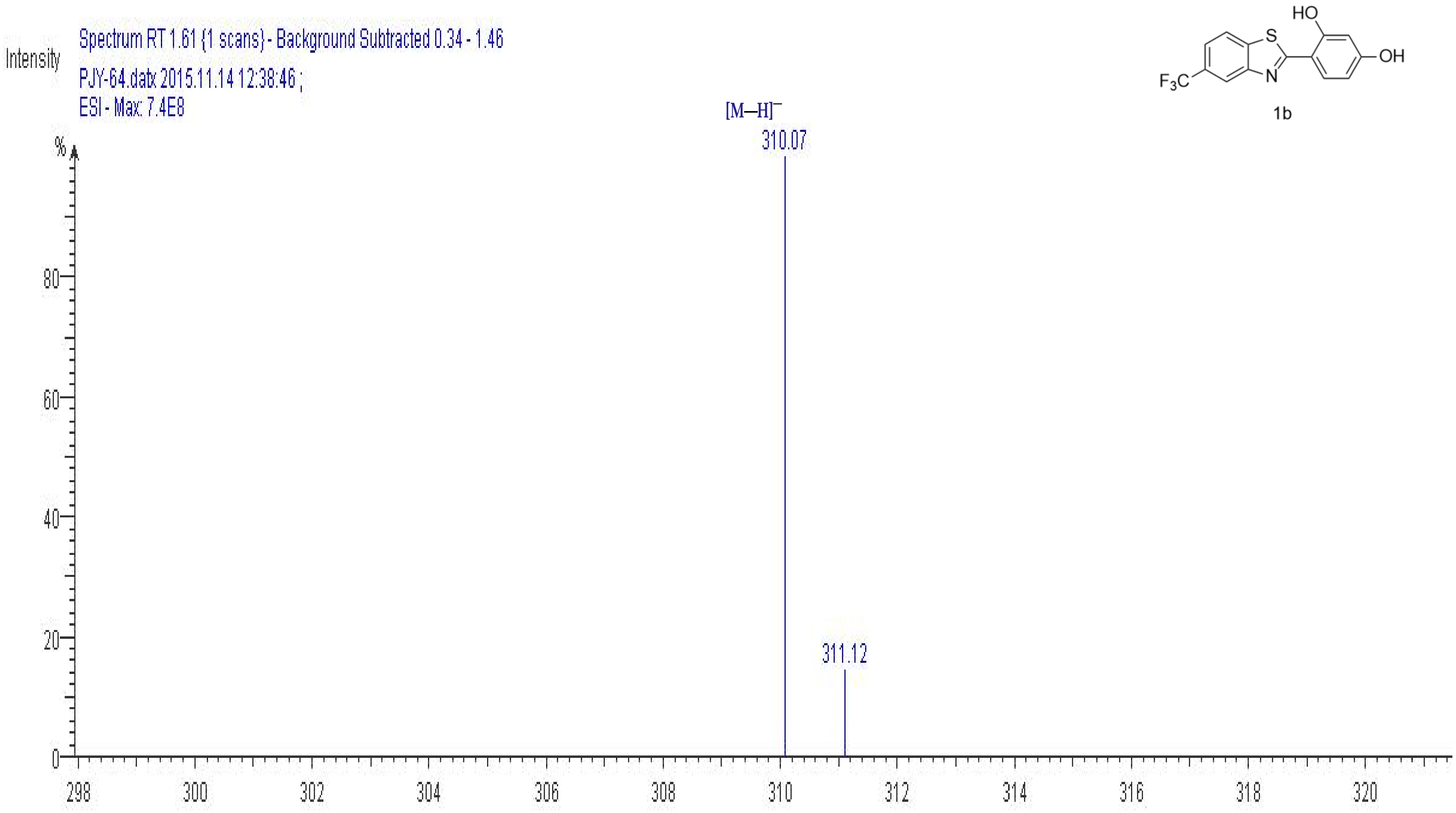

Figure S8. LRMS (ESI–) spectrum of compound **1b.**

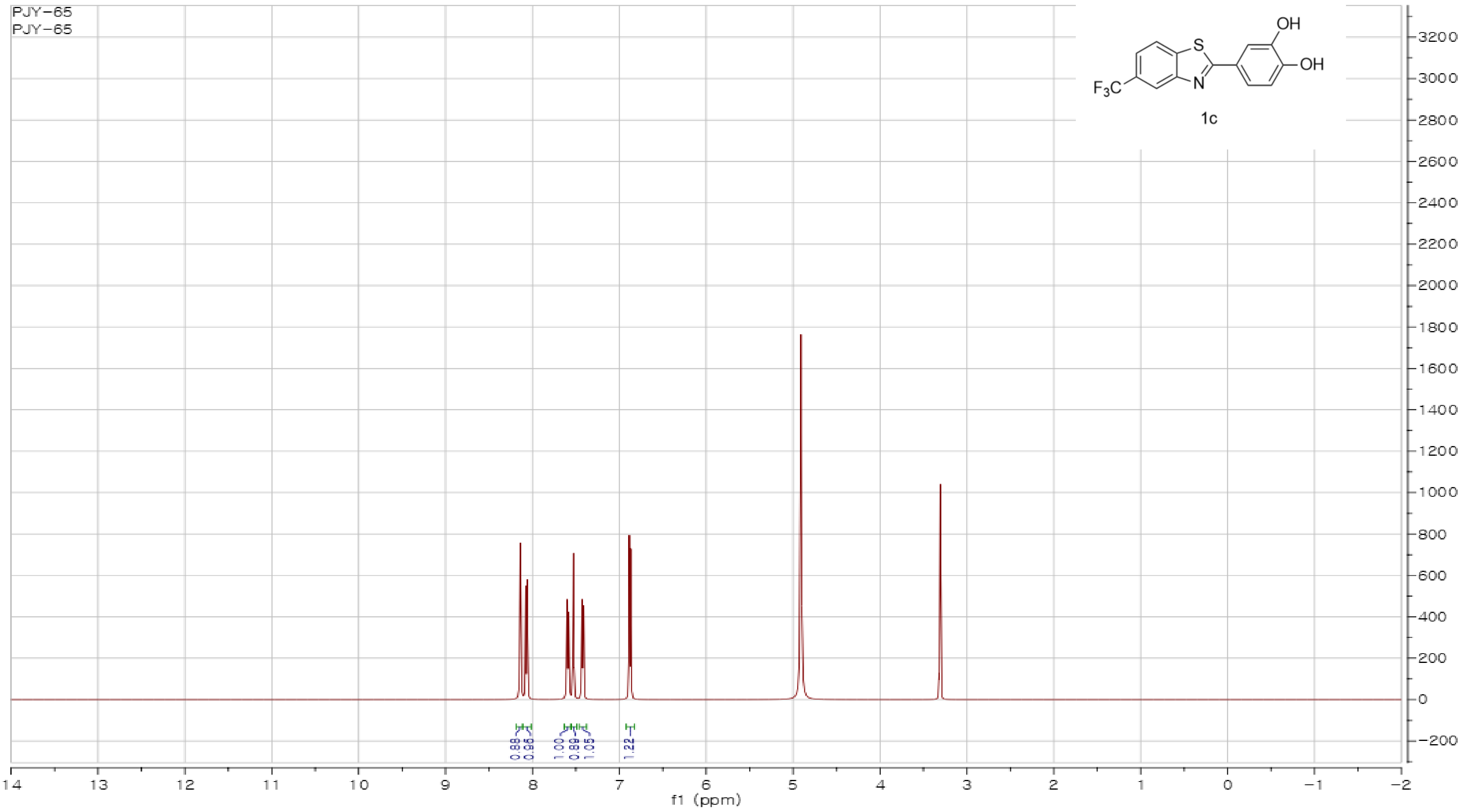

Figure S9. ^{1}H NMR spectrum of compound **1c.**

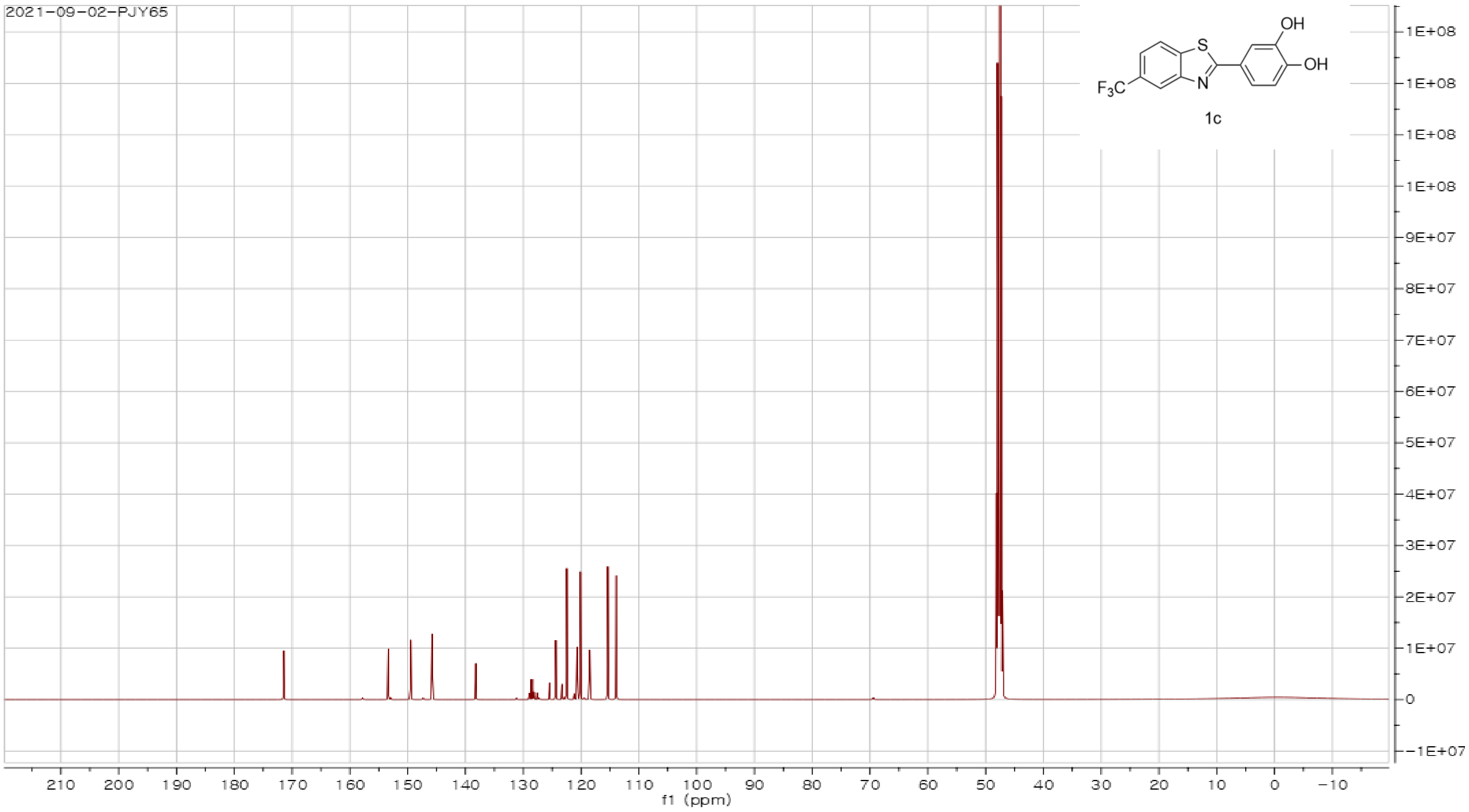

Figure S10. ^{13}C NMR spectrum of compound **1c.**

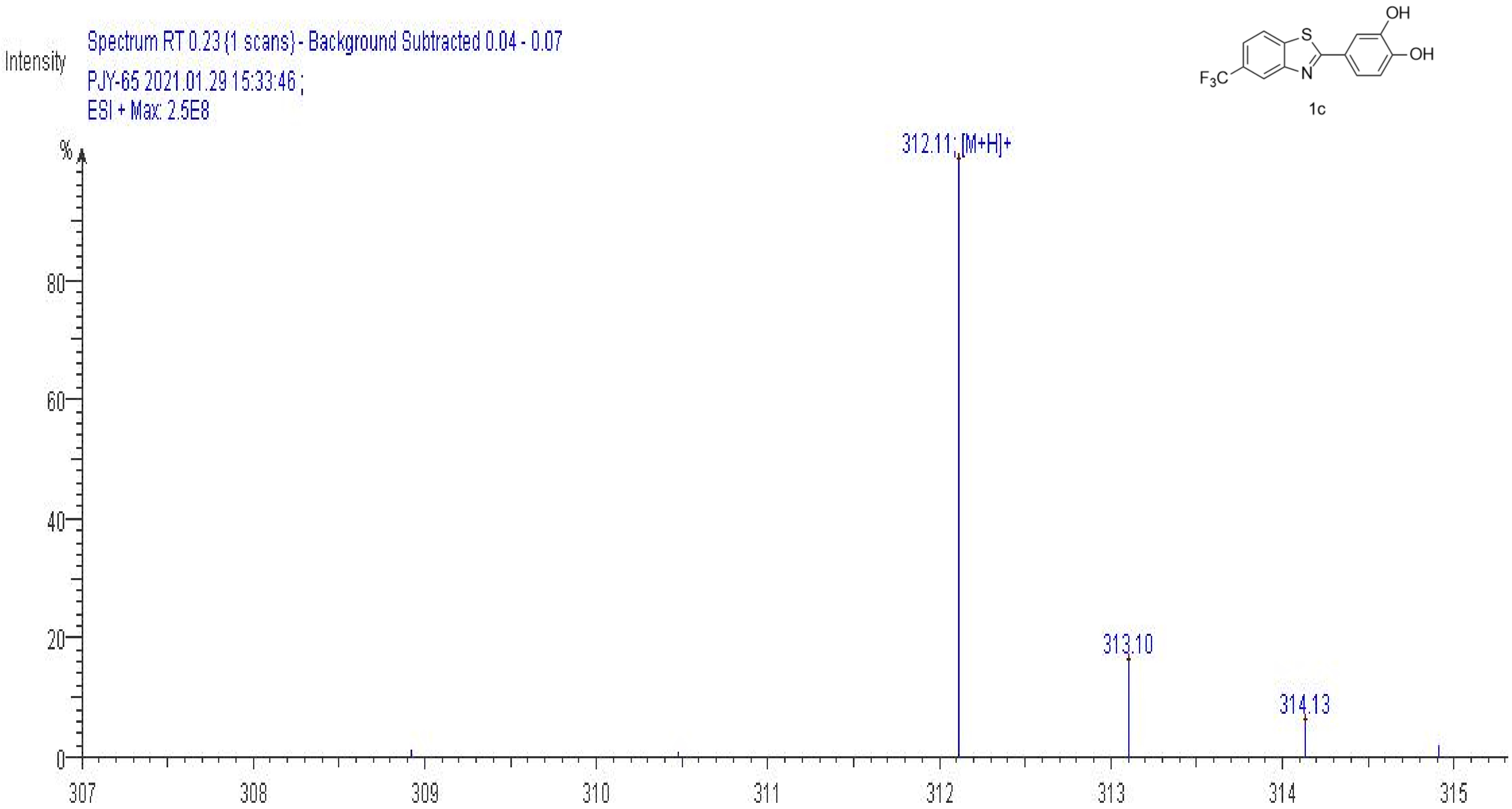

1c

Figure S11. LRMS (ESI+) spectrum of compound **1c.**

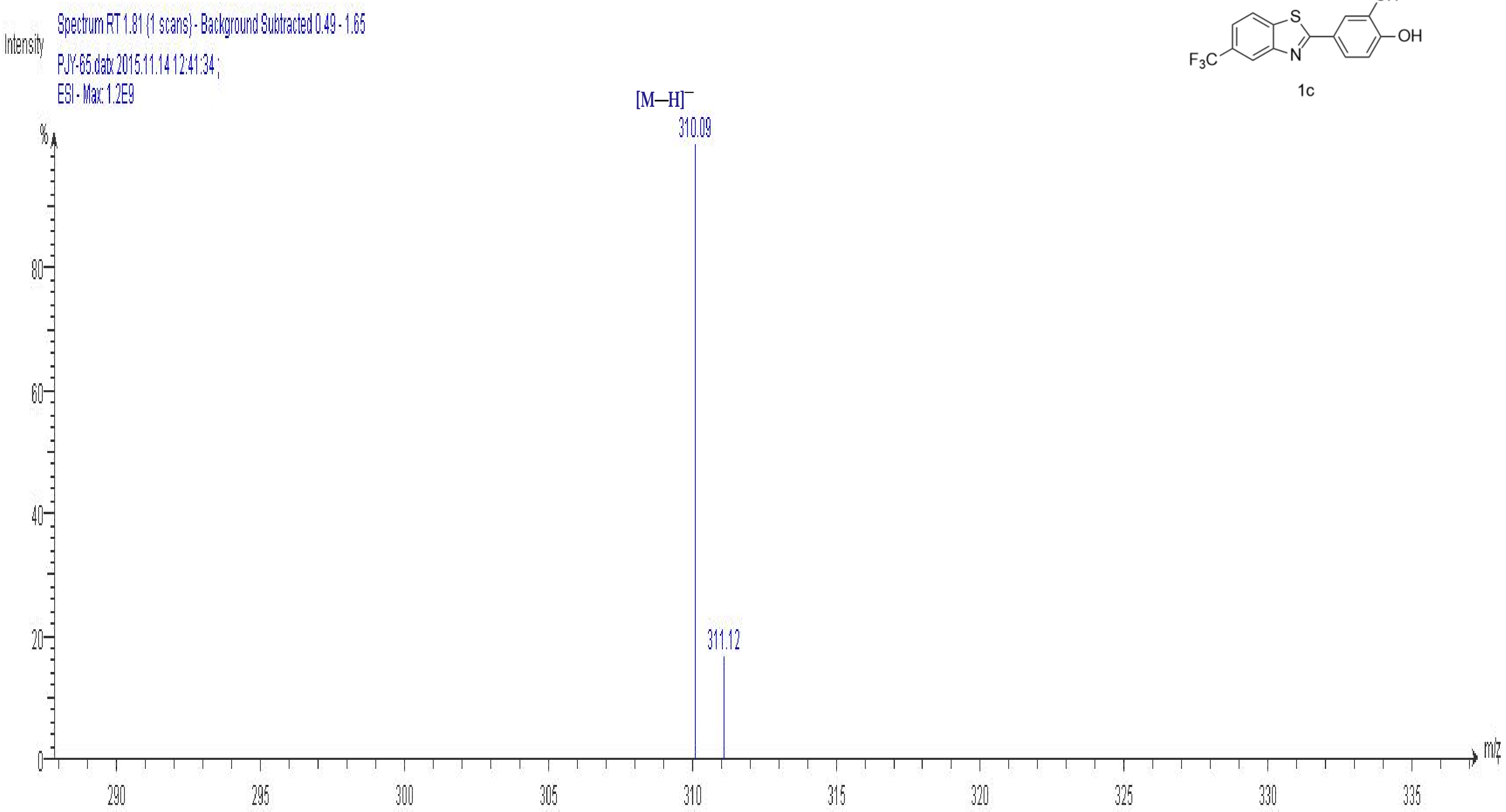

1c

Figure S12. LRMS (ESI–) spectrum of compound **1c.**

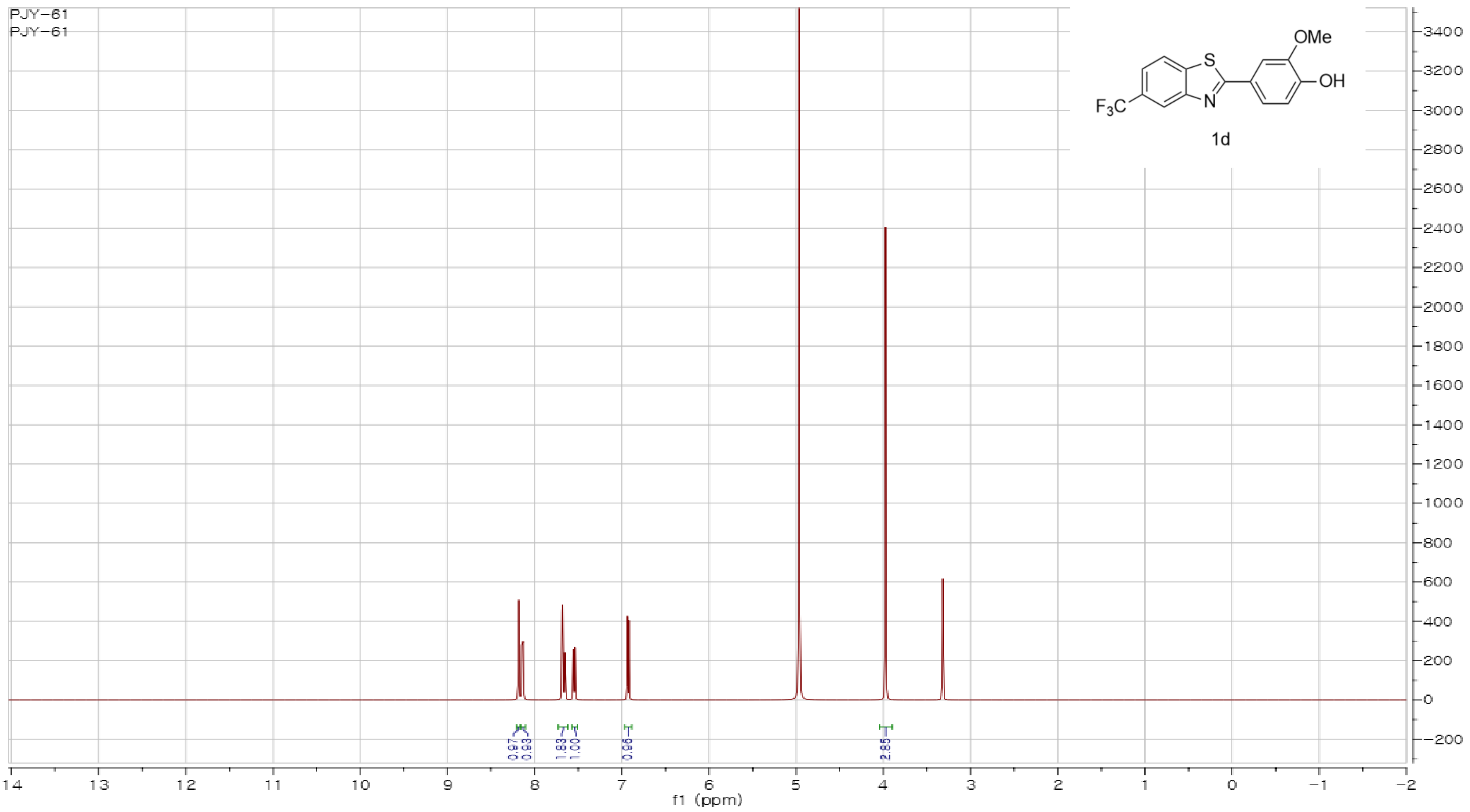

Figure S13. ^{1}H NMR spectrum of compound **1d.**

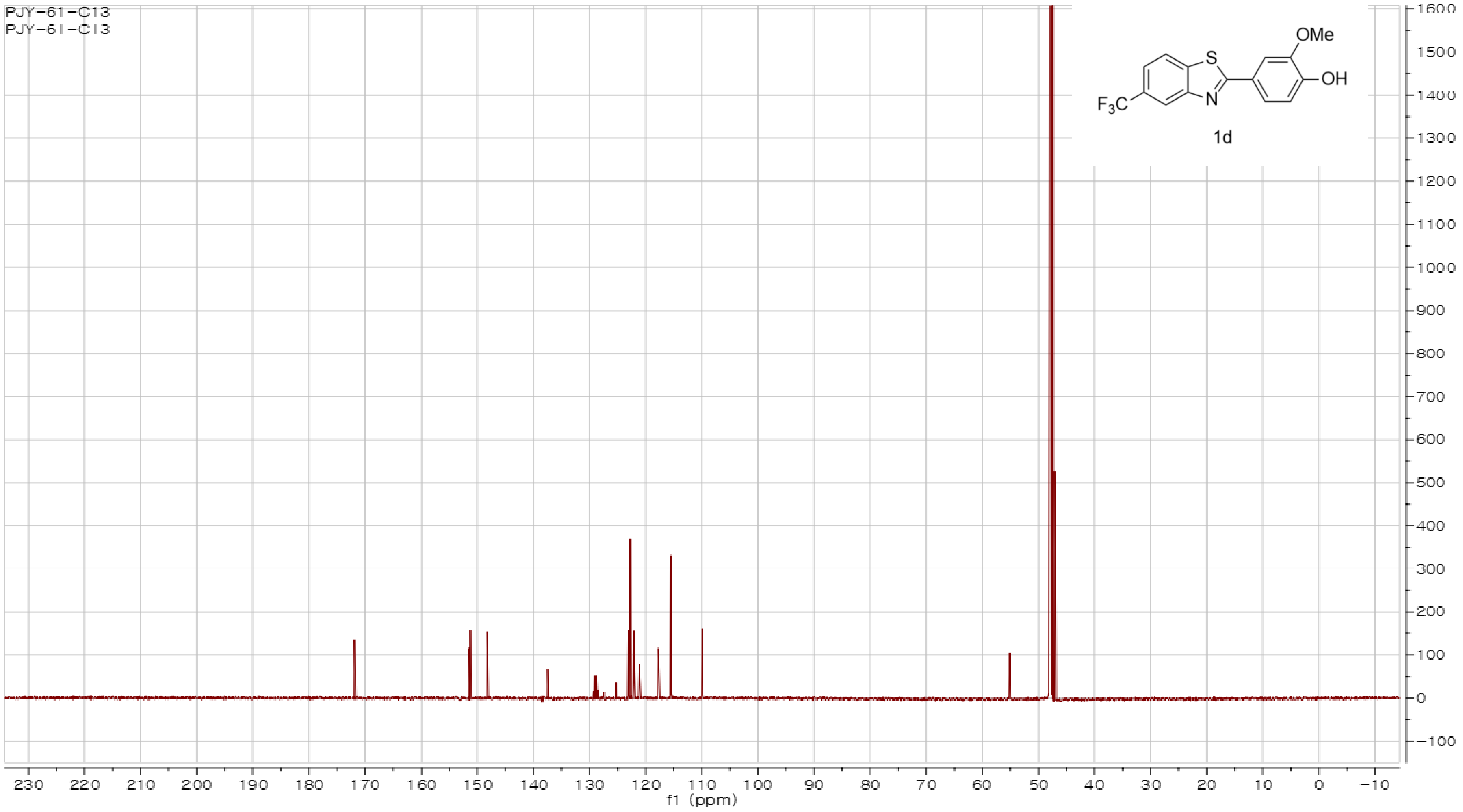

Figure S14. ^{13}C NMR spectrum of compound **1d.**

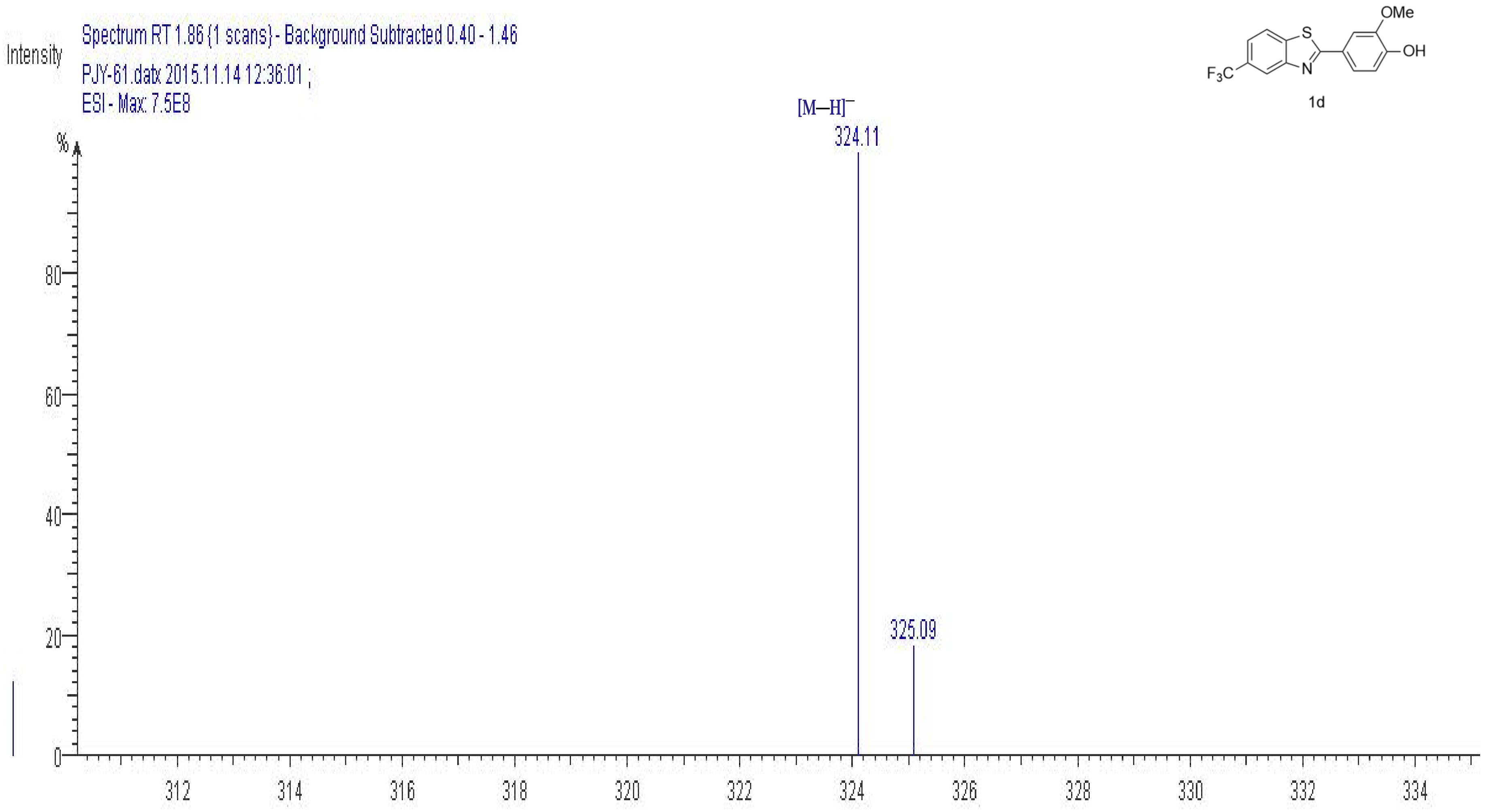

Figure S15. LRMS (ESI–) spectrum of compound **1d.**

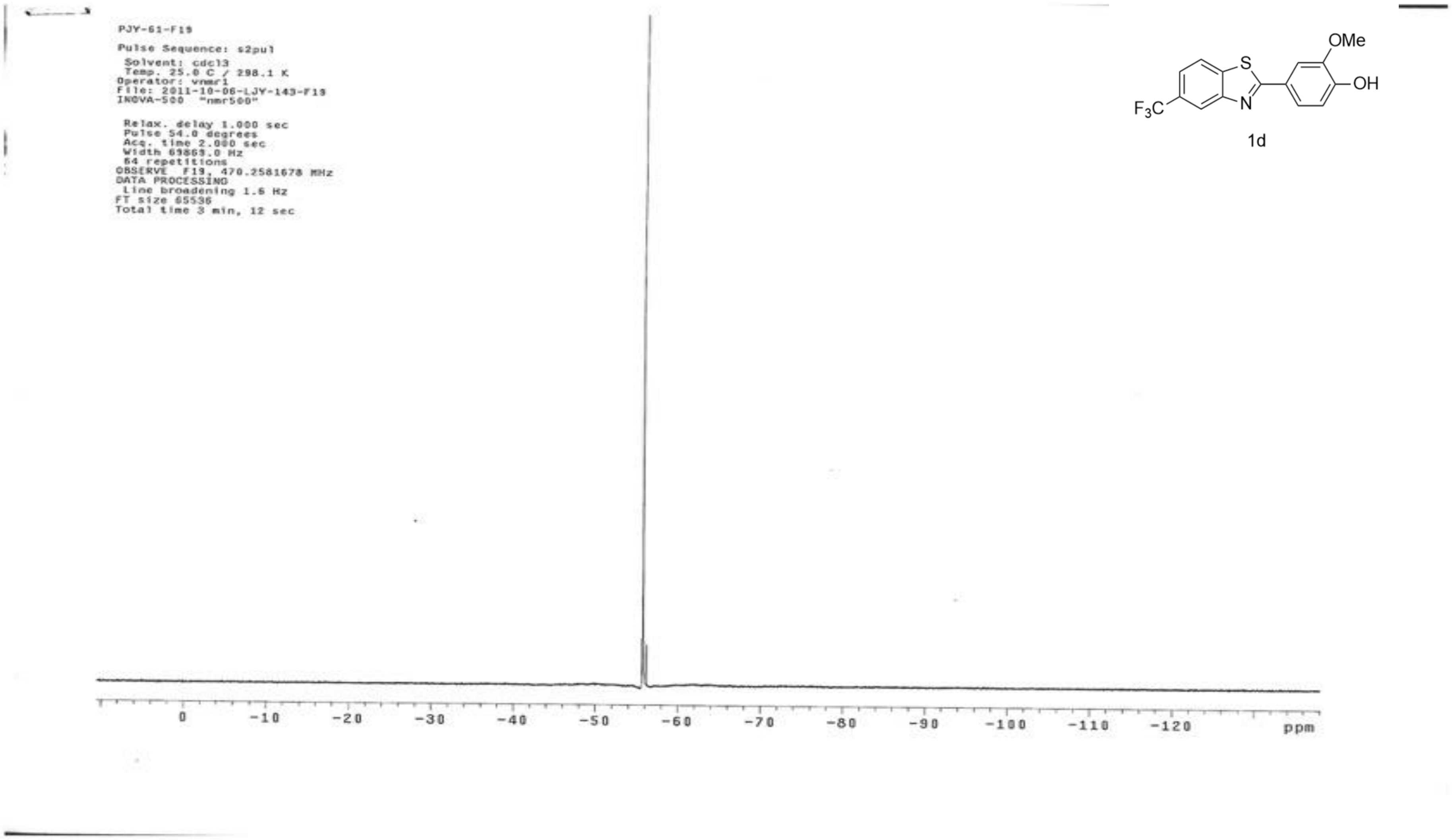

Figure S16. ^{19}F NMR spectrum of compound **1d.**

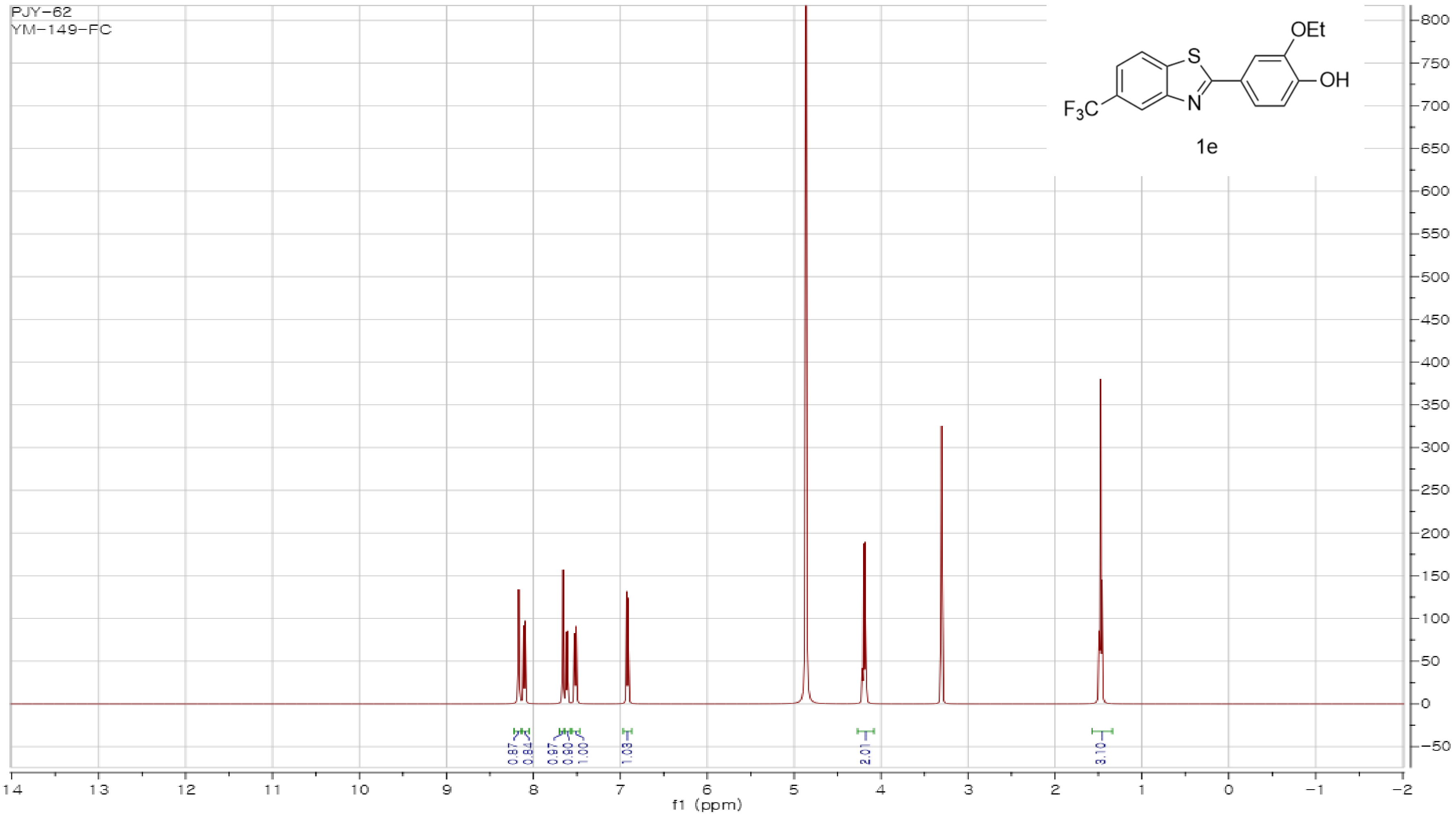

Figure S17. ^{1}H NMR spectrum of compound **1e.**

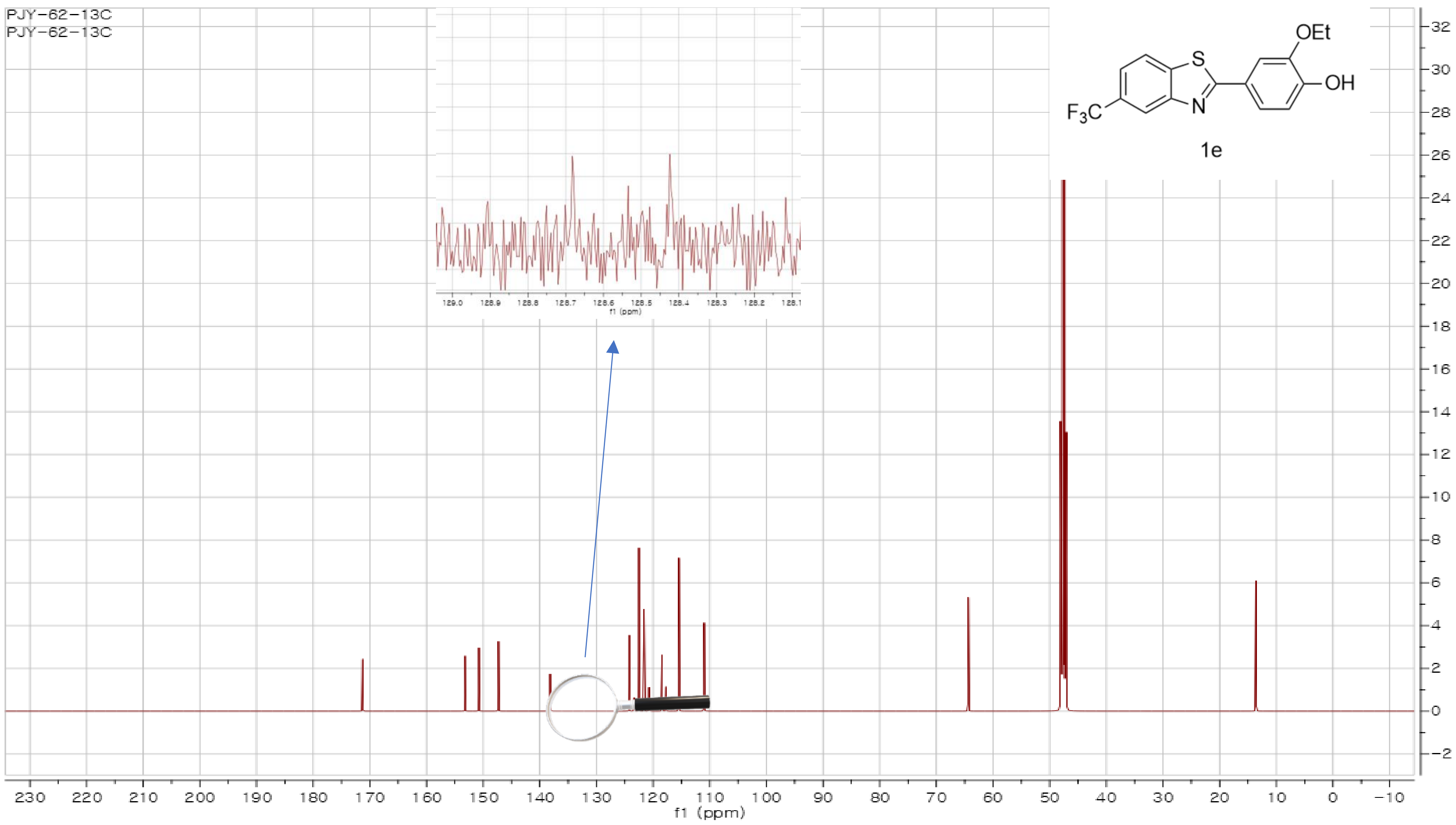

Figure S18. ^{13}C NMR spectrum of compound **1e.**

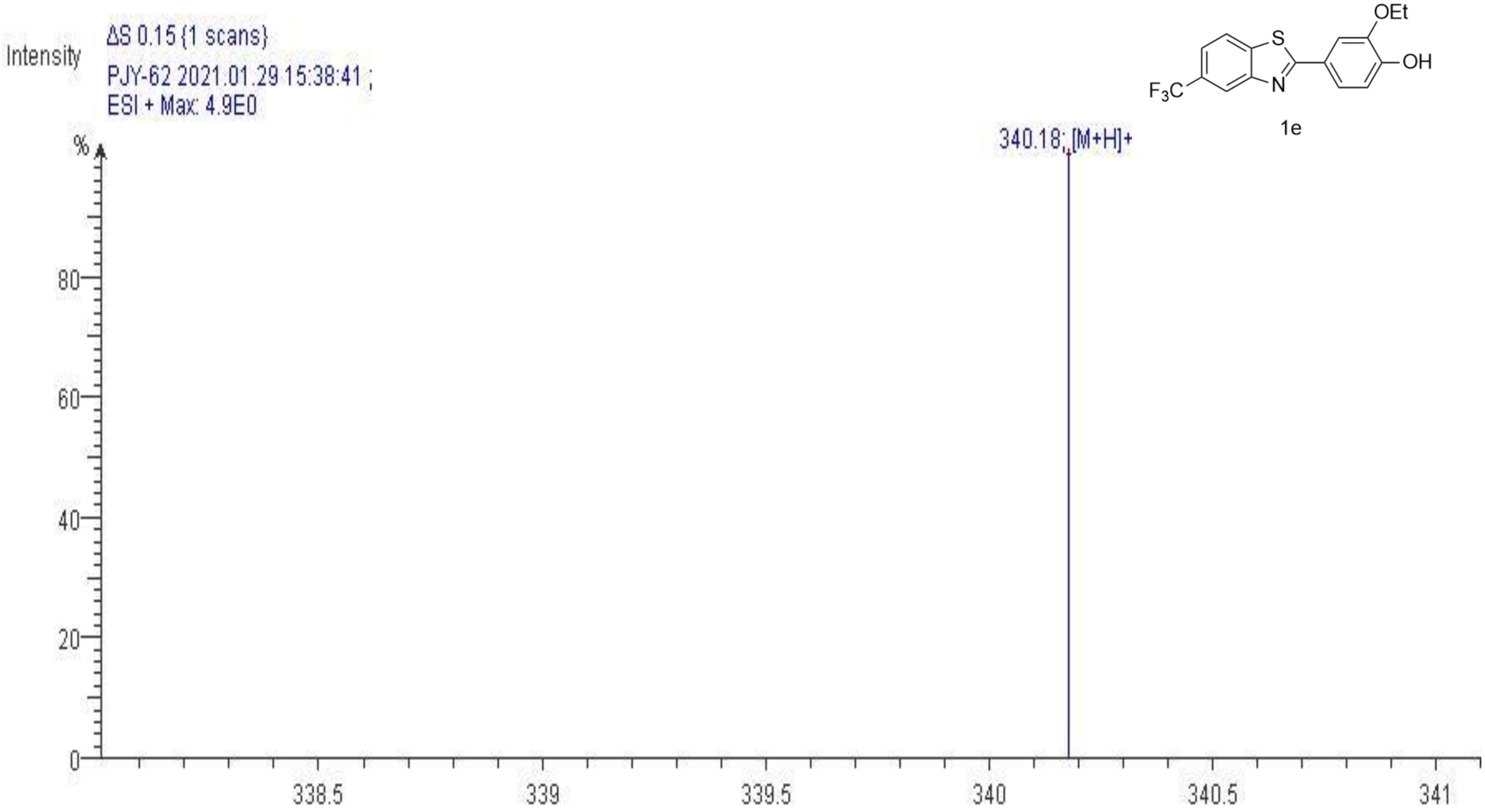

Figure S19. LRMS (ESI+) spectrum of compound **1e.**

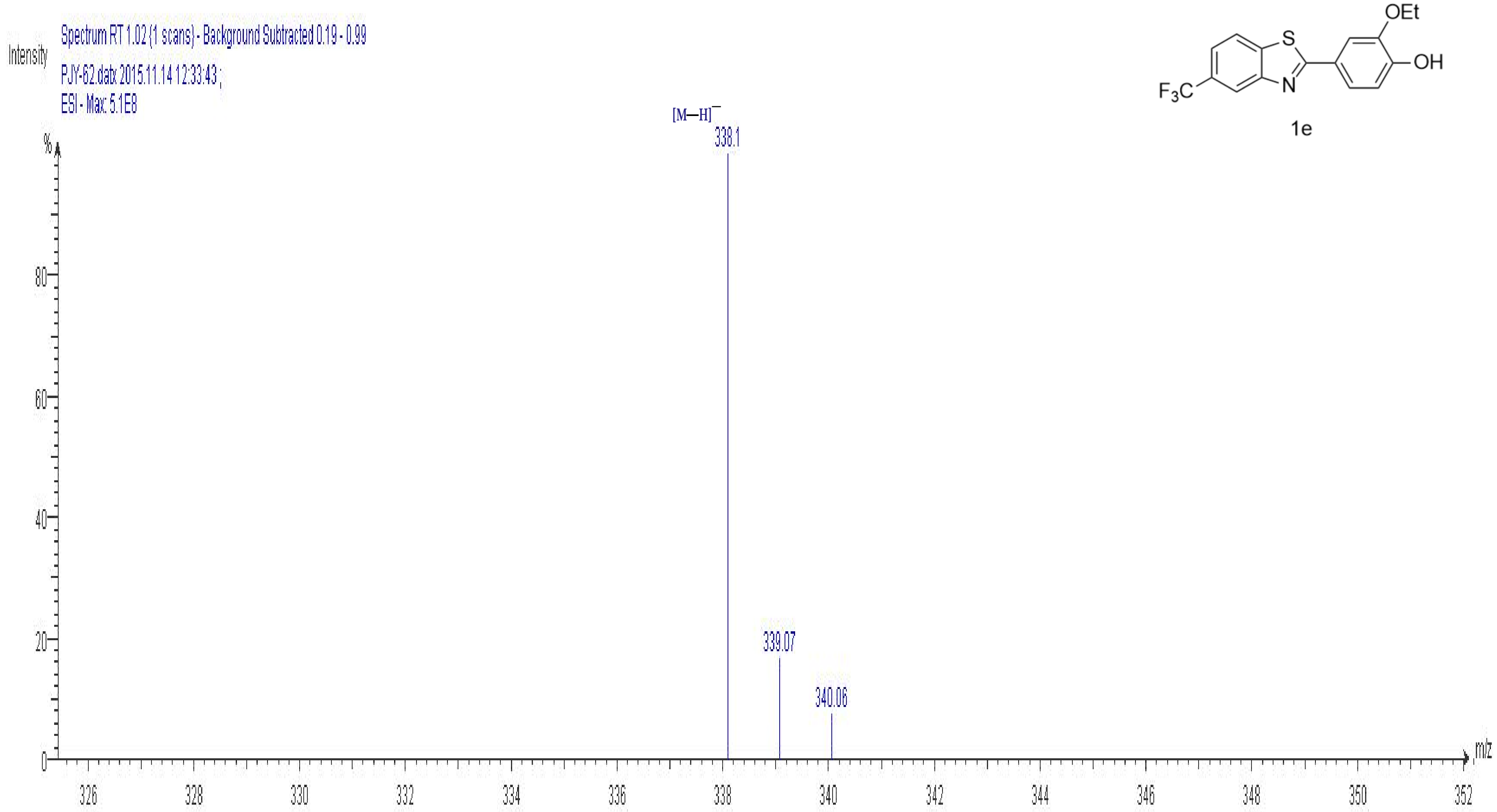

1e

Figure S20. LRMS (ESI–) spectrum of compound **1e.**

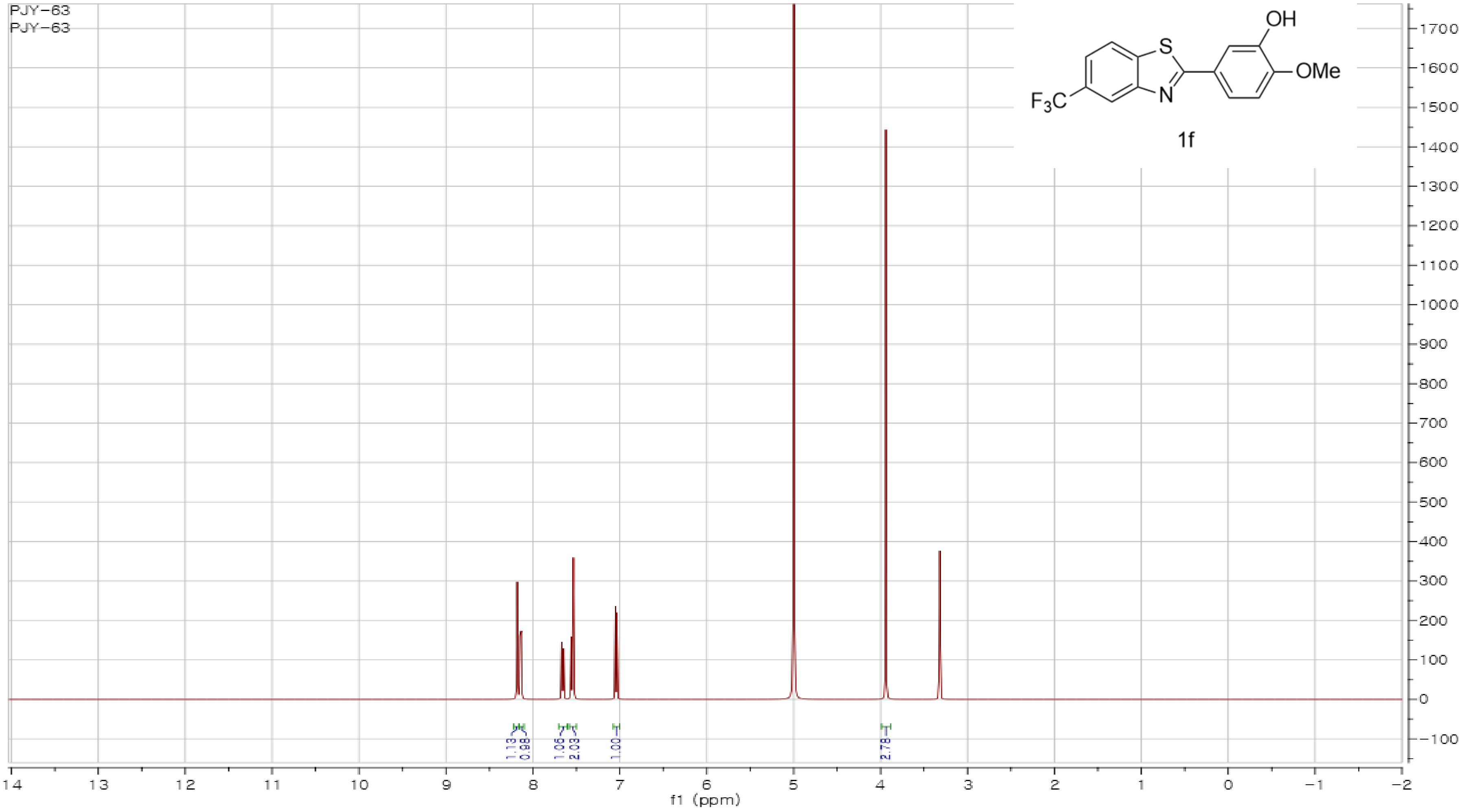

Figure S21. ^{1}H NMR spectrum of compound **1f.**

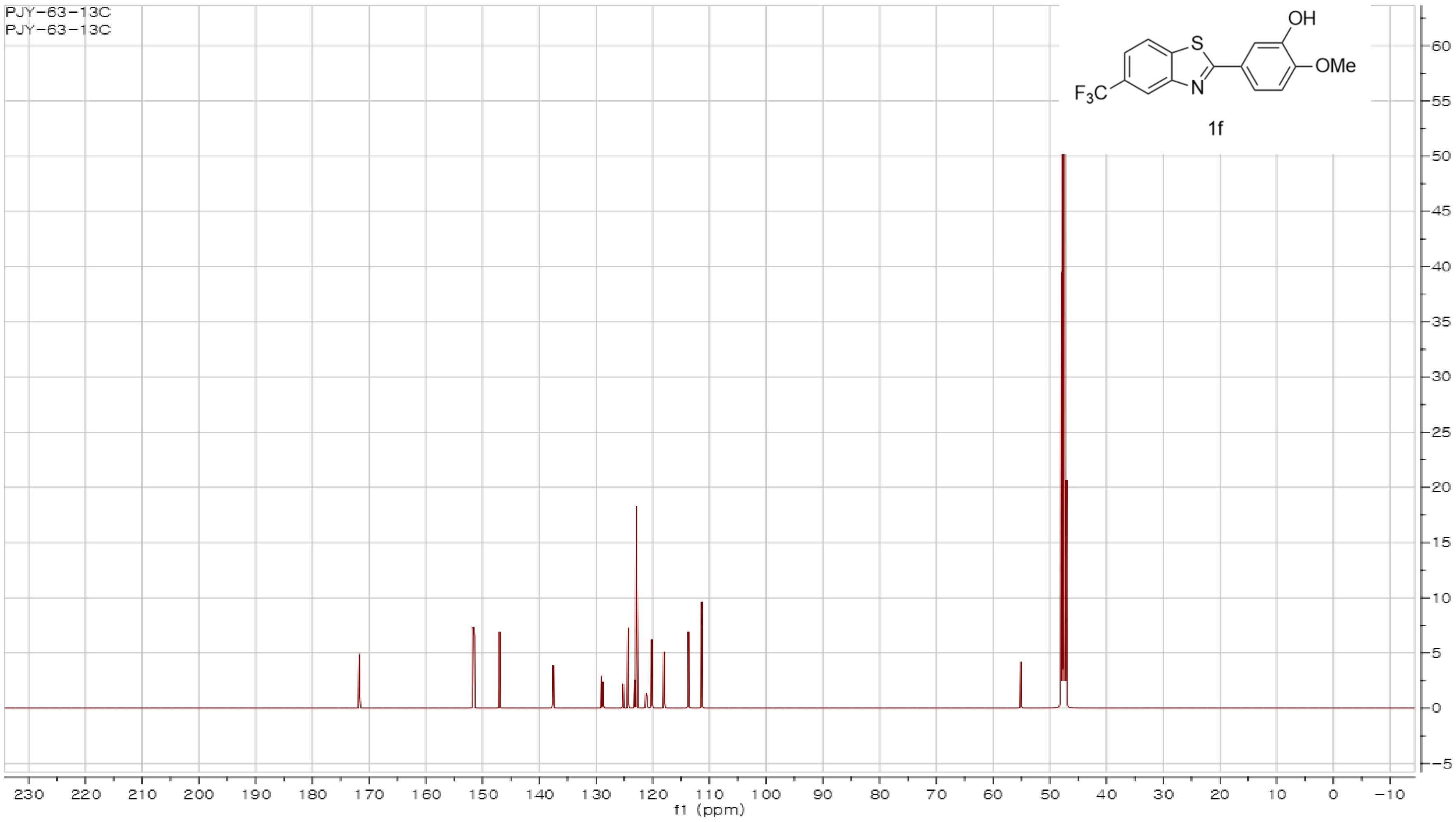

Figure S22. ^{13}C NMR spectrum of compound **1f.**

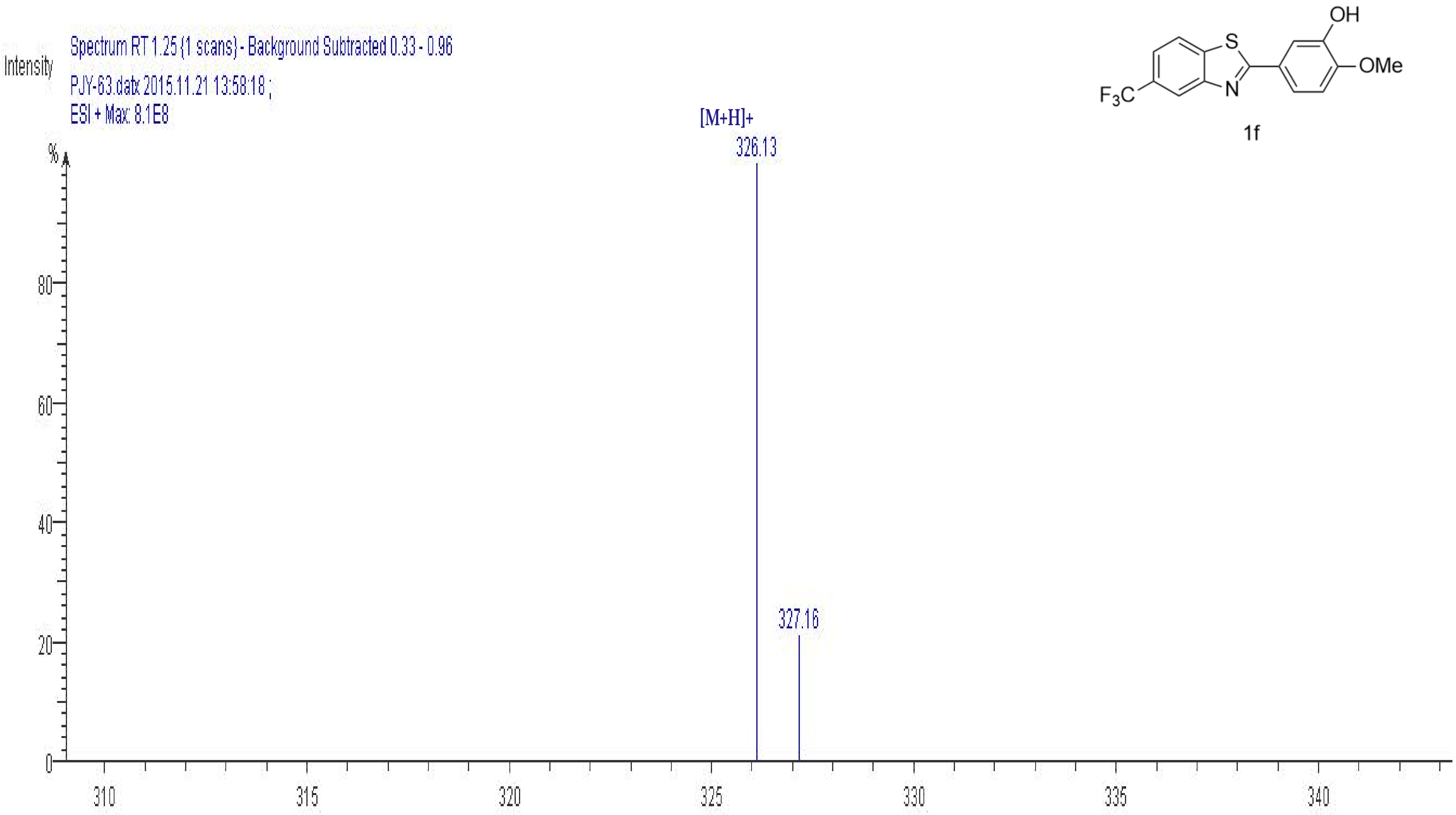

Figure S23. LRMS (ESI+) spectrum of compound **1f.**

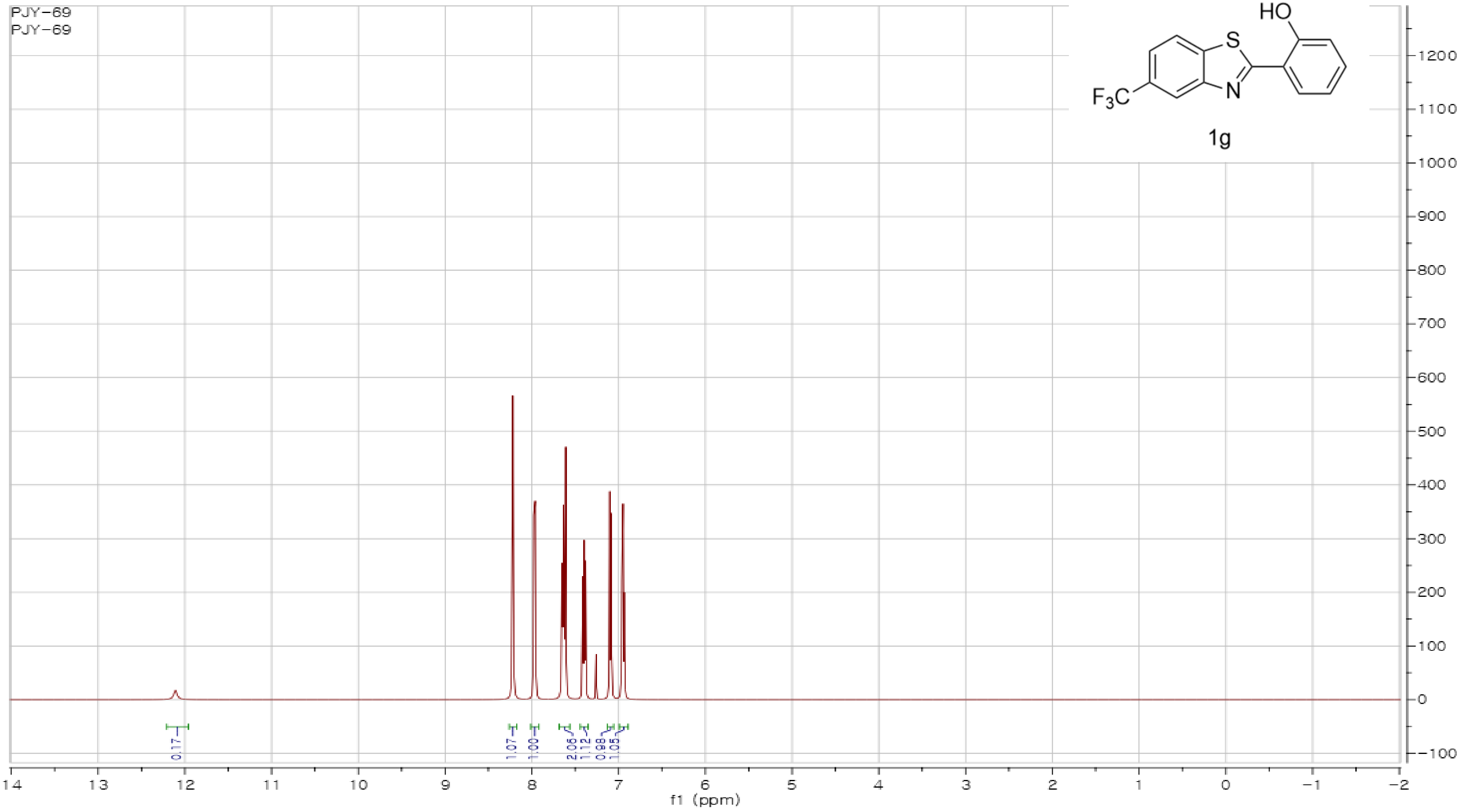

Figure S24. ^{1}H NMR spectrum of compound **1g.**

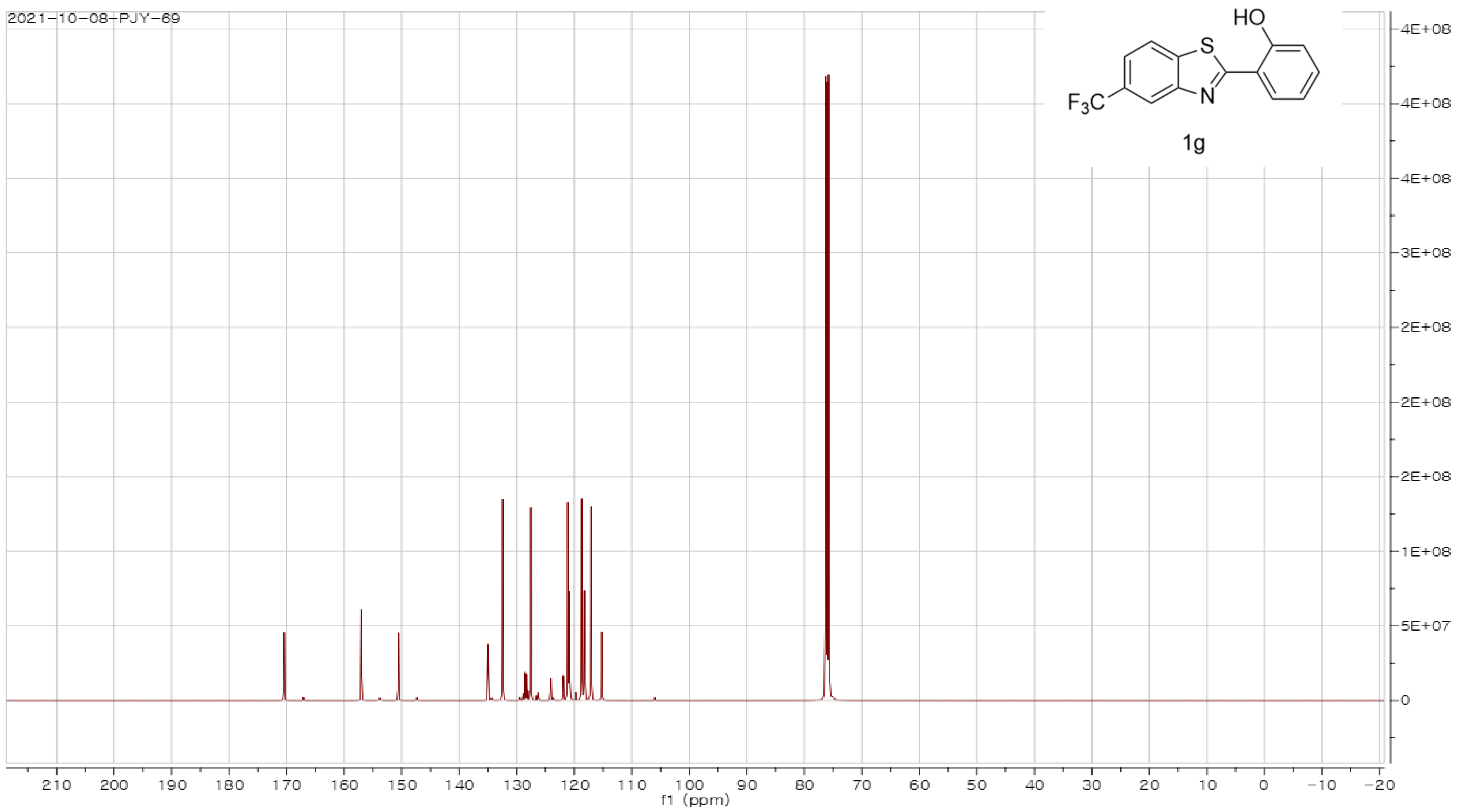

Figure S25. ^{13}C NMR spectrum of compound **1g.**

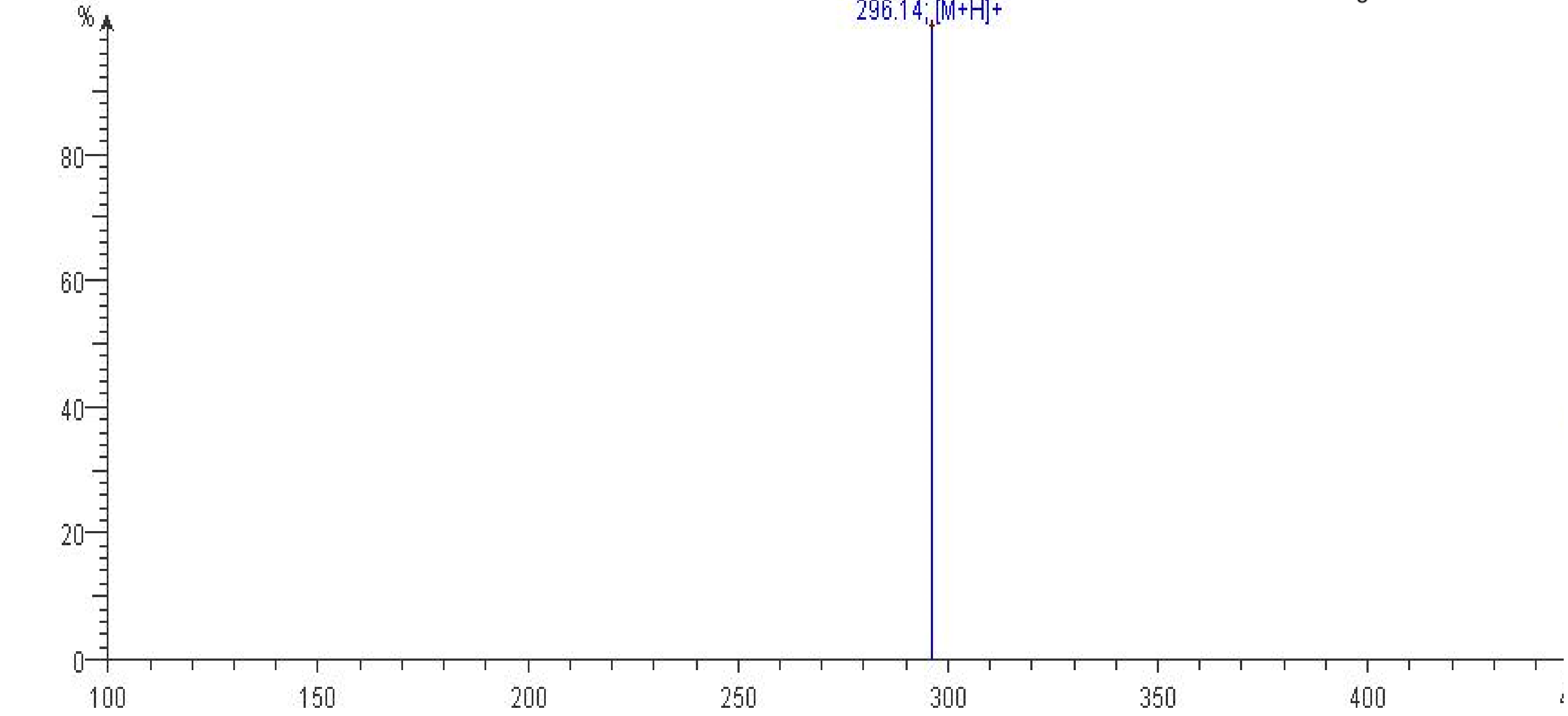

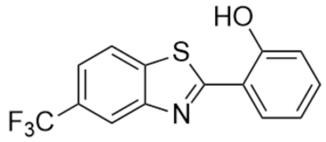

1g

Figure S26. LRMS (ESI+) spectrum of compound **1g.**

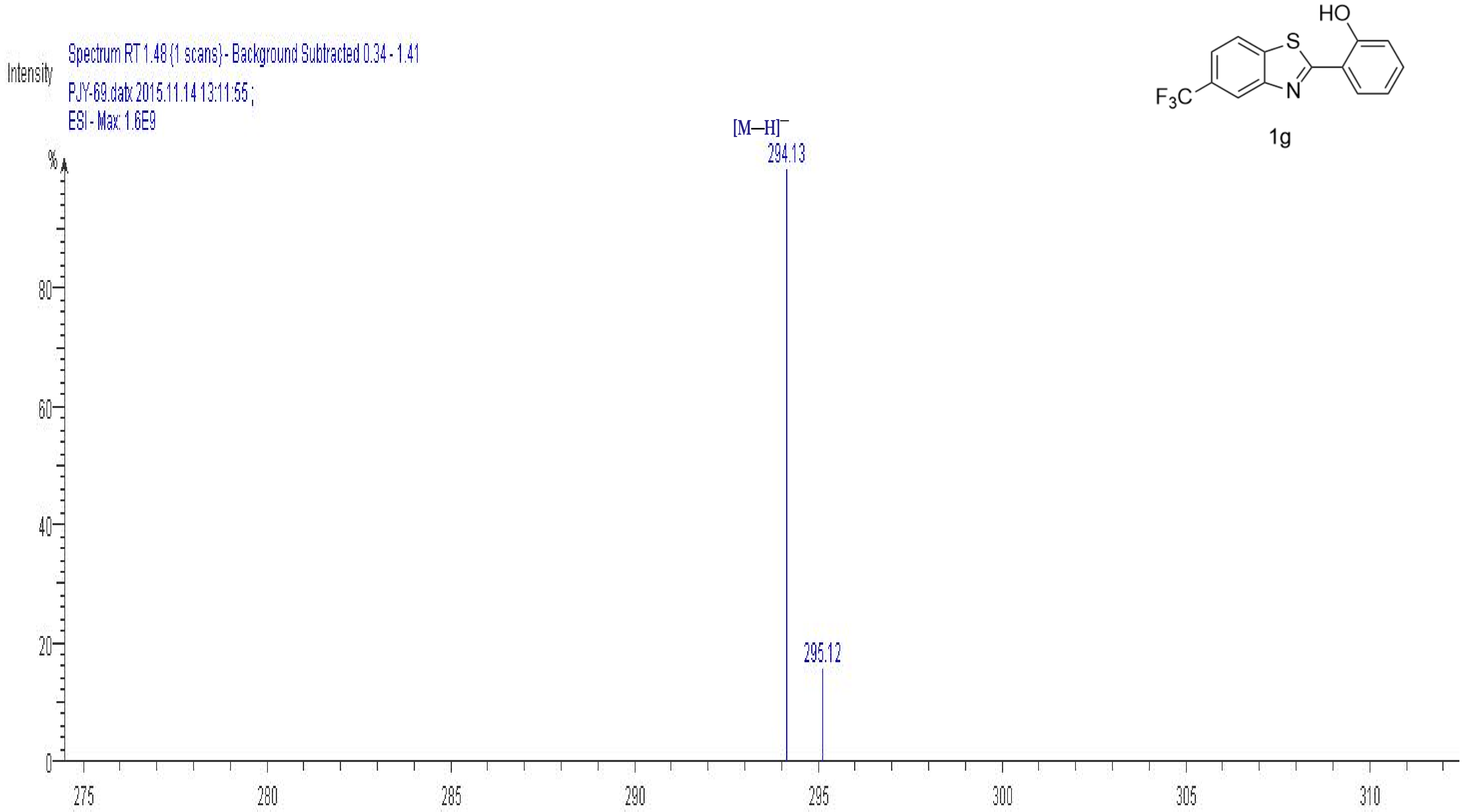

1g

Figure S27. LRMS (ESI–) spectrum of compound **1g.**

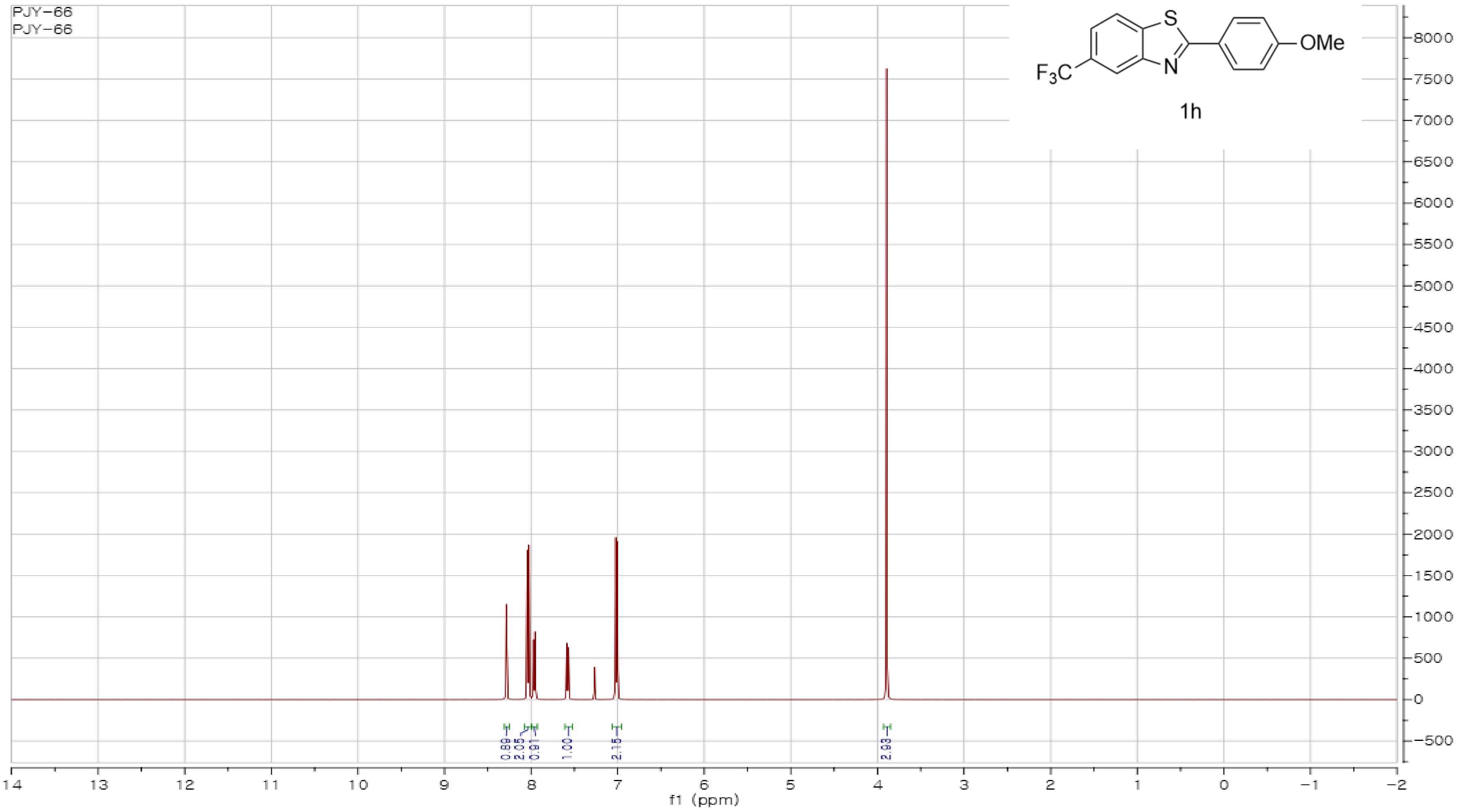

Figure S28. ^{1}H NMR spectrum of compound **1h.**

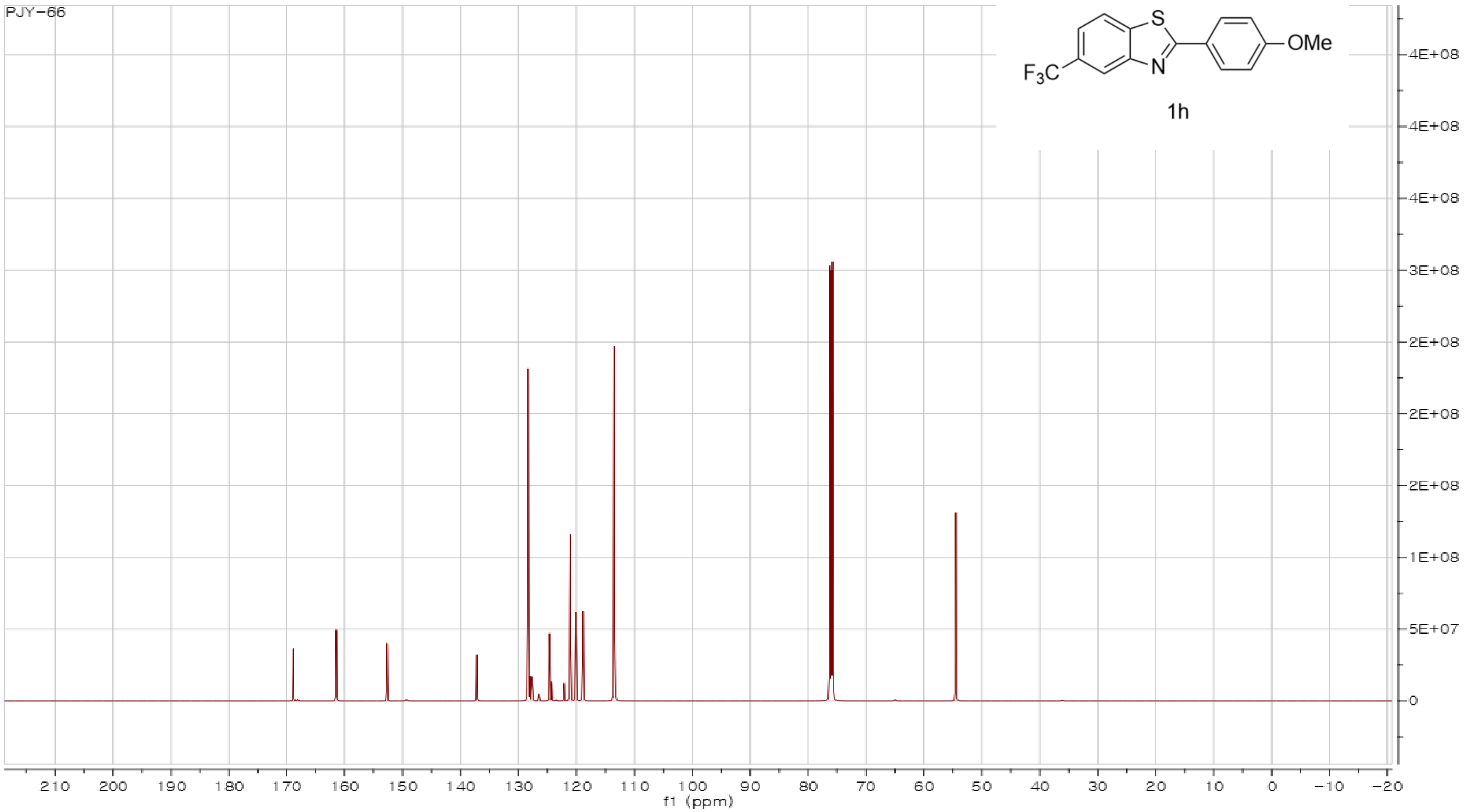

Figure S29. ^{13}C NMR spectrum of compound **1h.**

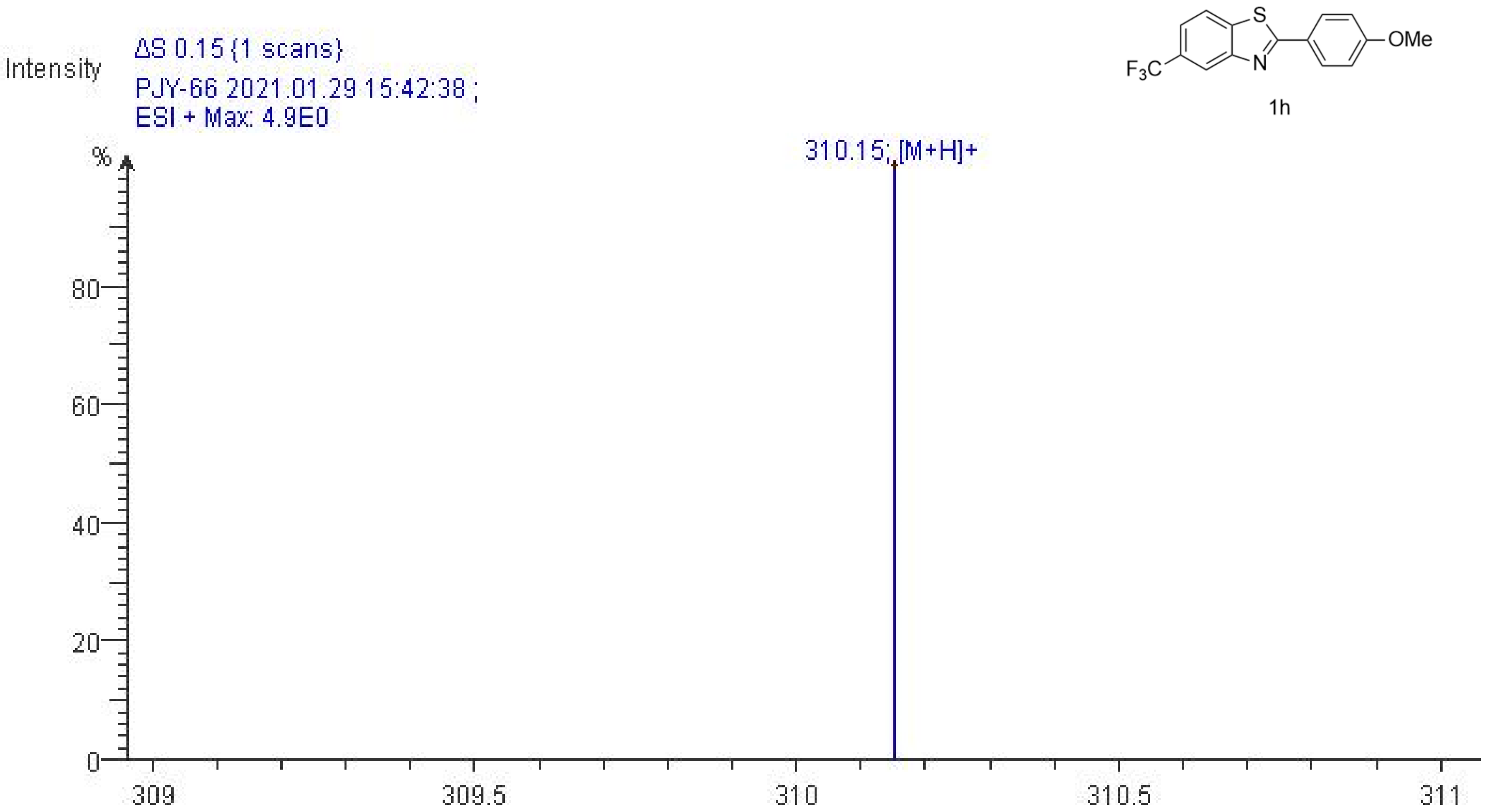

Figure S30. LRMS (ESI+) spectrum of compound **1h.**

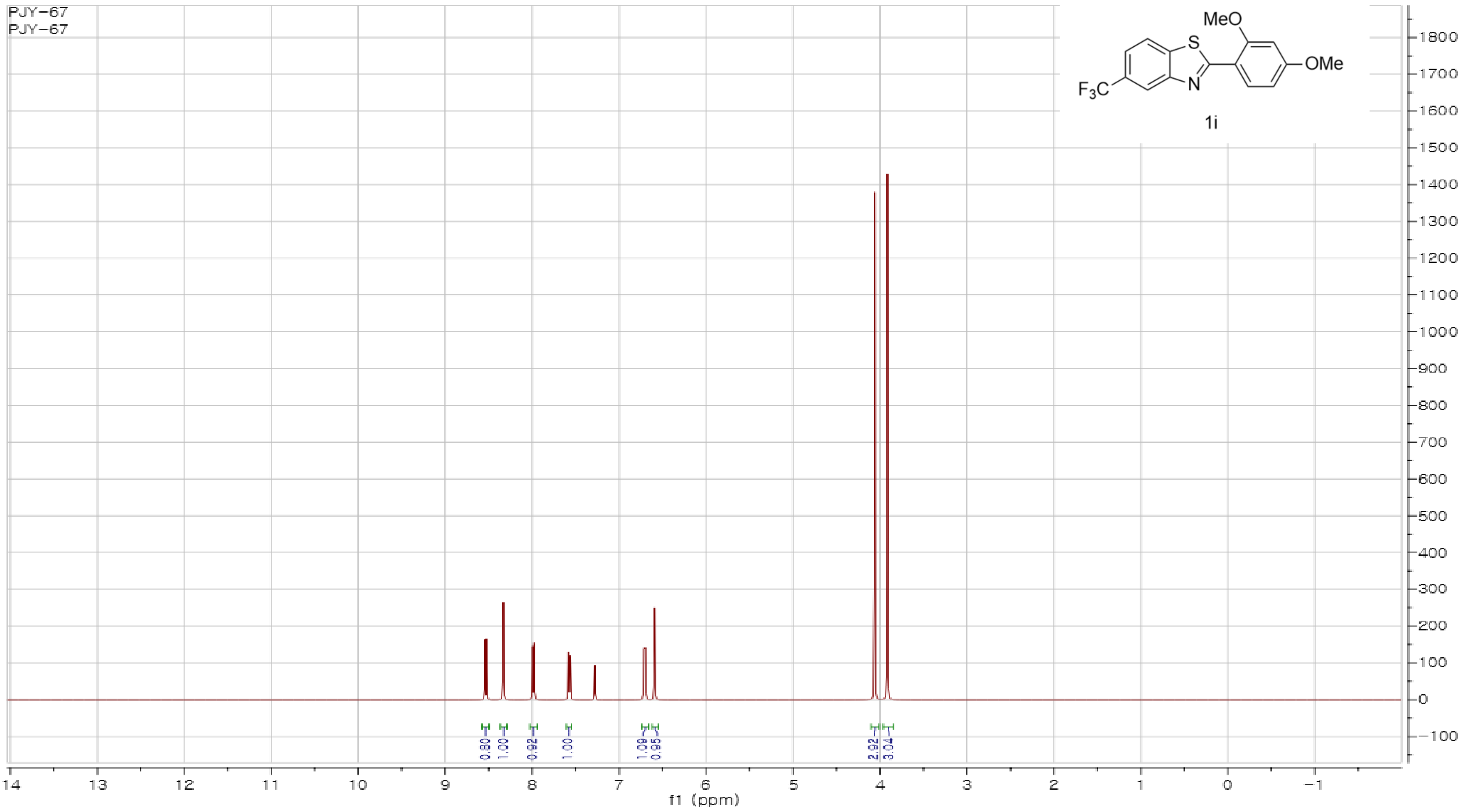

Figure S31. ^{1}H NMR spectrum of compound **1i.**

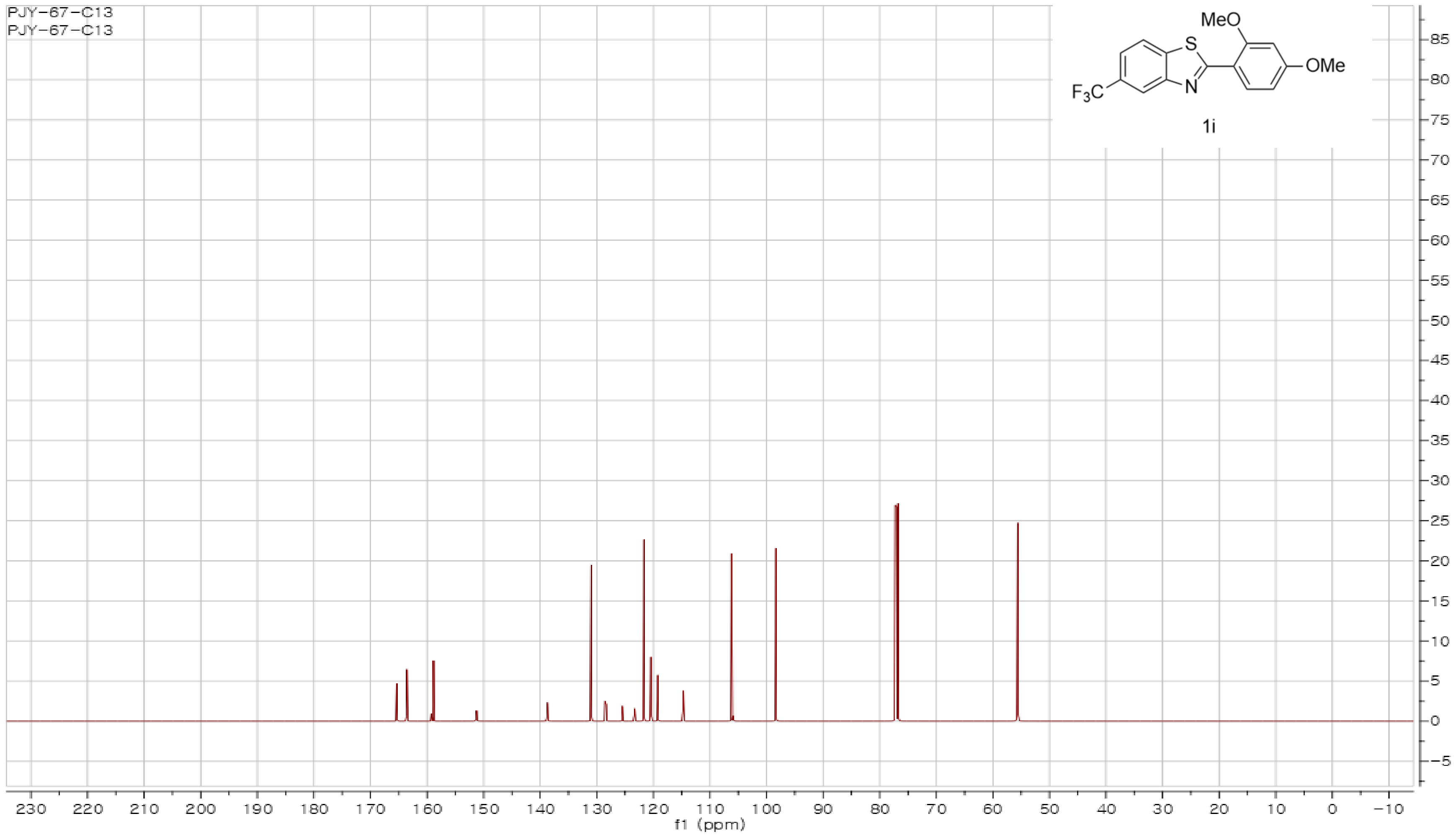

Figure S32. ^{13}C NMR spectrum of compound **1i.**

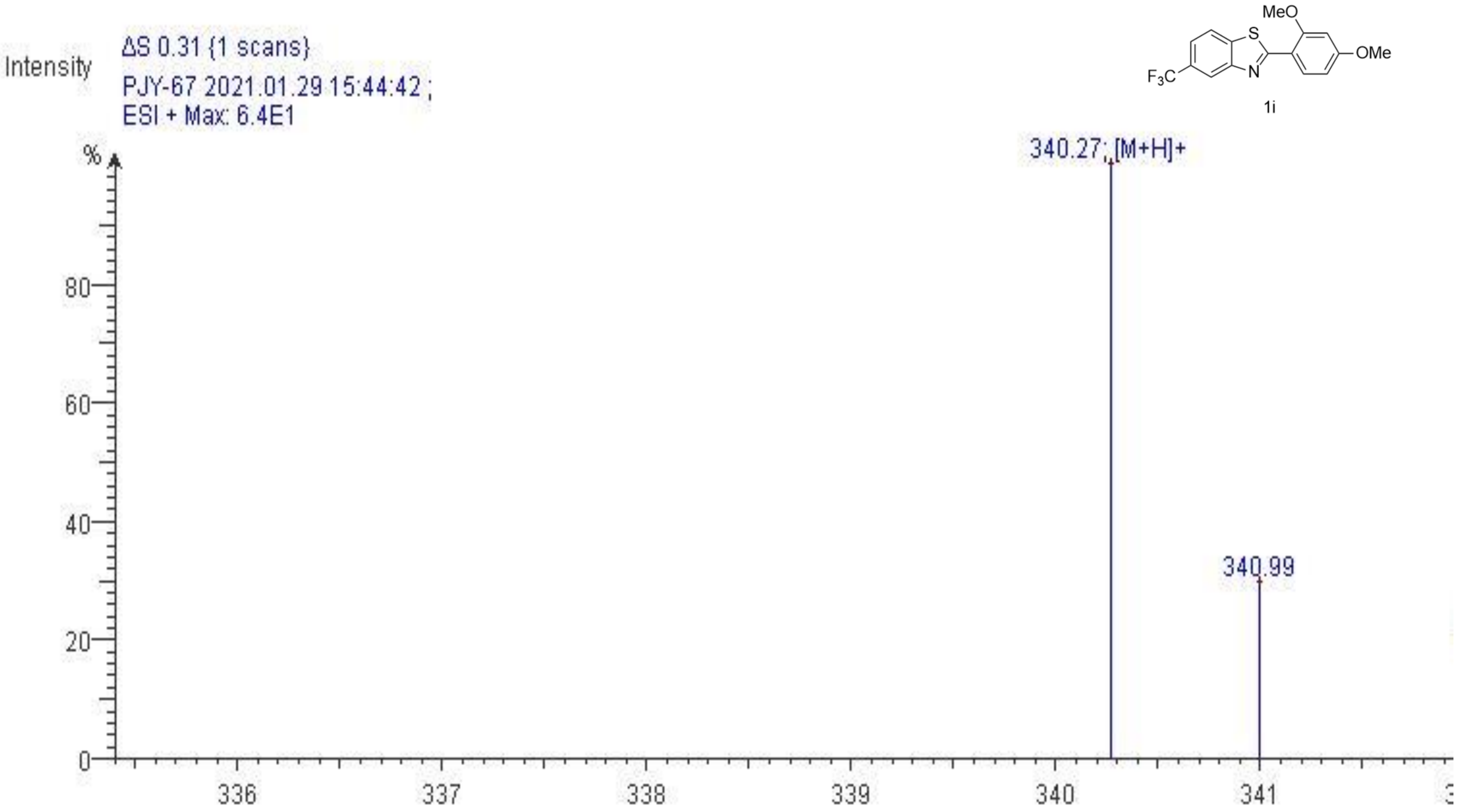

Figure S33. LRMS (ESI+) spectrum of compound **1i.**

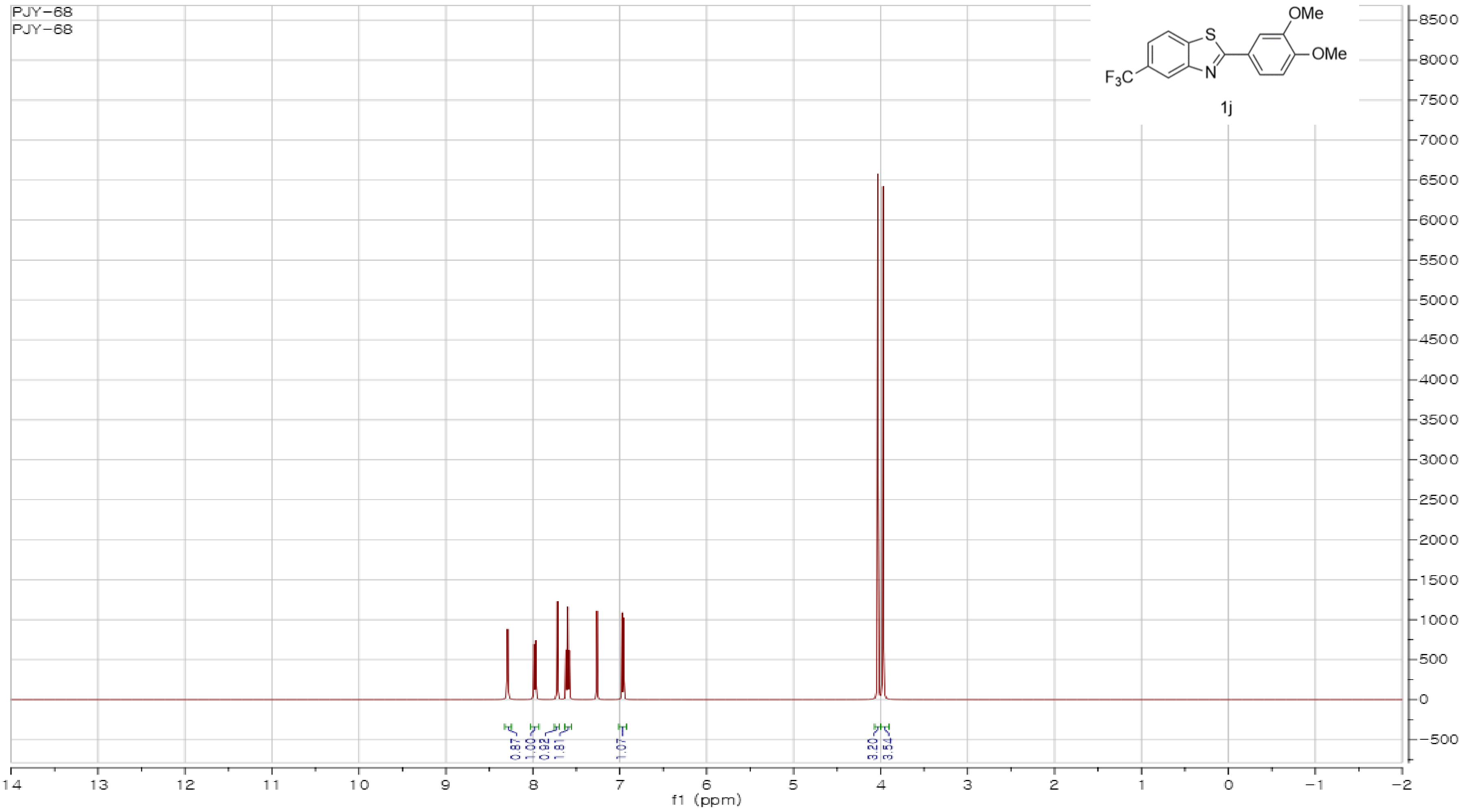

Figure S34. ^{1}H NMR spectrum of compound **1j.**

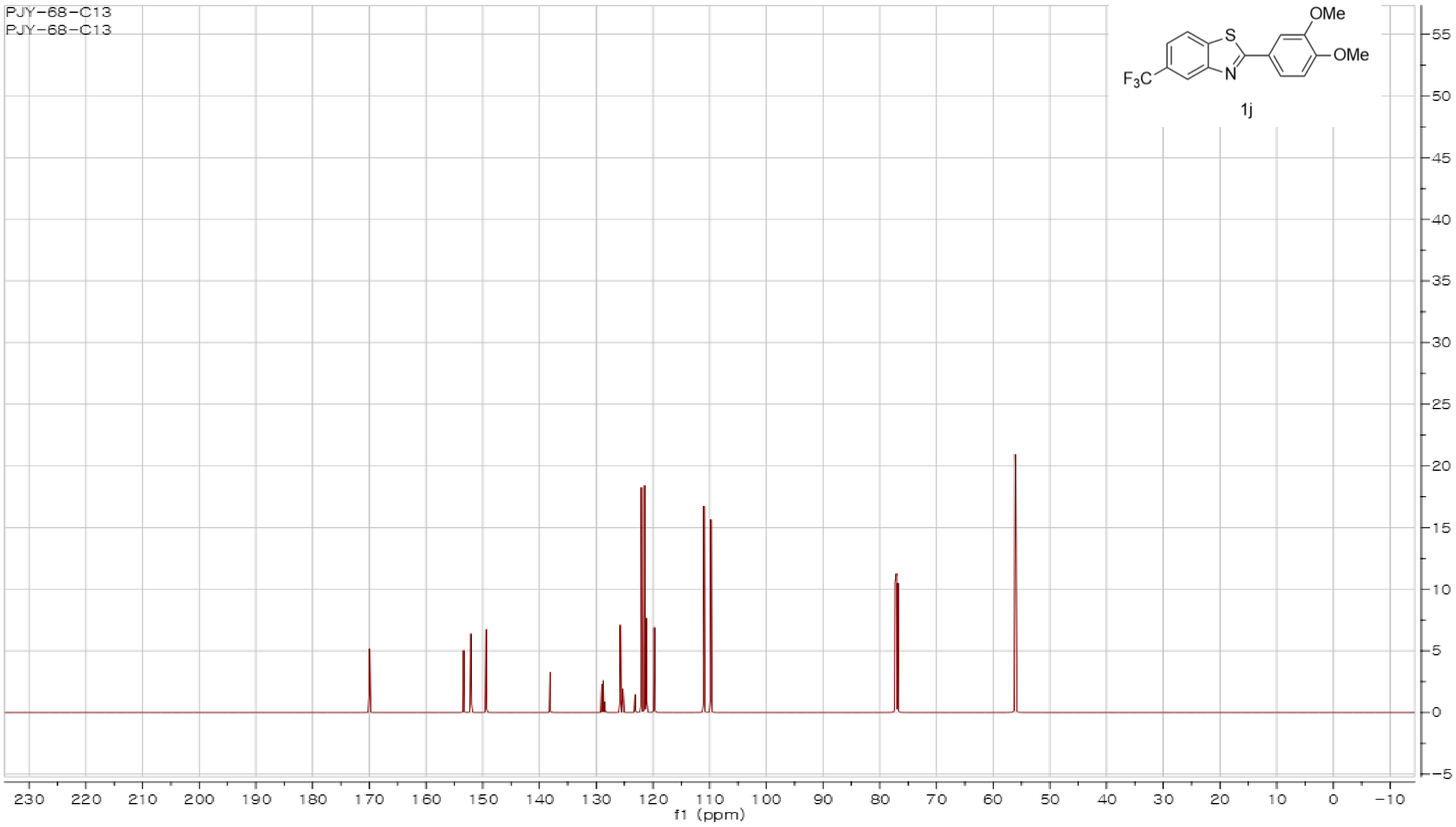

Figure S35. ^{13}C NMR spectrum of compound **1j.**

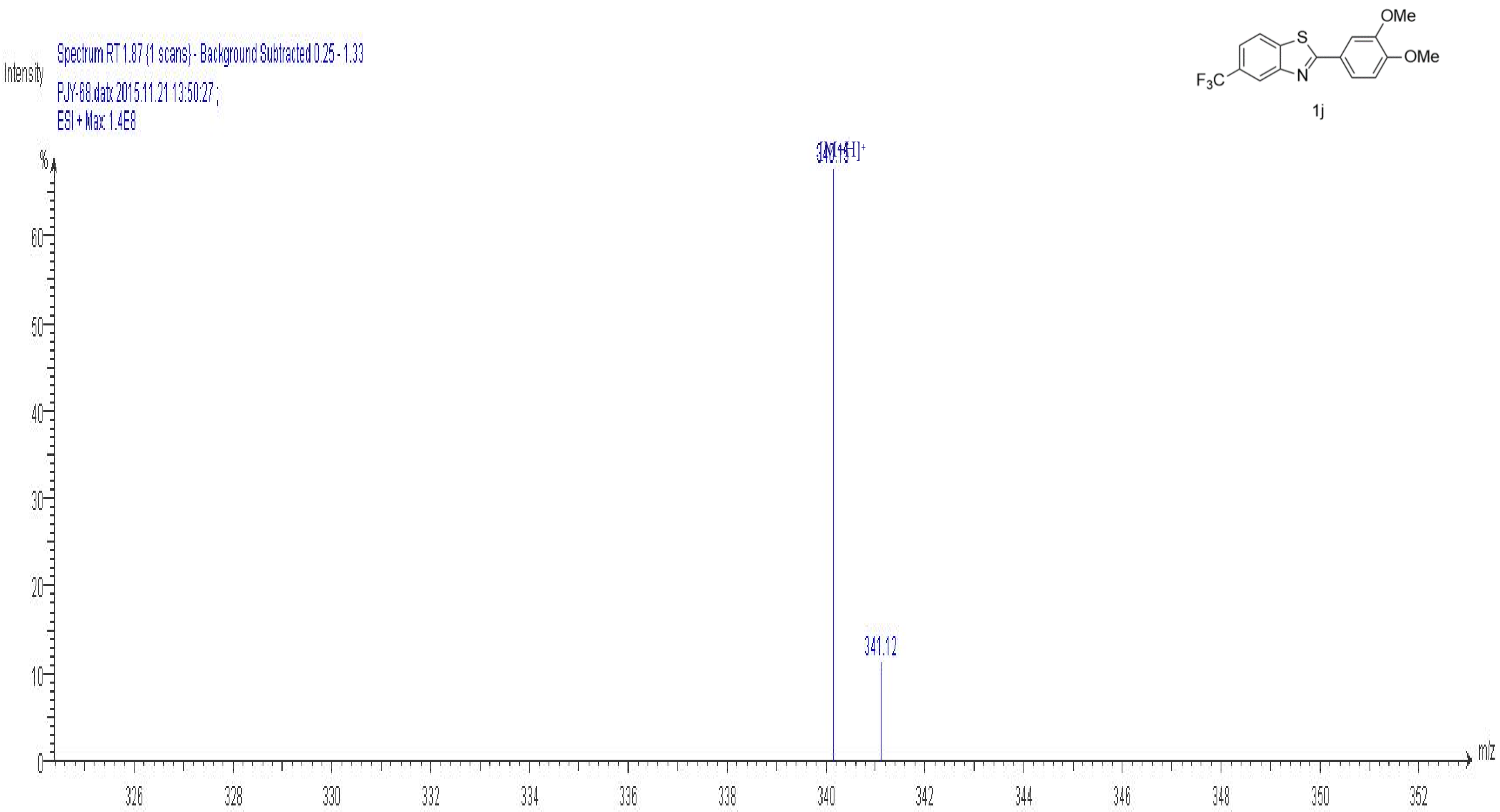

Figure S36. LRMS (ESI+) spectrum of compound **1j.**

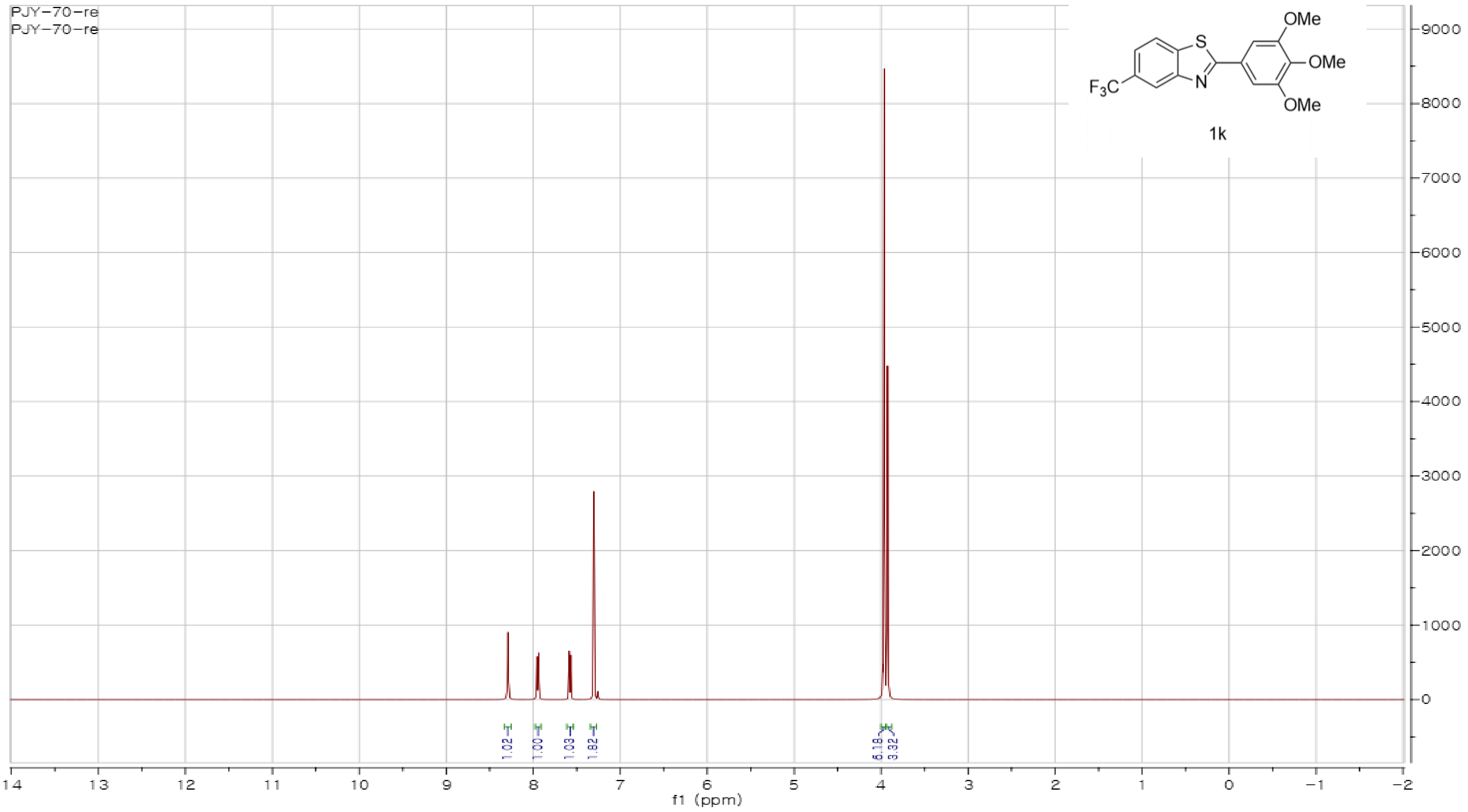

Figure S37. ^{1}H NMR spectrum of compound **1k.**

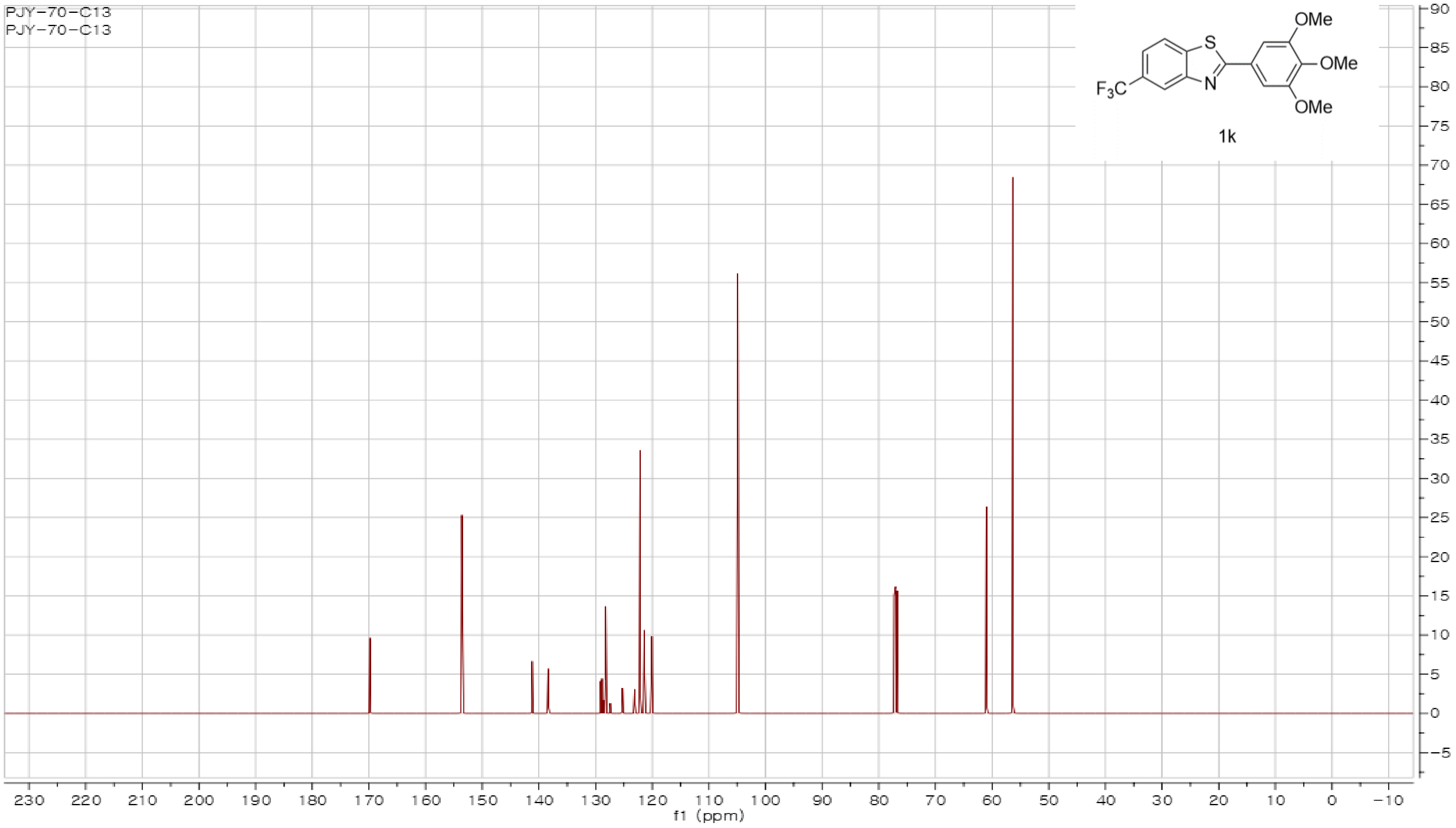

Figure S38. ^{13}C NMR spectrum of compound **1k.**

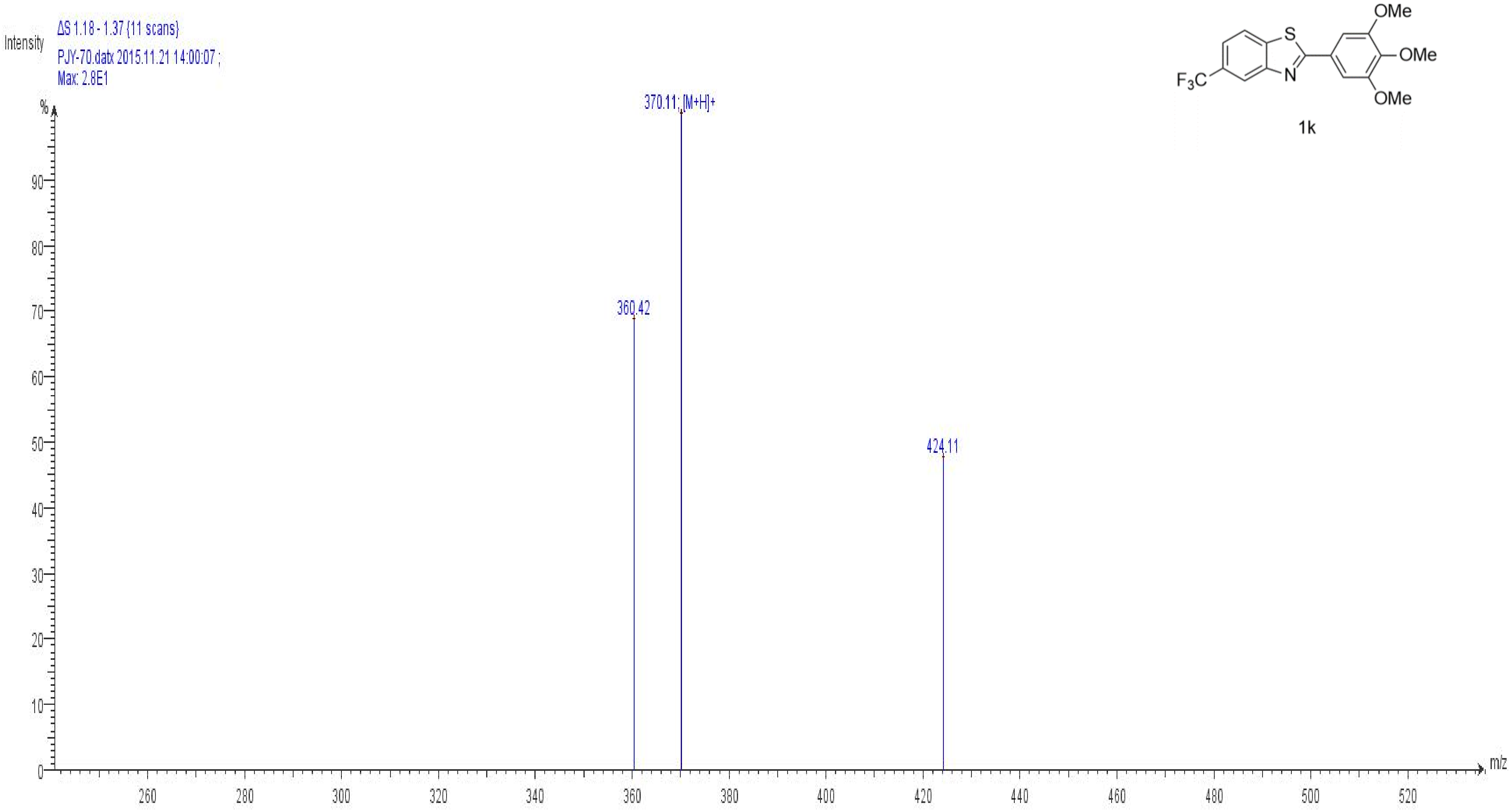

1k

Figure S39. LRMS (ESI+) spectrum of compound **1k.**

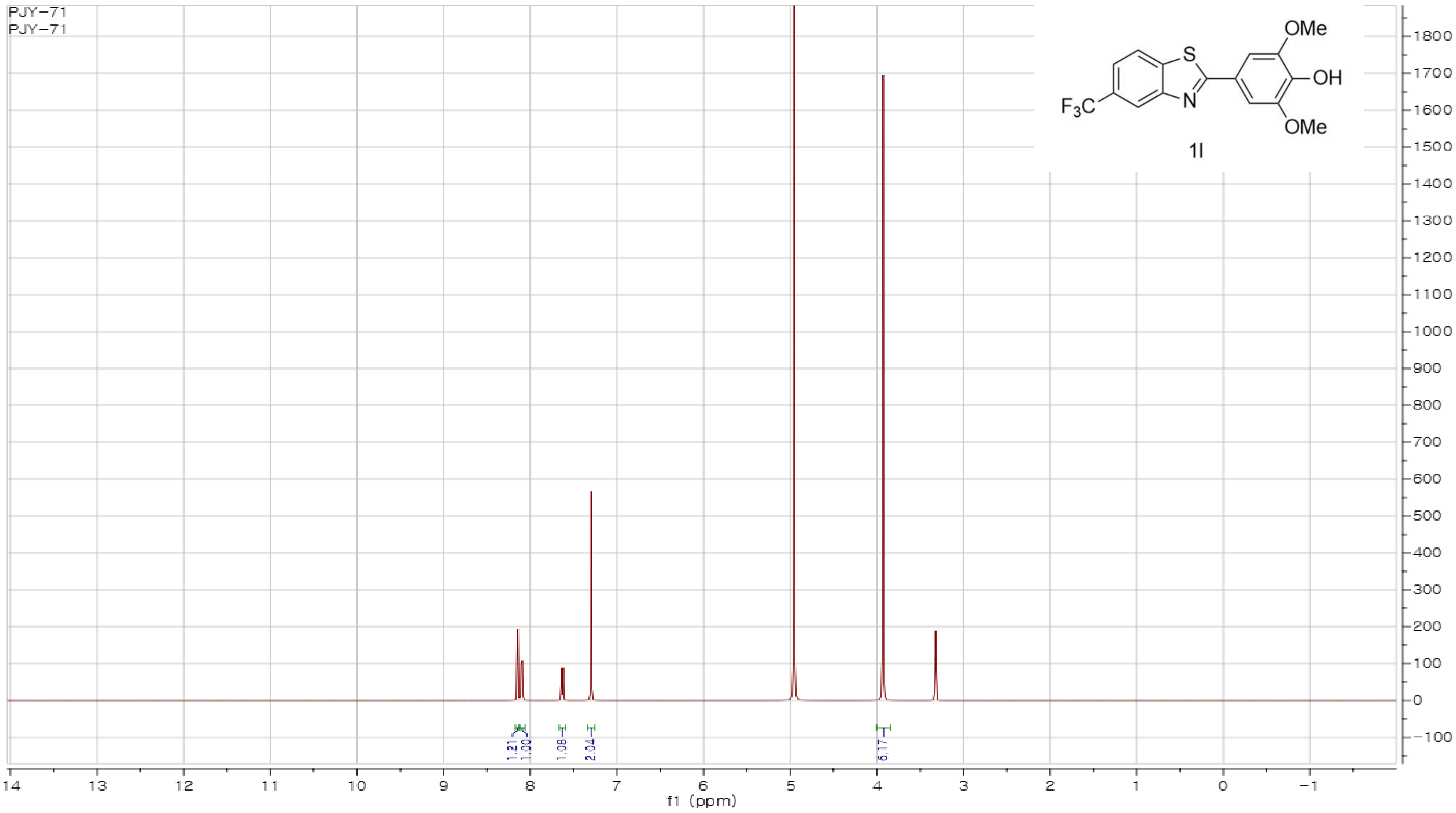

Figure S40. ^{1}H NMR spectrum of compound **1l.**

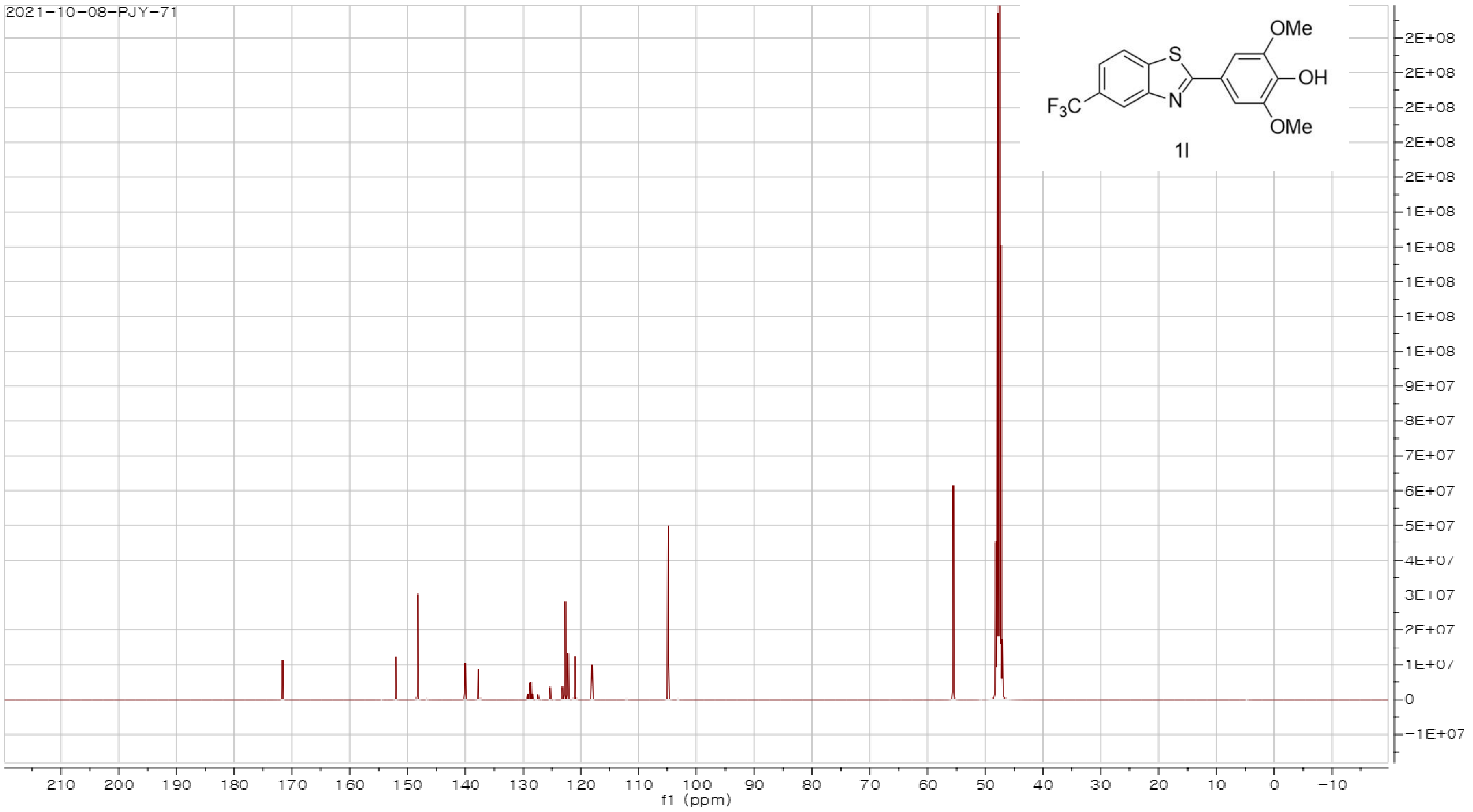

Figure S41. ^{13}C NMR spectrum of compound **1l.**

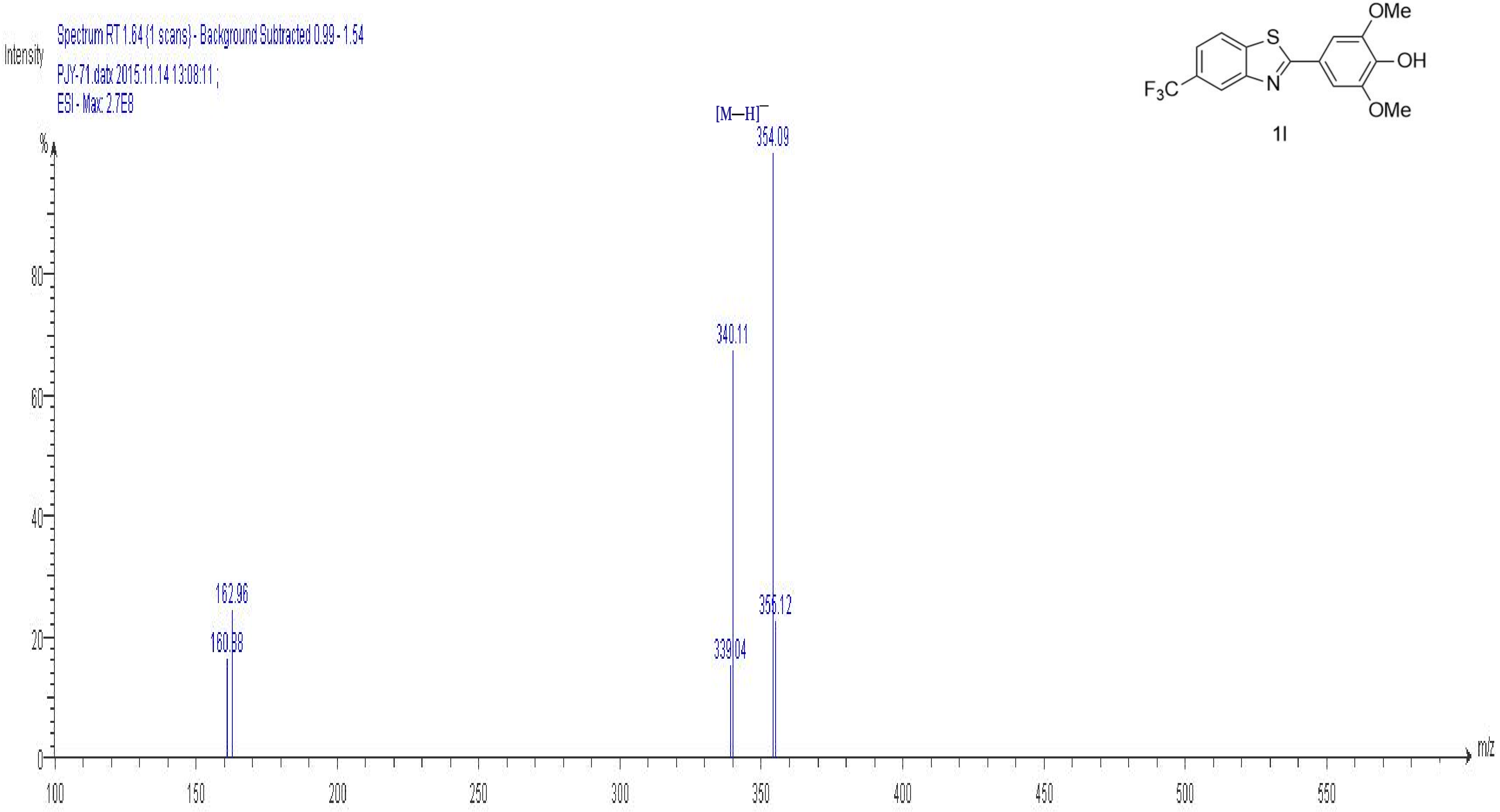

Figure S42. LRMS (ESI–) spectrum of compound **1l.**

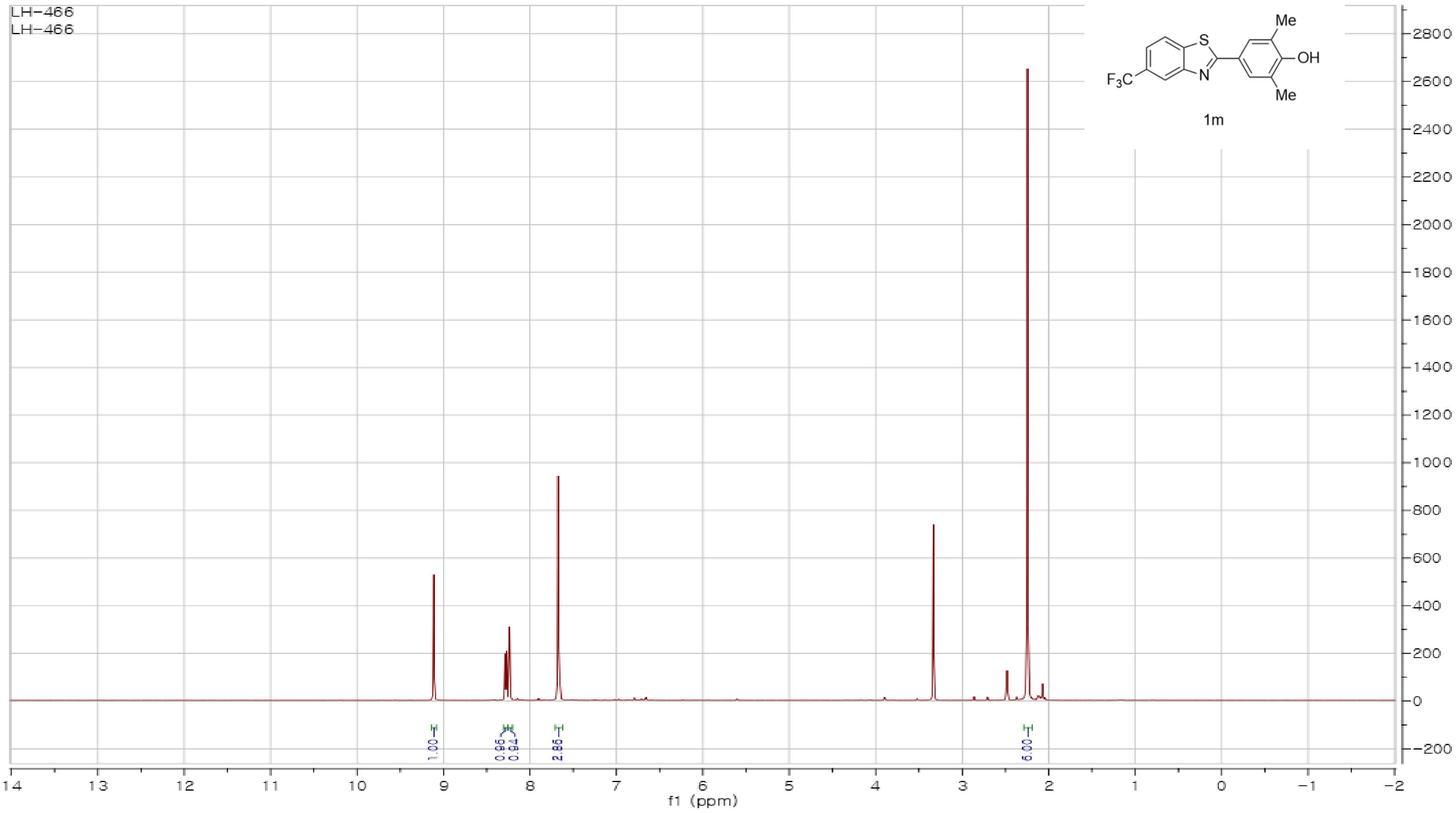

Figure S43. ^{1}H NMR spectrum of compound **1m.**

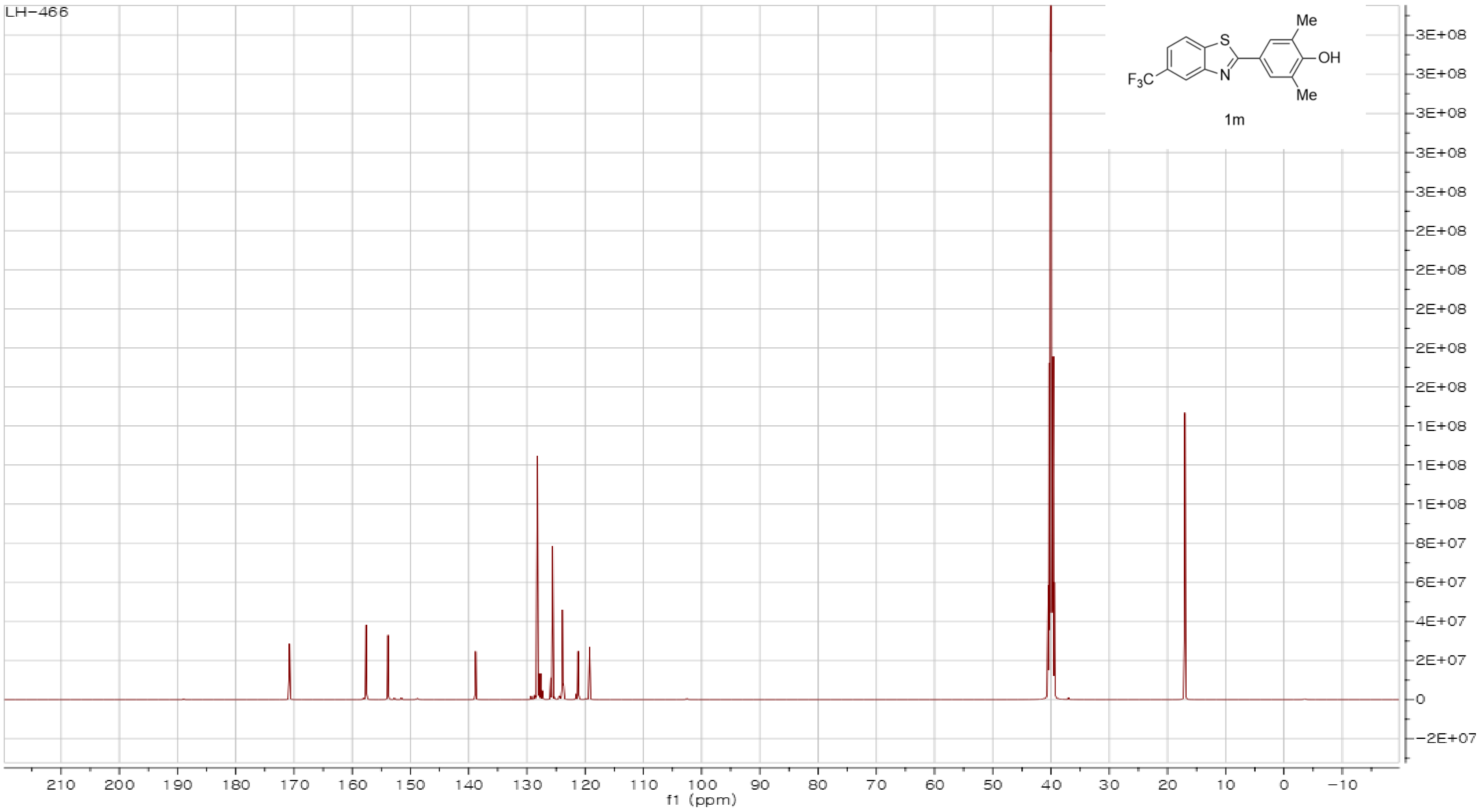

Figure S44. ^{13}C NMR spectrum of compound **1m.**

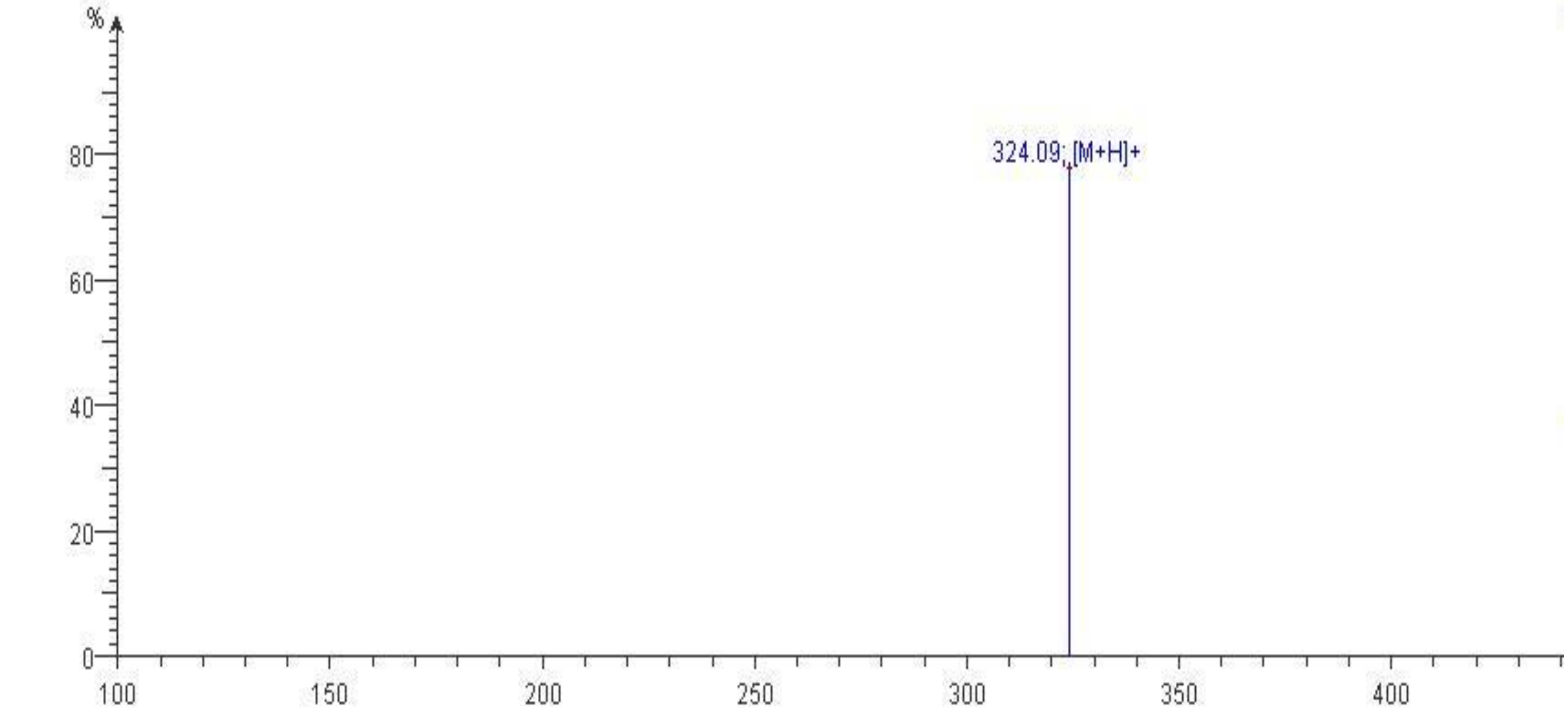

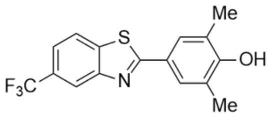

1m

Figure S45. LRMS (ESI+) spectrum of compound **1m.**

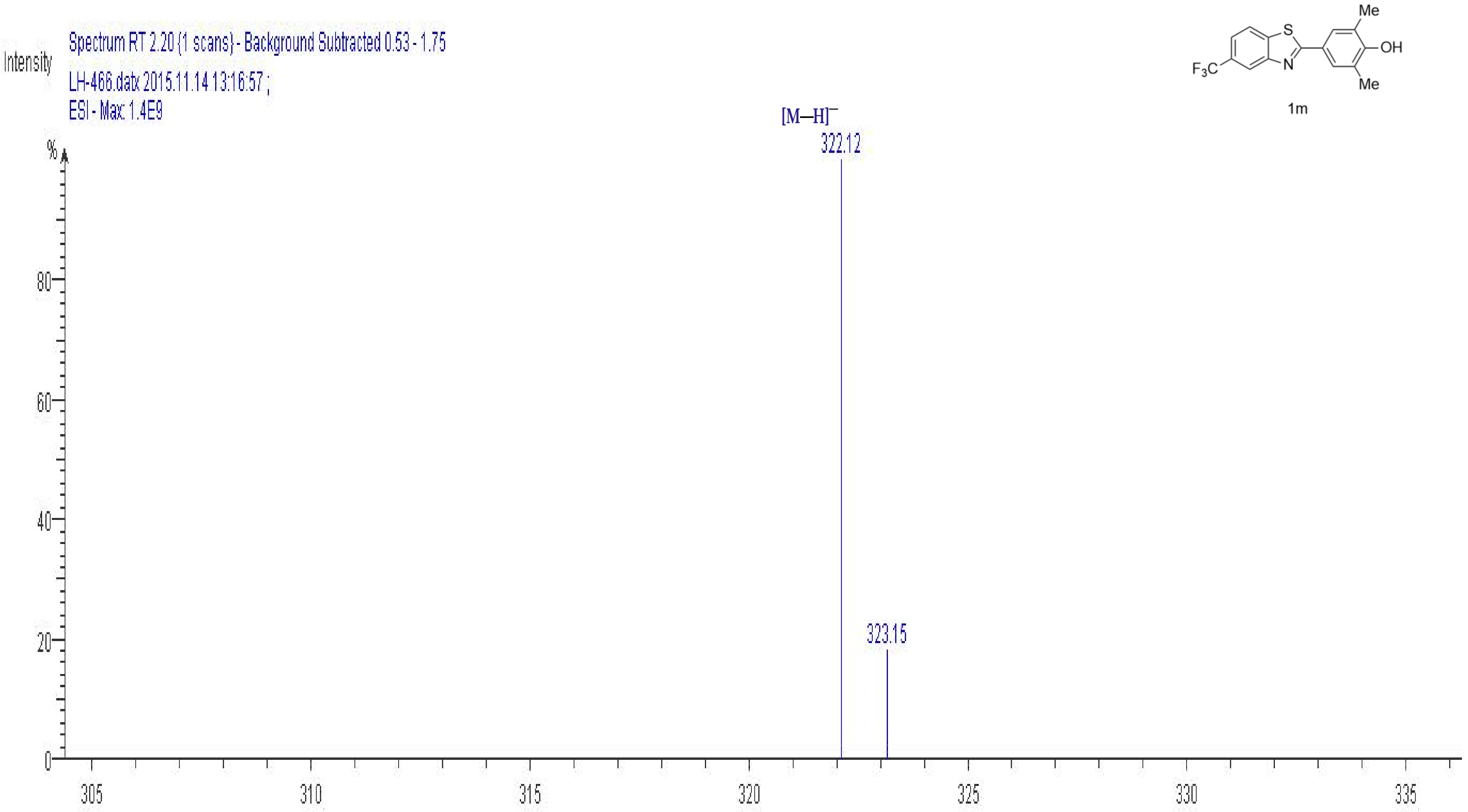

Figure S46. LRMS (ESI–) spectrum of compound **1m.**

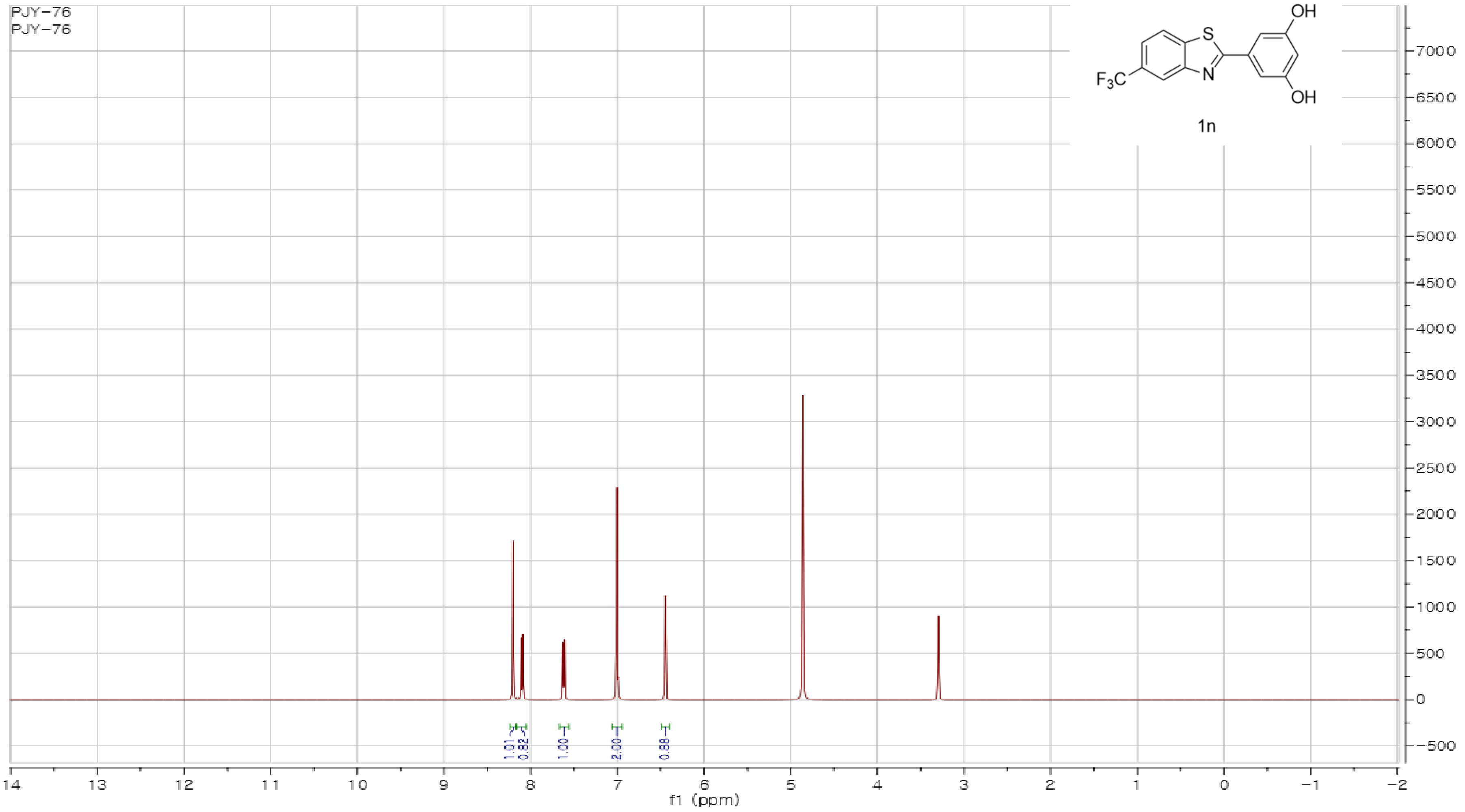

Figure S47. ^{1}H NMR spectrum of compound **1n.**

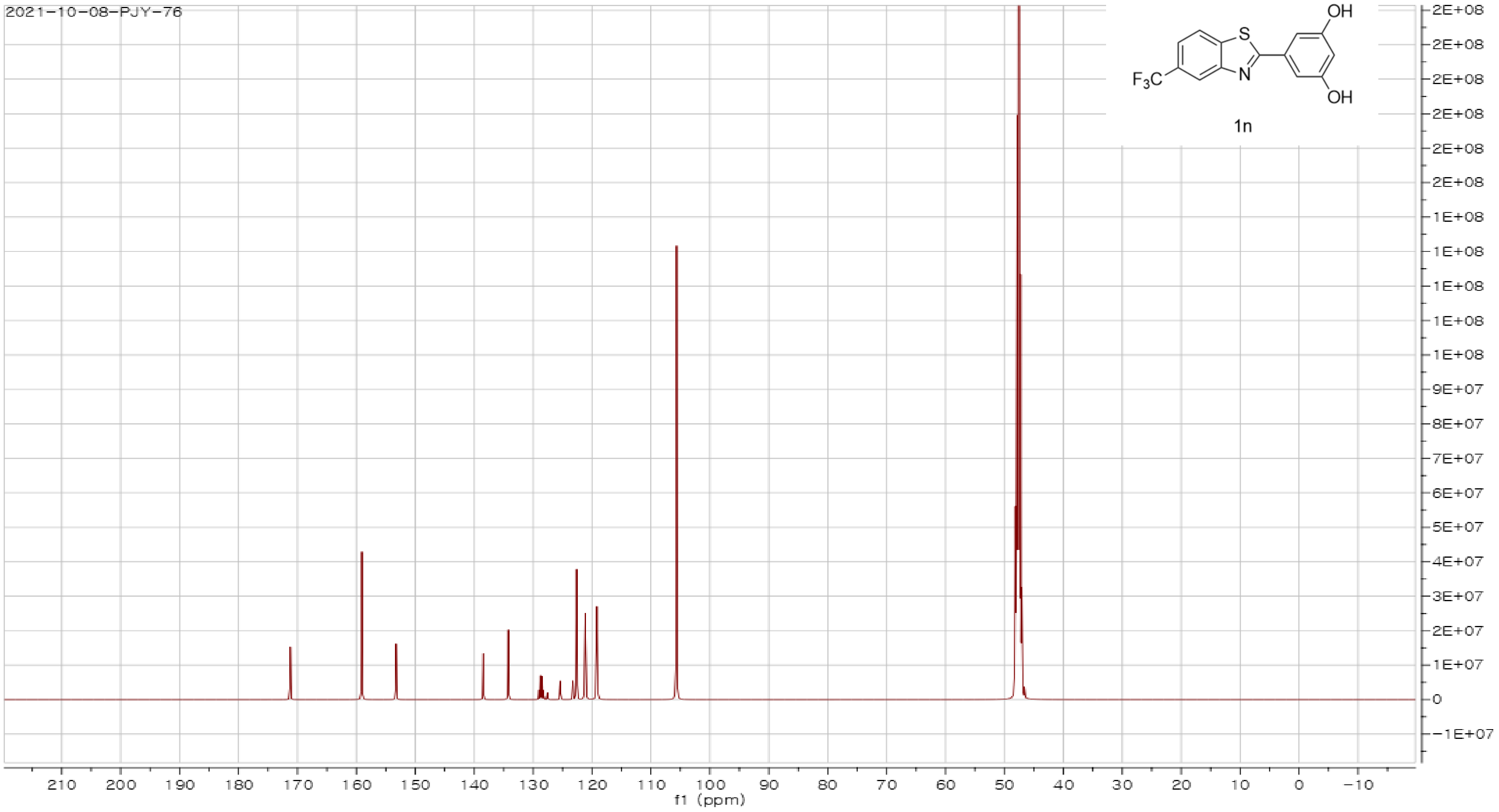

Figure S48. ^{13}C NMR spectrum of compound **1n.**

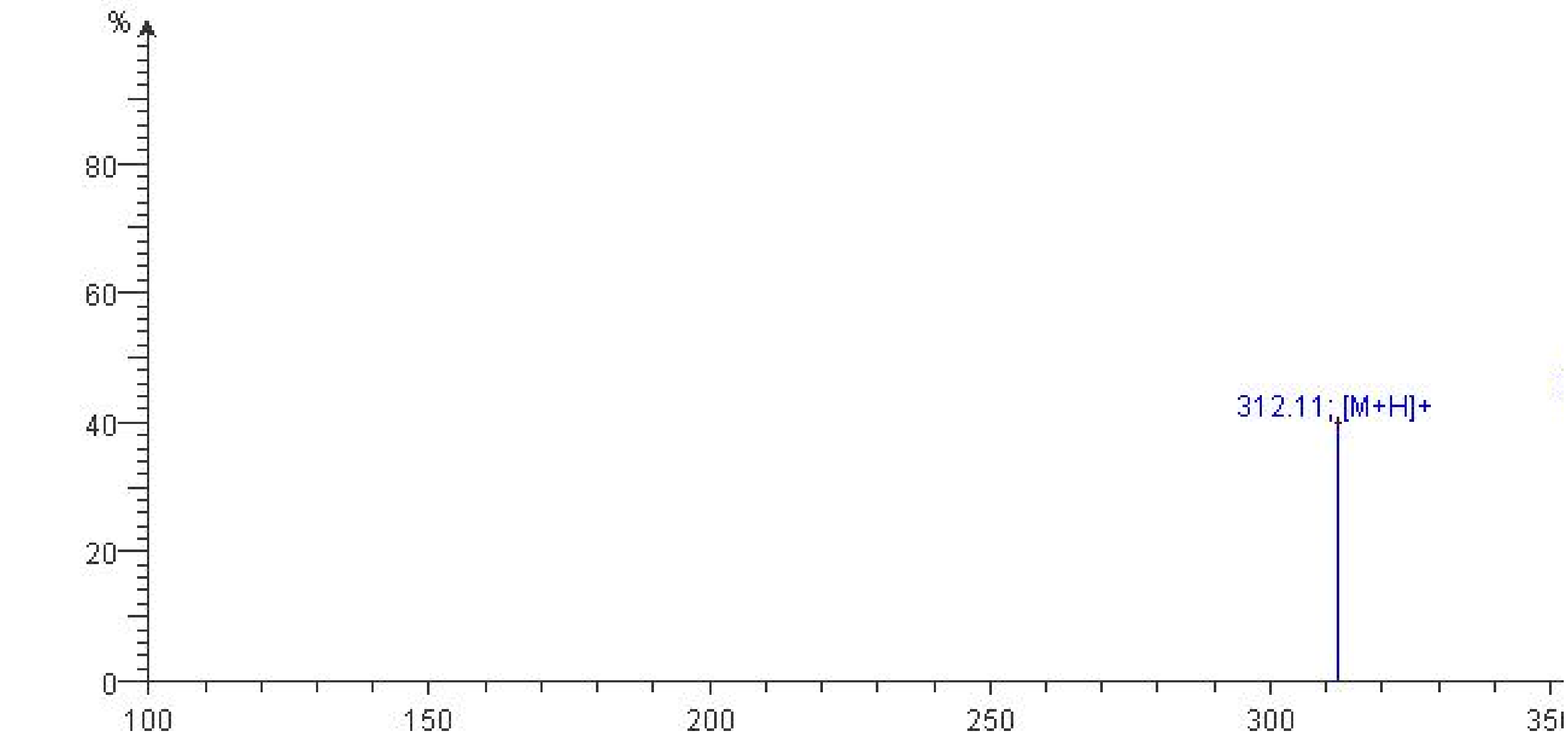

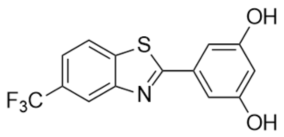

1n

Figure S49. LRMS (ESI+) spectrum of compound **1n.**

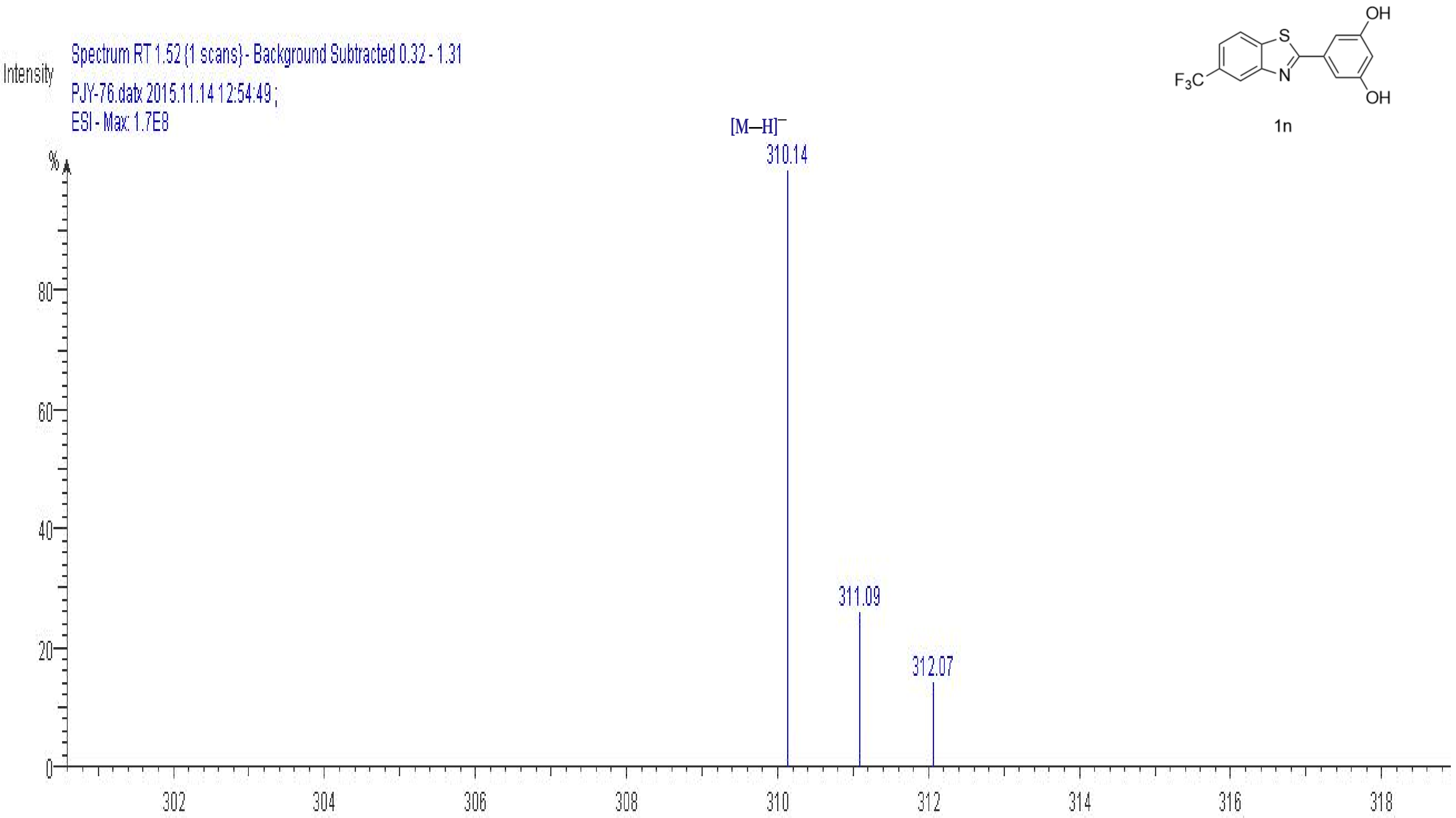

1n

Figure S50. LRMS (ESI–) spectrum of compound **1n.**

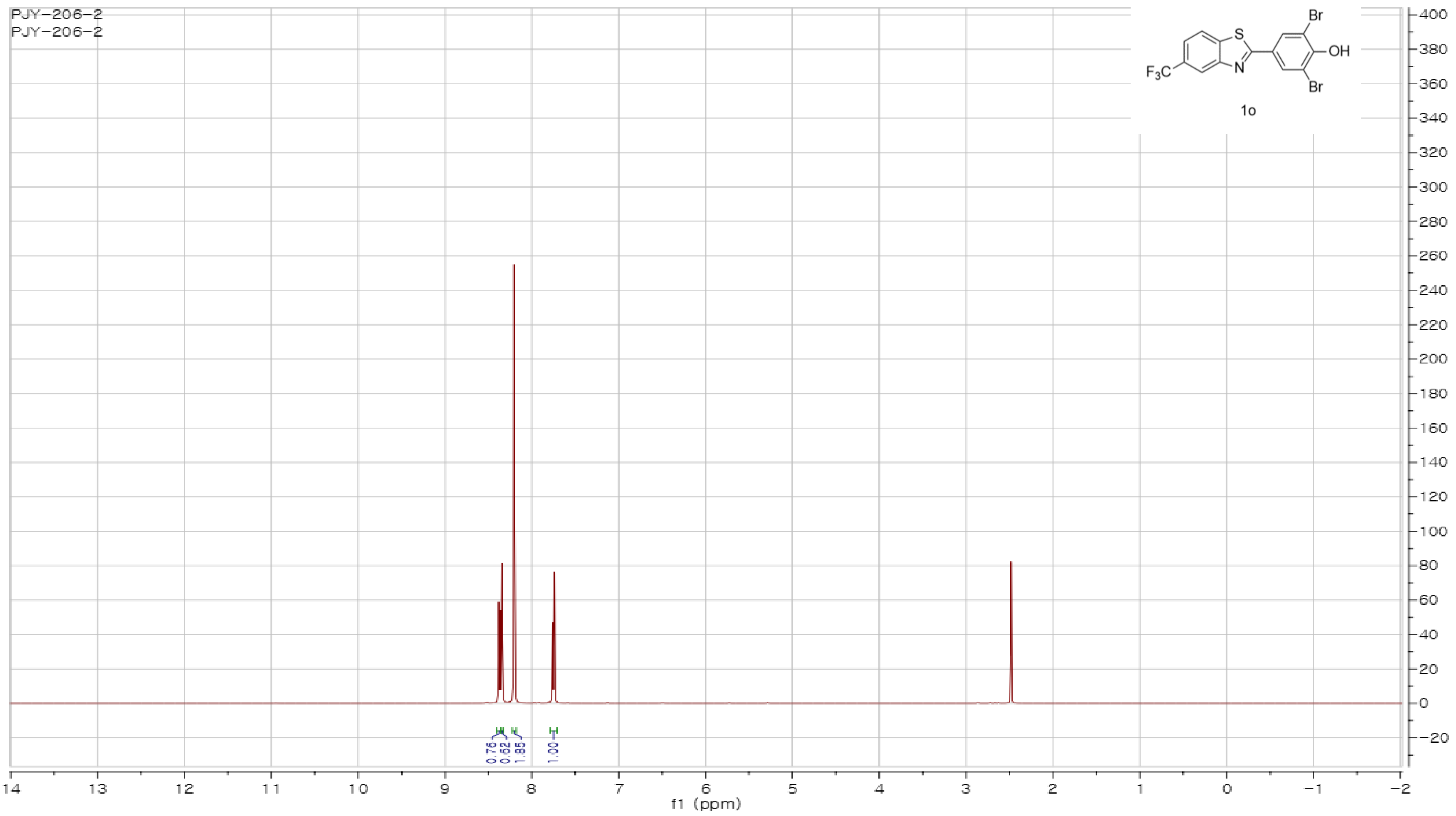

Figure S51. ^{1}H NMR spectrum of compound **1o.**

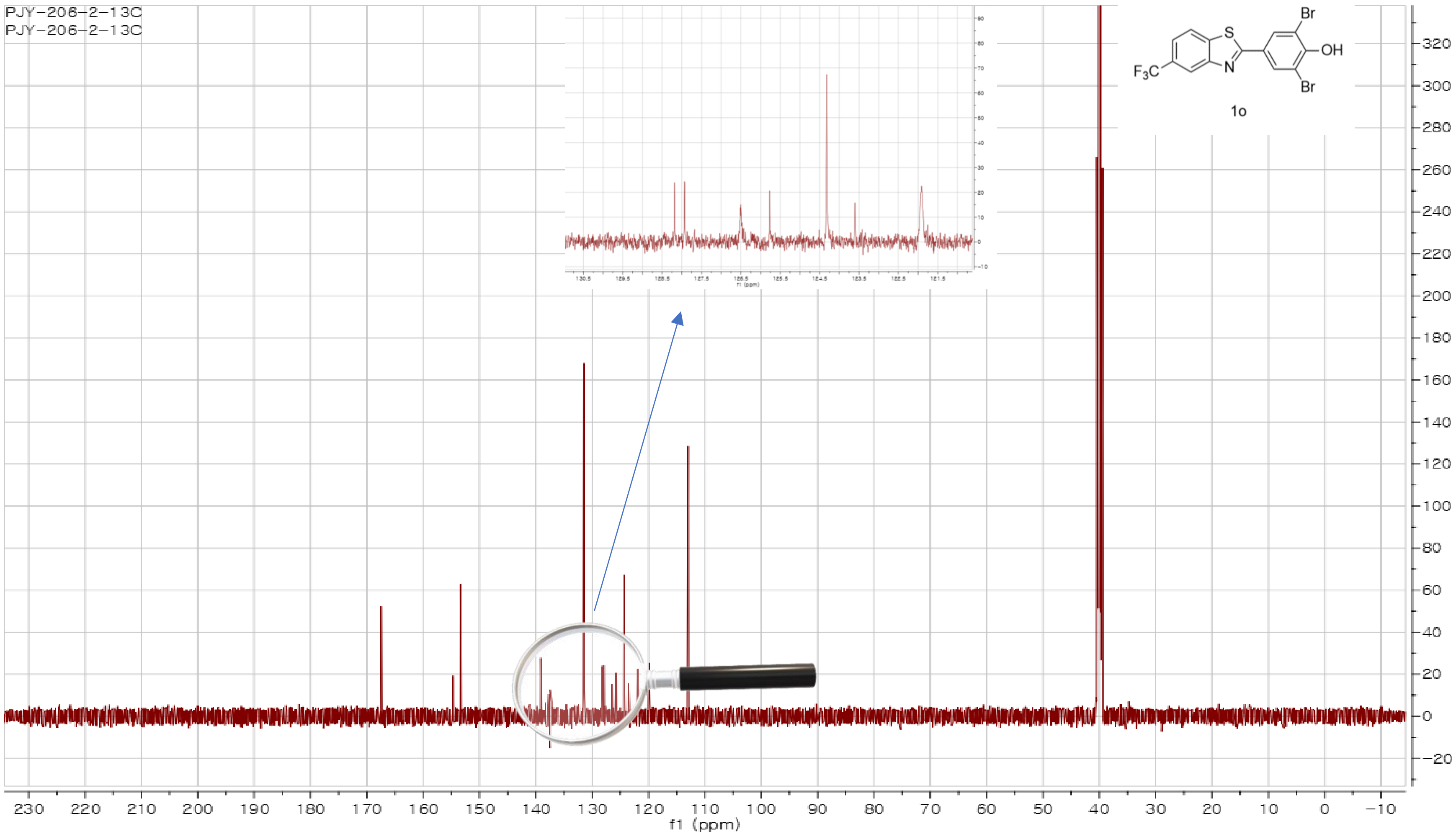

Figure S52. ^{13}C NMR spectrum of compound **1o.**

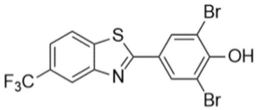

1o

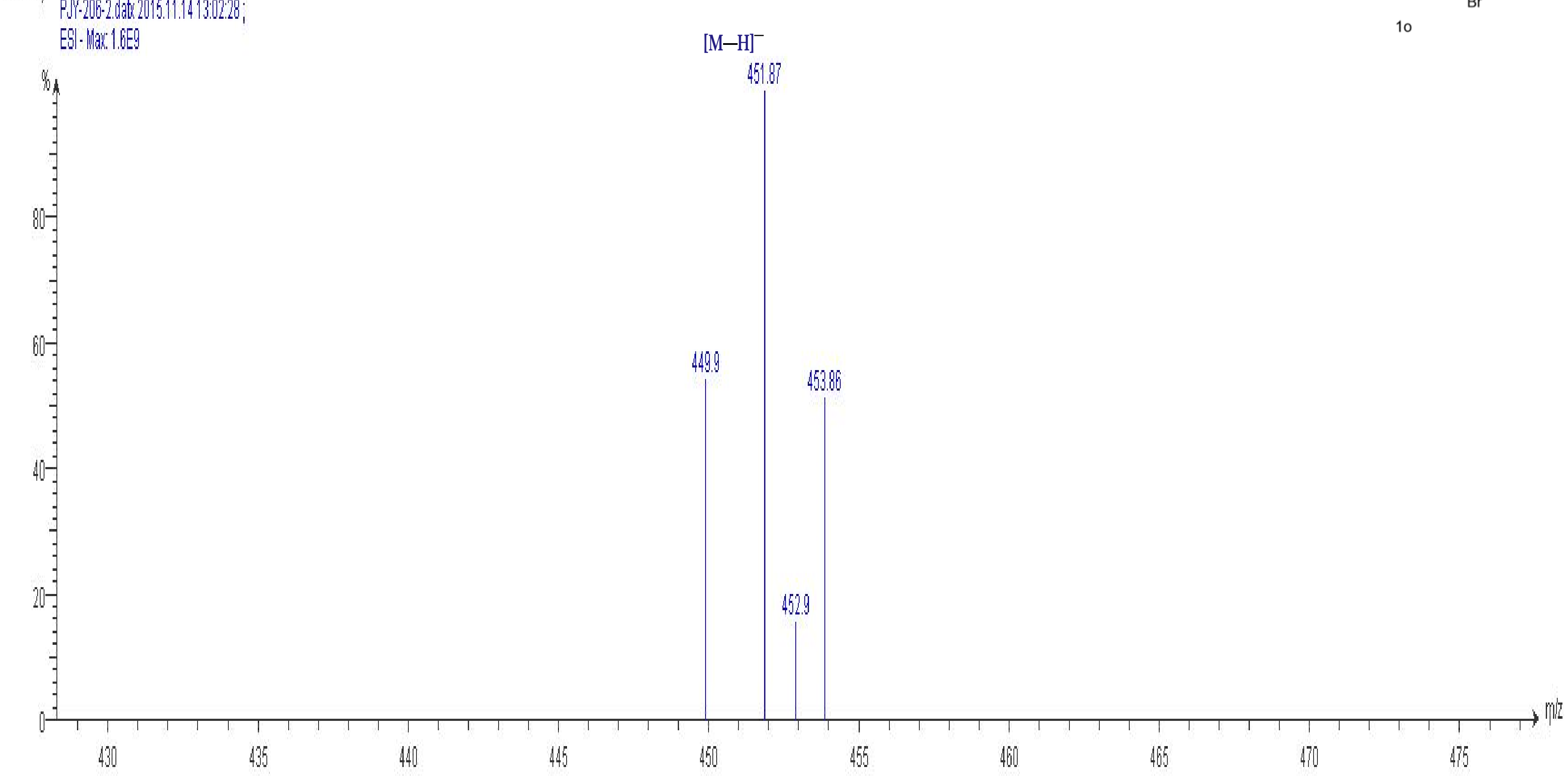

Figure S53. LRMS (ESI–) spectrum of compound **1o.**

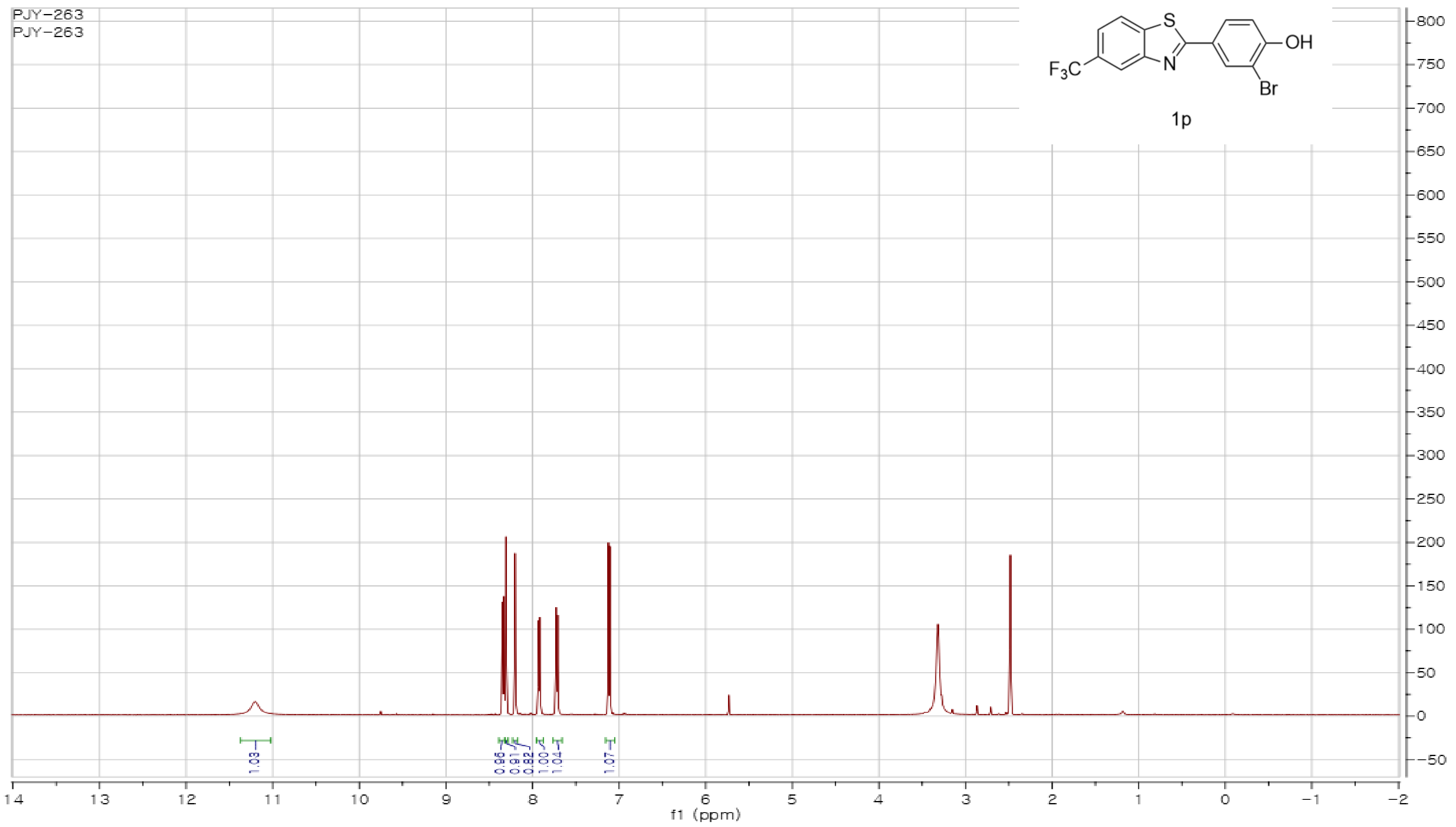

Figure S54. ^{1}H NMR spectrum of compound **1p.**

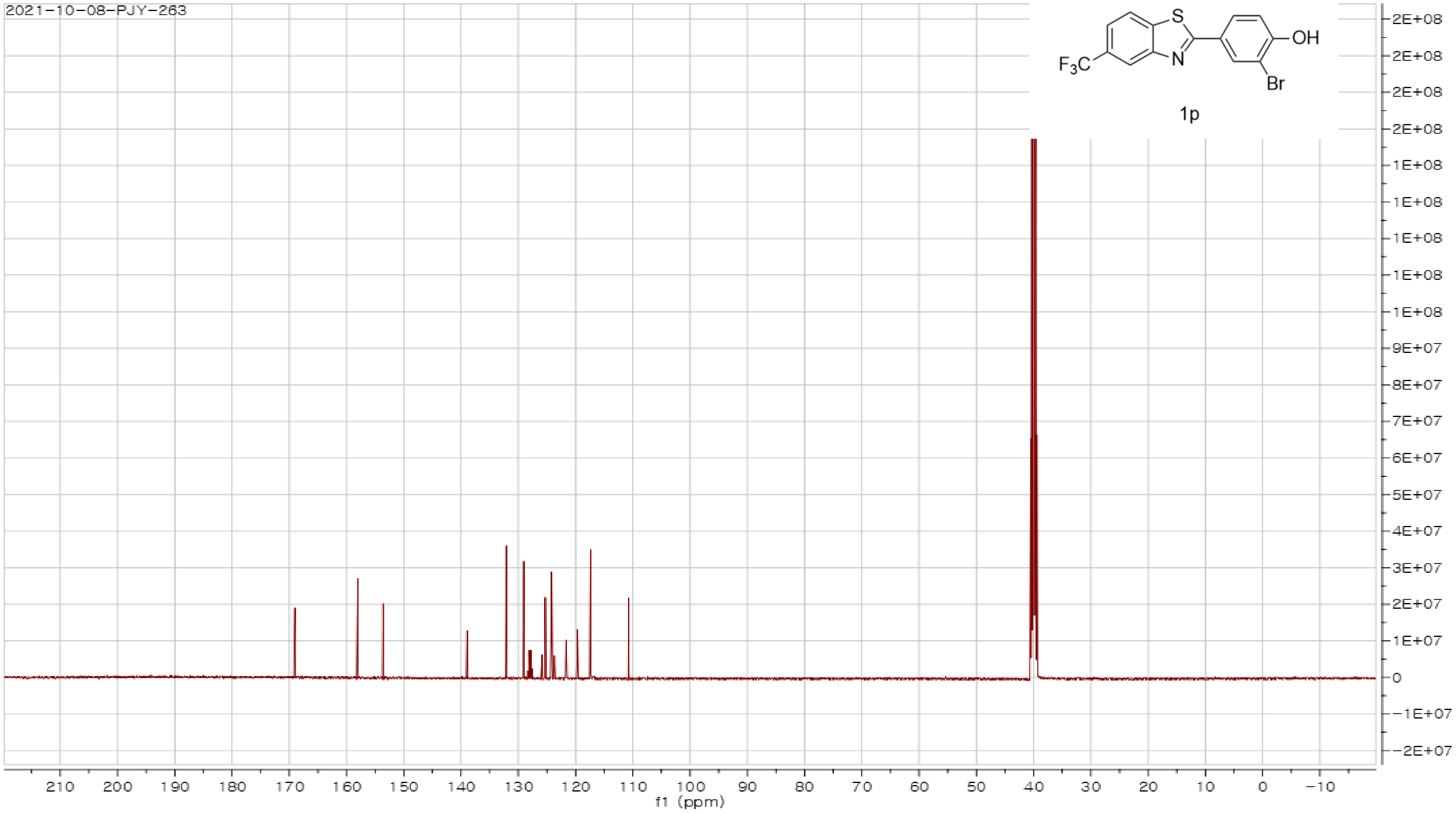

Figure S55. ^{13}C NMR spectrum of compound **1p.**

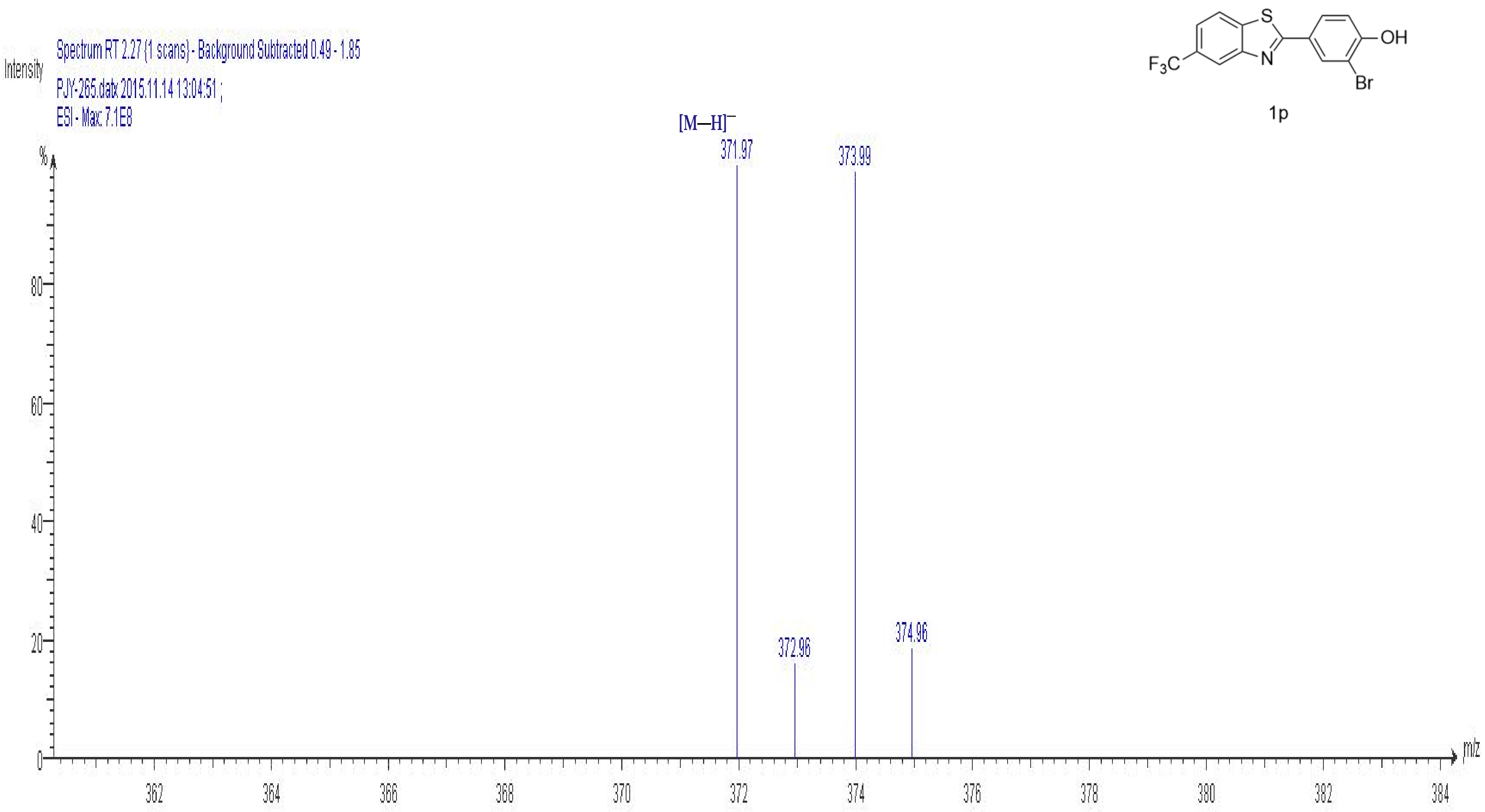

Figure S56. LRMS (ESI–) spectrum of compound **1p.**

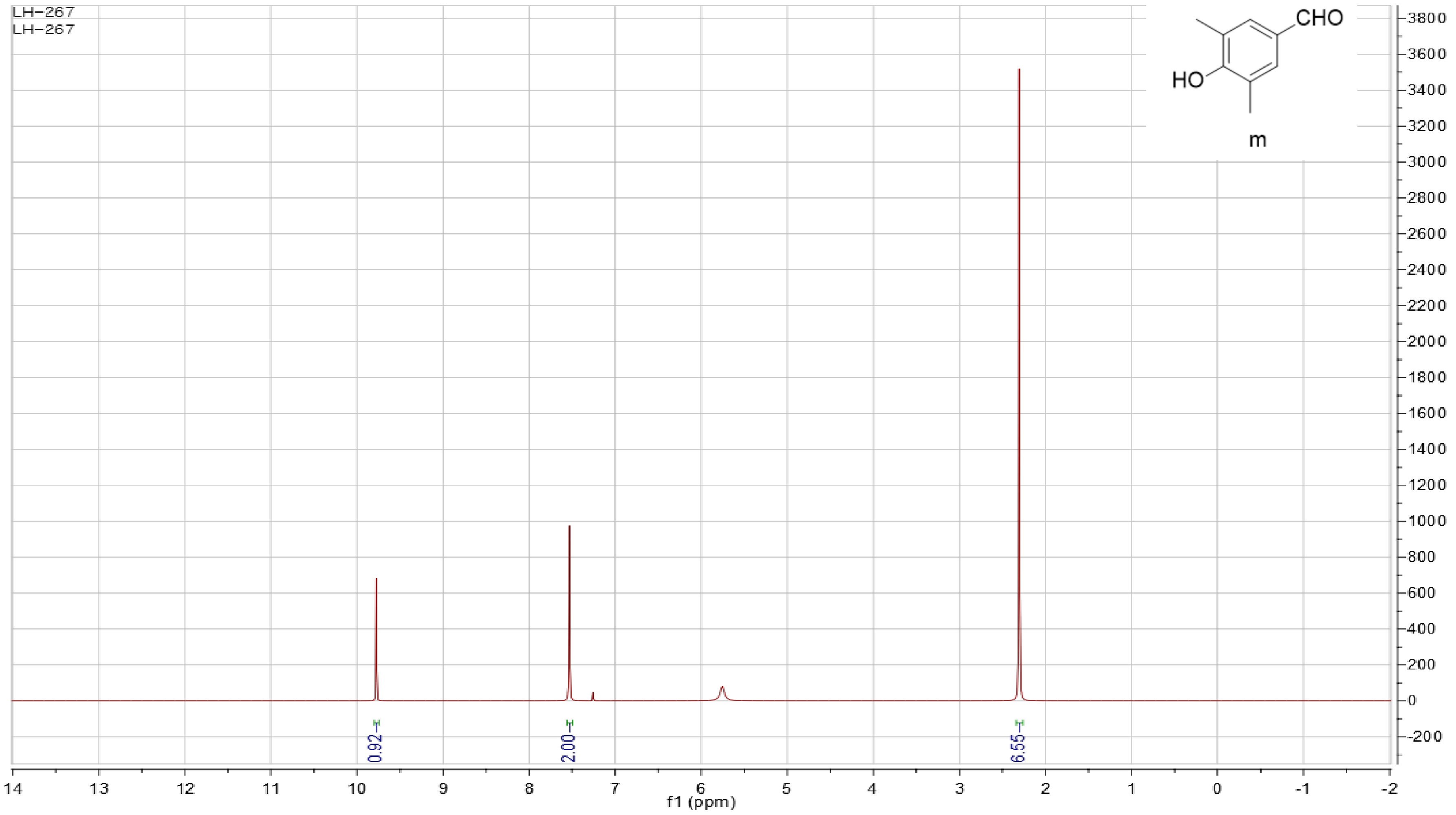

Figure S57. ^{1}H NMR spectrum of compound **m.**

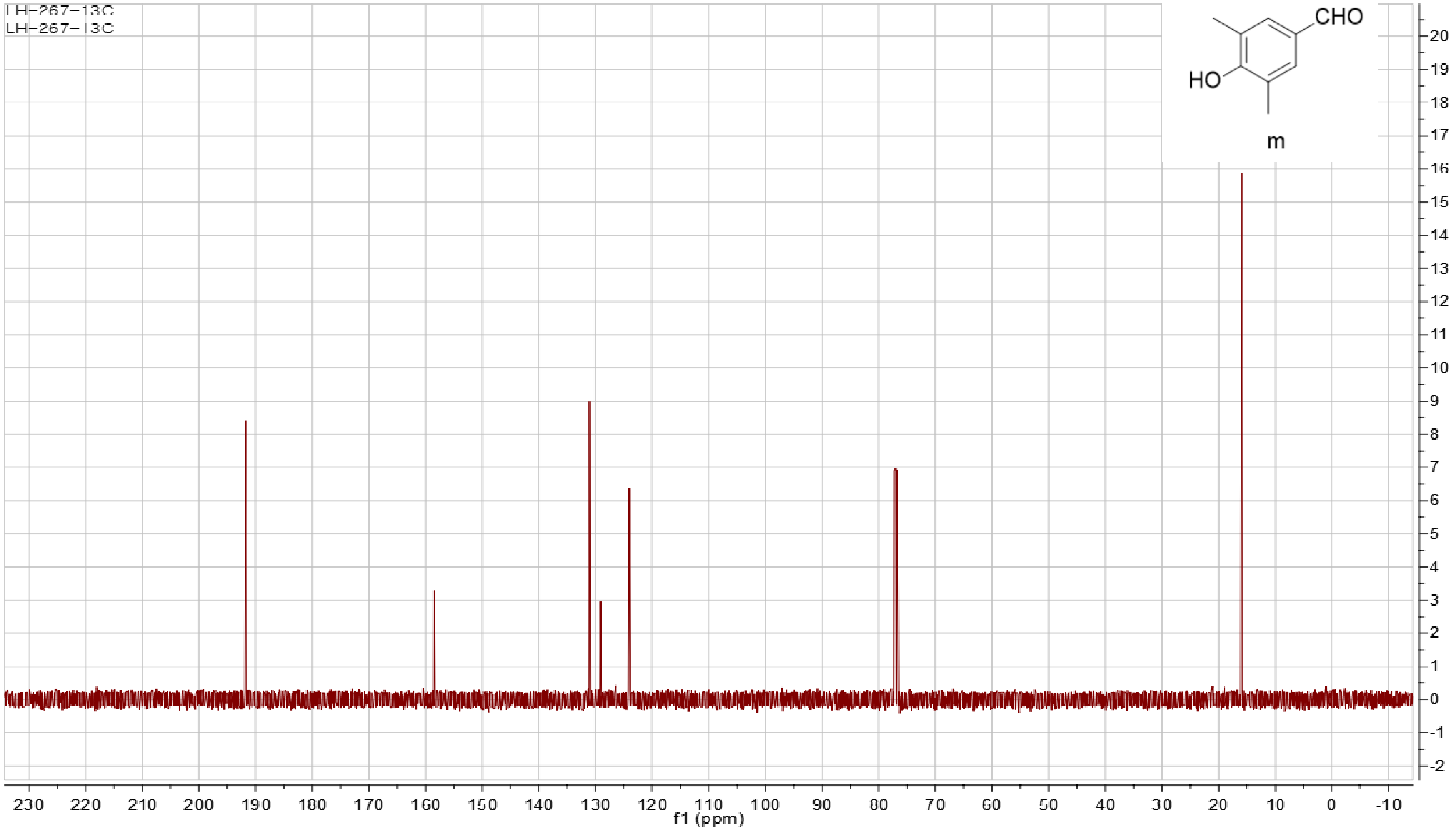

Figure S58. ^{13}C NMR spectrum of compound **m.**

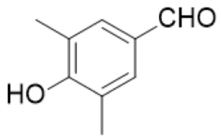

m

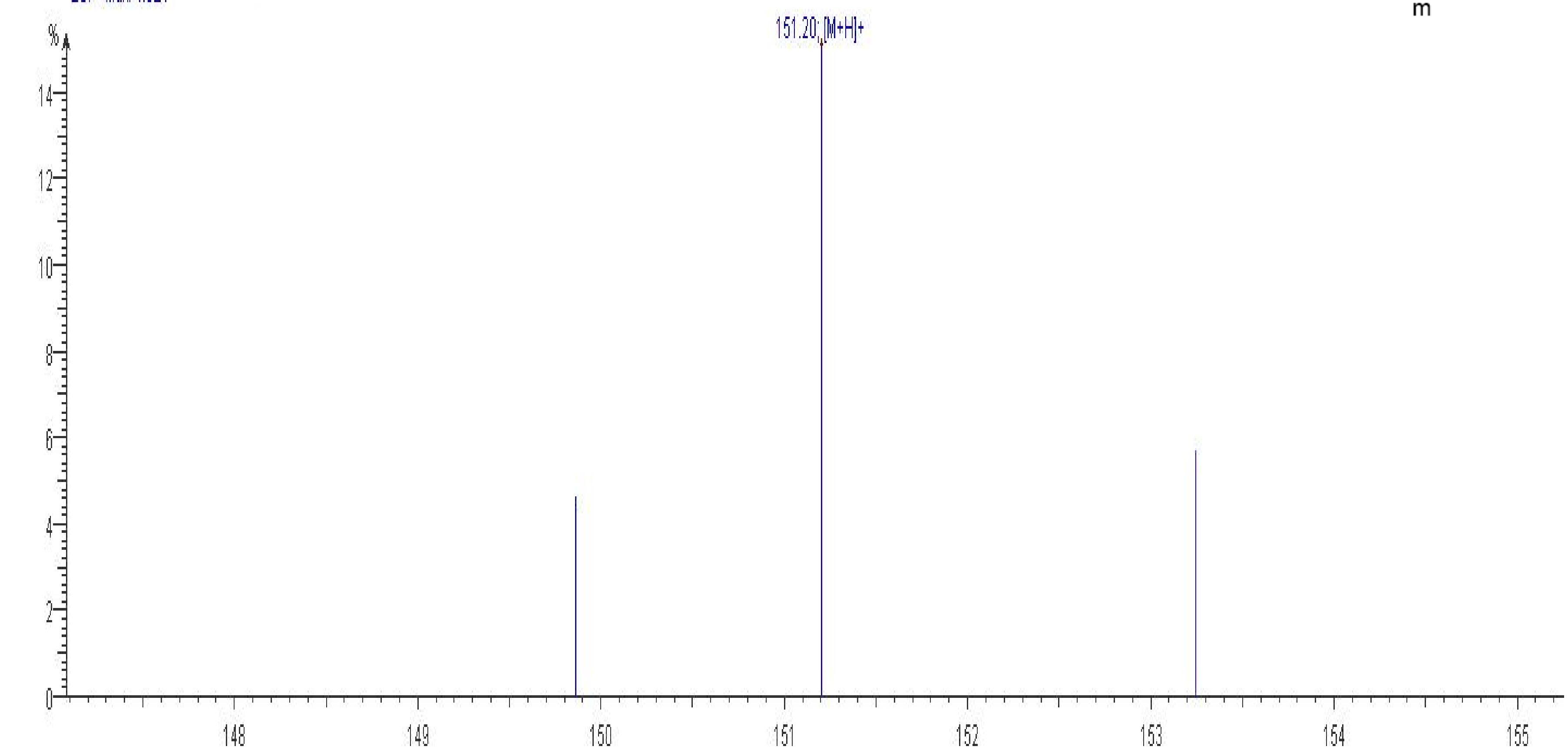

Figure S59. LRMS (ESI+) spectrum of compound **m.**
